# Comparison of statistical models for time-dependent repellency using the novel Pole-dance bioassay against *Tetranychus urticae* Koch

**DOI:** 10.1007/s10493-025-01058-y

**Published:** 2025-08-21

**Authors:** Junho Yoon

**Affiliations:** Research Institute of Agricultural and Life Sciences, Seoul National University, Seoul, 08826 South Korea

**Keywords:** High-throughput, Repellent, Essential oils, Spider mite, Gaussian process

## Abstract

Evaluating the repellency of essential oils against *Tetranychus urticae* requires robust methodologies to detect time-dependent changes in their efficacy, due to differential efficacy, volatility, and sustained effectiveness. This study introduces the Pole-dance bioassay, a novel no-choice method optimized for high-throughput screening of time-dependent repellency, demonstrated through tests on twenty botanical volatiles against *T. urticae*. Four statistical models, probit, two-parameter Hill, four-parameter Hill, and Gaussian Process (GP) regression, were compared for analyzing time-response landing data, employing both experimental results and scenario-based synthetic datasets reflecting diverse curve shapes. GP regression often provided superior model fits, especially for biphasic or incomplete landing trajectories. While repellency rankings based on median effective time (ET_50_) and area under the curve (AUC) were highly correlated within respective models, the choice of model significantly influenced parameter estimates. AUC proved essential for quantifying activity for highly potent compounds where ET_50_ was inestimable (e.g., t-cinnamaldehyde, 1,8-cineole, (-)-terpinen-4-ol, thymol) and offered complementary insights into repellent effects. The Pole-dance bioassay combined with GP modeling establishes an optimized framework for statistically rigorous in vivo high-throughput screening against *T. urticae*.

**Supplementary Information:**

The online version contains supplementary material available at 10.1007/s10493-025-01058-y.

## Introduction

The two-spotted spider mite, *Tetranychus urticae* Koch, poses a significant threat to global agriculture due to its broad host range (Jakubowska et al. 2022; Razuvaeva et al. 2023). Management of this pest has traditionally depended heavily on chemical acaricides, yet this approach is increasingly challenged by environmental safety concerns and rapid development of acaricide resistance (Ilias et al. 2014; Adesanya et al. 2021). Consequently, pest management strategies are progressively incorporating behavioral manipulation tactics, with repellents derived from agricultural sources presenting a potential alternative to conventional chemical pesticides (Isman 2006; Deletre et al. 2016; Oliveira et al. 2018). Among these, plant-derived volatiles are notable candidates, although their effective utilization demands robust methodologies capable of accurately quantifying repellency over time, as their efficacy can diminish due to volatility, chemical degradation, or habituation by target pests (Isman and Miresmailli 2011; Deletre et al. 2016).

Assays previously developed to evaluate repellency in small arthropods, such as *T. urticae*, vary in their design and may yield different interpretations of repellency behavior. Many assay systems often keep the initial inoculation site accessible, allowing mites to move bidirectionally between treated and untreated surfaces. Allowing unrestricted movement may limit the clear tracking of time-dependent repellency patterns. (Dunn et al. 2019; Manu et al. 2021). The bridge assay was developed for this purpose, using a narrow connector between treated and control surfaces to monitor initial directional choice. Although mites typically do not reverse their decision once a surface is chosen, individuals can drop out of observation by escaping downward by silking. No comparable no-choice test has previously been proposed (Snyder et al. 2011; Tak and Isman 2017; Dawood and Snyder 2020). Moreover, conventional assays often emphasize escape responses rather than measuring inhibition of initial host acceptance, a critical behavioral parameter in pest management contexts (Antonious and Snyder 2006; Roh et al. 2012; Faraone et al. 2020).

Conventionally, dose-response or time-response repellency data have been analyzed using models such as probit, logistic, or the Hill equation, as in toxicological or pharmacological contexts (Badolo et al. 2004; World Health Organization 2009; Elamir et al. 2019; Jiang et al. 2019). Nevertheless, uniformly applying a single model type without regard to observed response dynamics can introduce bias, potentially misrepresenting compound efficacy when actual responses deviate from the models (Elamir et al. 2019). The selection of models can critically influence parameter estimation, including key metrics such as median effective time (ET_50_), commonly employed to indicate repellent activity. In addition, the area under the curve (AUC), defined as the integral of the cumulative landing curve over time, was proposed herein as an additional metric. Both metrics can be used to summarize efficacy and rank the potency of candidate repellents.

To facilitate a rigorous evaluation of repellent efficacy, a novel no-choice bioassay named the Pole-dance method was developed, specifically designed for high-throughput screening of repellent compounds against small arthropods such as *T. urticae*. This study explores the impact of model selection on the interpretation of time-dependent repellency data generated using our novel Pole-dance bioassay with *T. urticae*, supplemented by synthetic simulation data. The performance of four distinct modeling frameworks, probit regression, Hill-type equations (two- and four-parameter variants), and non-parametric Bayesian Gaussian Process (GP) regression were compared. Using diverse curve-shape scenarios reflected in synthetic and experimental datasets, model fits through standard metrics (RMSE, MAE, R²) and how model choice affects derived ET_50_ and AUC values, as well as subsequent rankings of compound repellency were assessed. The objective of this study was to provide empirical guidance on selecting appropriate statistical models for analyzing time-dependent repellency data from Pole-dance assay, a novel method to enhance both the methodological rigor and throughput of future repellency studies.

## Materials and methods

### Chemicals

Twenty botanical volatiles used in this study were selected based on previous reports identifying chemicals with repellent activities against *T. urticae* and other arthropods(An and Tak 2022; da Camara et al. 2015; Nerio et al. 2010; Wood et al. 2024; Wu et al. 2022; Yoon and Tak 2018). The selected chemicals included carvacrol (99%), β-caryophyllene (> 98%), (±)-citronellal (> 98.0%), eugenol (99%), geranic acid (> 98%), D-limonene (97%), linalool (> 97%), linalyl acetate (> 98%), α-pinene (98%), α-terpineol (90%), (-)-terpinen-4-ol (> 95.0%), *t*-cinnamaldehyde (99%), citral (> 95%), (+)-borneol (87%), camphor (96%), 1,8-cineole (99%), *t*-anethole (> 98%), thymol (> 98.5%), citronellol (> 98%), and L-menthol (99%).

Carvacrol, eugenol, geranic acid, D-limonene, linalool, α-pinene, α-terpineol, (-)-terpinen-4-ol, (+)-borneol, camphor, 1,8-cineole, thymol, citronellol, and L-menthol were purchased from Sigma-Aldrich (St. Louis, MO, USA). β-Caryophyllene, (±)-citronellal, *t*-cinnamaldehyde, citral, linalyl acetate, and *t*-anethole were obtained from Tokyo Chemical Industry Co., Ltd. (Tokyo, Japan). Analytical grade ethanol (> 99.9%) was acquired from Duksan Pure Chemicals Co., Ltd. (Ansan, South Korea).

## Two-spotted spider mites

The colony of *T. urticae* used in this study was maintained in an insectary at Seoul National University for over 10 years without exposure to any known pesticides. Kidney bean plants (*Phaseolus vulgaris* var. *humilis*) infested with mites were kept under controlled conditions of 25 ± 1 ℃, 70 ± 10% relative humidity, and a photoperiod of 16:8 h (L: D). Kidney beans were purchased from Sohwa Farm (Yecheon, South Korea), and the soil medium (68% coco peat, 15% peat moss, 7% perlite, 4% zeolite) was obtained from Seoul Bio (Eumseong, South Korea). Plants were grown in small plastic pots (Ø 85 × 70 mm), watered twice weekly, and replaced regularly to maintain colony health. Female adult mites less than two weeks old were selected for experimentation.

## Pole-dance assay

**Fig. 1:**
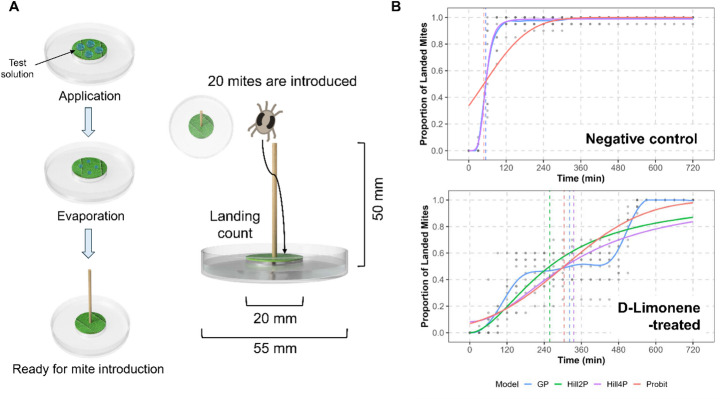
(**A)** Schematic diagram of the Pole Dance method used to assess time-series repellency behavior in spider mites. (**B)** Representative landing trajectories of mites over time under solvent control and D-limonene treated conditions

Leaf discs (Ø 20 mm) were excised from kidney bean leaves and placed on a 2% (w/w) agar medium in Petri dishes (Ø 55 mm), with their abaxial surface facing upward (Fig. 1A). The agar medium was hollowed out to create a recessed area, and water was filled into the surrounding space to form a “leaf island” that prevented mites from escaping. Test compounds were dissolved in ethanol and applied evenly to the abaxial surface of each leaf disc at a dosage of 1 mg per disc using 100 µL of test solution. Following evaporation of the solvent, a wooden toothpick (50 mm length) was vertically impaled at the center of the leaf disc, forming a pole for mite introduction. The tip of the toothpick had a diameter of approximately 2 mm. Twenty adult female mites were then gently placed at the top edge, but they typically moved promptly down onto the shaft of the toothpick, leaving room for additional individuals to be introduced. As a result, most mites stood on the vertical stem rather than remaining clustered at the tip, which minimized crowding and prevented entanglement or accidental falling. This vertical arrangement ensured mites could not reverse their choice (once mites landed, they did not climb back up) nor escape downward using webs. Landing was defined as the moment a mite made sustained physical contact with the leaf disc, remaining continuously for at least 10 s without climbing back onto the pole. Brief or transient contacts shorter than 10 s without direct sustained body-leaf interaction were not counted as successful landings at that moment. The number of mites successfully landing on the leaf disc was recorded at 30-minute intervals over a period of 720 min post-introduction (Fig. 1B). For each compound, the assay was replicated ten times, with each replicate using a separate leaf disc and 20 unique mites (i.e., 200 mites per compound).

## Time-response models

Time-dependent landing data were analyzed using four modeling approaches: probit regression, two-parametric Hill equation (Hill2P), four-parametric Hill equation (Hill4P), and GP regression. For fitting the curves, the cumulative landing data from all replicates were pooled and a single time-response curve was fitted per compound. This approach considered only fixed effects and did not explicitly account for potential inter-replicate variation.

Probit regression modeled the cumulative proportion of mites landed over time, assuming a linear relationship between the probit-transformed response to log time:

$$\:{\varPhi\:}^{-1}\left(p\left(t\right)\right)=\:{\beta\:}_{0}+\:{\beta\:}_{1}\text{l}\text{o}\text{g}\left(t\right)$$

where $\:{\varPhi\:}^{-1}$ is the inverse cumulative distribution function of the standard normal distribution, and p(*t*) is the cumulative proportion of mites landed at time *t*, and *β*_*0*_ and *β*_*1*_ are coefficients.​.

The Hill equation included two variants: a two-parametric version where the minimum and maximum of p(*t*) were fixed, and a four-parametric version where they were estimated from the data. The general form of the Hill equation is:

$$\:p\left(t\right)=\:{E}_{min}+\:\frac{{E}_{max}-\:{E}_{min}}{1+{\left(\frac{{ET}_{50}}{t}\right)}^{h}}$$

where ET_50_ is the time at which 50% of mites were landed, and *h* is the Hill slope parameter indicating the steepness of the curve. For two-parametric variant, E_min_ was fixed to 0.0 and E_max_ to 1.0, assuming that repellency fully inhibits and eventually fully permits landing over time. Fixing these values ensures that ET_50_ corresponds to the time when 50% of mites have landed relative to the total number introduced, regardless of whether this assumption perfectly matches the observed data.

In the four-parametric variant, E_min_, E_max_, *h*, and ET_50_ were simultaneously estimated. Under this flexible fitting, ET_50_ corresponds to the time point at which the cumulative landing reaches 50% of fitted E_max_-E_min_, rather than 50% of the total introduced mites. This allows the model to accommodate situations where complete landing does not occur by the end of the observation period but affects the interpretation of ET_50_ in comparisons among compounds.

Gaussian Process (GP) regression is a flexible, non-parametric Bayesian method useful when the exact form of the relationship between variables is unknown or complex. Unlike parametric models that assume a fixed functional form (e.g., probit or Hill equations), GP regression relies on the correlation between observed data points to smoothly interpolate predictions, effectively accommodating complex and irregular data trajectories

$$\:p\left(t\right)\:\sim\:GP(m\left(t\right),\:k\left(t,\:{t}^{{\prime\:}}\right))$$.

where m(*t*) is the mean function and k(*t*, *t’*) is the covariance kernel function describing the correlation between landing proportion at two time points t and t’. A Matérn 5/2 kernel was adopted as the covariance function, defined as:

$$\:k\left(t,{t}^{{\prime\:}}\right)=\:{\sigma\:}^{2}\:\left(1+\frac{\sqrt{5}\left|t-{t}^{{\prime\:}}\right|}{l}+\:\frac{5{\left(t-{t}^{{\prime\:}}\right)}^{2}}{3{l}^{2}}\right)\text{e}\text{x}\text{p}(-\frac{\sqrt{5}\left|t-{t}^{{\prime\:}}\right|}{l})$$

where *l* is the characteristic length-scale parameter controlling the smoothness of the curve.

## Synthetic simulation data

To evaluate model performance across diverse repellency patterns, synthetic datasets were generated to represent four distinct curve types. Scenarios A and B were generated using a standard parametric Hill equation, while Scenarios C and D were generated using a biphasic model created by integrating two distinct Hill equations. The four types were:

Scenario A: Complete landing with sigmoid shape: Mites gradually land over time, following a sigmoidal trajectory, and ultimately reach 100% cumulative landing.

Scenario B: Incomplete landing with sigmoid shape: Landing behavior follows a simple sigmoid curve but asymptotes below 100% (incomplete landing), representing incomplete repellency decay at the end of the observation period.

Scenario C: Complete landing with biphasic shape: An initial landing phase is followed by a temporary plateau where landing activity ceases, after which a second phase of landing begins, ultimately reaching 100% cumulative landing.

Scenario D: Incomplete landing with biphasic shape: Landing behavior follows the same two-phase pattern as Scenario C, but the final cumulative landing asymptotes below 100% (incomplete landing) at the end of the observation period.

For each of the 20 hypothetical compounds, a unique set of mean parameter values and their corresponding standard deviations were assigned to generate distinct curve characteristics (Supplementary Table S2&S3). To match the experimental bioassay design, ten independent replicate datasets were generated for each compound. This resulted in a total of twenty synthetic compounds, with five representing each of the four scenarios.

### Model evaluation and repellency indicators

Three model fit metrics were used to evaluate how accurately the fitted models matched observed data. Root Mean Square Error (RMSE) measures the average magnitude of prediction errors, penalizing larger errors more heavily due to squaring. Mean Absolute Error (MAE) provides a straightforward average of absolute errors, reflecting typical prediction accuracy without emphasizing large deviations. The coefficient of determination (R²) indicates the proportion of data variability explained by the model, with values closer to 1 indicating better fits.

In addition to fit statistics, two repellency indicator parameters were extracted from each model: the ET_50_ and the area under the curve (AUC). ET_50_ was defined as the time point at which the cumulative proportion of landed mites reached 50%, reflecting the median timing of repellency decay and being particularly sensitive to early-to-mid phase landing dynamics. AUC was defined as the integral of the cumulative landing curve over the entire observation period, capturing the overall degree of repellency loss across time. Lower AUC values indicate slower landing progression and thus stronger sustained repellency throughout the tested period. These two metrics were compared across models and compounds to evaluate how model selection influences the interpretation of repellent activity.

To evaluate how model selection influenced repellency interpretation, rankings of compounds based on ET_50_ and AUC were compared across models. Spearman rank correlation coefficients were calculated to assess the consistency between ET_50_-based and AUC-based rankings within synthetic and experimental datasets. In addition, the degree of ranking diversification across different models was analyzed for ET_50_ and AUC, respectively, to show how model choice affected the perceived potency of candidate repellents.

## Software

Data generation, model fitting, and statistical analyses were performed using R version 4.2.2. The package *GauPro* (version 0.2.5) was used for fitting Gaussian Process regression models. *boot* (version 1.3–28) was used for bootstrapping to estimate parameter distributions and confidence intervals. *pracma* (version 2.3.8) was used for numerical integration to calculate the AUC. For synthetic data generation, *MASS* (version 7.3–58.1) was used for multivariate normal sampling, and *splines* (version 4.2.2) was used for spline generation in biphasic curve shapes. The spearman correlation test was performed by *stats* package (version 4.2.2).

## Results

### Model fitting on experimental data

To evaluate the performance of the four modeling approaches (Probit, Hill2P, Hill4P, and GP) on experimental Pole-dance assay data, model fit statistics including RMSE, MAE, and R² across twenty botanical volatiles and a solvent control were analyzed (Supplementary Table S1). The experimental cumulative landing curves exhibited diversity, including incomplete landing (final cumulative landing < 1.0), non-sigmoidal trajectories with biphasic landing trajectories.

Fitted curves for each compound are presented in Supplementary Fig. S1–S21. During model fitting, several compounds failed to converge under specific models, particularly in Hill2P and Hill4P. Hill2P fitting failed for Estragole, Linalool, and (-)-Terpinen-4-ol, while Hill4P fitting failed for Bornyl acetate, Estragole, Thymol, and (-)-Terpinen-4-ol. In contrast, Probit and GP models successfully generated fits for all compounds without convergence failures.

Multiple-comparison analysis revealed no significant differences among the four models for RMSE (ANOVA, *F*_*3,71*_ = 1.676, *P* = 0.180), MAE (ANOVA, *F*_*3,71*_ = 1.251, *P* = 0.298), or R² (Kruskal–Wallis, *H*_*3*_ = 4.022, *P* = 0.259) when fitting the experimental time-dependent landing data (Table 1).

**Table 1:** For experimental data and each synthetic data scenario, RMSE, MAE, and R² were compared across probit, Hill2P, Hill4P, and GP. Following normality check with Shapiro-Wilk test, overall differences were tested with one-way ANOVA (parametric) or Kruskal-Wallis (non-parametric). Multiple comparisons were made by tukey’s test (parametric) or dunn’s test (non-parametric) at α of 0.05

Data source	Data group	Metric	Test used	Test statistics (df)		*P*	Pairwise comparison			
							Probit	Hill2P	Hill4P	GP
Experimental	All compounds	RMSE	ANOVA	1.6761	(*F*_*3,71*_)	0.1799	0.1238^a†‡^	0.1190^a^	0.1204^a^	0.1026^a^
Experimental	All compounds	MAE	ANOVA	1.2505	(*F*_*3,71*_)	0.298	0.0798^a^	0.0776^a^	0.0810^a^	0.0629^a^
Experimental	All compounds	R^2^	Kruskal-Wallis	4.0219	(*H*_*3*_)	0.2591	0.7388^a^	0.8225^a^	0.8490^a^	0.8477^a^
Synthetic	Scenario A	RMSE	ANOVA	0.4043	(*F*_*3,16*_)	0.7519	0.0924^a^	0.0924^a^	0.0923^a^	0.0918^a^
Synthetic	Scenario A	MAE	ANOVA	0.1706	(*F*_*3,16*_)	0.9147	0.0574^a^	0.0574^a^	0.0579^a^	0.0569^a^
Synthetic	Scenario A	R^2^	ANOVA	0.5087	(*F*_*3,16*_)	0.6819	0.9418^a^	0.9418^a^	0.9420^a^	0.9424^a^
Synthetic	Scenario B	RMSE	ANOVA	3.6738	(*F*_*3,15*_)	0.0364	0.1499^a^	0.1218^a^	0.0897^a^	0.0928^a^
Synthetic	Scenario B	MAE	ANOVA	2.0067	(*F*_*3,15*_)	0.1563	0.1118^a^	0.0872^a^	0.0657^a^	0.0615^a^
Synthetic	Scenario B	R^2^	Kruskal-Wallis	7.2695	(*H*_*3*_)	0.0638	0.8561^a^	0.8775^a^	0.8968^a^	0.8985^a^
Synthetic	Scenario C	RMSE	ANOVA	8.6213	(*F*_*3,16*_)	0.0012	0.1473^a^	0.1573^a^	0.1441^a^	0.1010^b^
Synthetic	Scenario C	MAE	ANOVA	8.4579	(*F*_*3,16*_)	0.0014	0.1091^a^	0.1248^a^	0.1102^a^	0.0721^b^
Synthetic	Scenario C	R^2^	ANOVA	3.4608	(*F*_*3,16*_)	0.0414	0.8141^a^	0.7972^b^	0.8377^a^	0.9115^a^
Synthetic	Scenario D	RMSE	Kruskal-Wallis	6.0857	(*H*_*3*_)	0.1075	0.1273^a^	0.1295^a^	0.1289^a^	0.1018^a^
Synthetic	Scenario D	MAE	Kruskal-Wallis	5.3086	(*H*_*3*_)	0.1505	0.1035^a^	0.0983^a^	0.1029^a^	0.0811^a^
Synthetic	Scenario D	R^2^	ANOVA	0.9196	(*F*_*3,16*_)	0.4537	0.7409^a^	0.7777^a^	0.7945^a^	0.8361^a^
Synthetic	Scenario A + B + C + D	RMSE	ANOVA	6.1238	(*F*_*3,75*_)	0.0009	0.1341^a^	0.1238^a^	0.1128^b^	0.0982^b^
Synthetic	Scenario A + B + C + D	MAE	ANOVA	4.0312	(*F*_*3,75*_)	0.0103	0.0988^a^	0.0913^b^	0.0826^b^	0.0683^b^
Synthetic	Scenario A + B + C + D	R^2^	Kruskal-Wallis	8.9843	(*F*_*3,75*_)	0.0295	0.8449^a^	0.8698^b^	0.8937^b^	0.9178^b^

† Each cell displays either the mean (when ANOVA was used) or the median (when Kruskal–Wallis test was used), depending on the test used

‡ Within each row, models that are assigned different uppercase letters in the pairwise comparison column differ significantly at α = 0.05

### Model fitting on synthetic data

To systematically evaluate model performance, Probit, Hill2P, Hill4P, and GP models were applied to synthetic datasets simulating four distinct repellency curve types. These scenarios included complete landing with a sigmoidal shape (Scenario A), incomplete landing with a sigmoidal shape (Scenario B), complete landing with a biphasic shape (Scenario C), and incomplete landing with a biphasic shape (Scenario D).

Model fitting results revealed scenario-specific differences in model performance (Table 1, Supplementary Table S4 & Fig. S22-S31). In Scenarios A, B, and D, no statistical differences were observed among the four models based on RMSE, MAE, or R² values. In Scenario C, however, GP achieved significantly better fits compared to Probit, Hill2P, and Hill4P. Specifically, for RMSE in Scenario C, ANOVA detected a significant difference among models (*F*_*3,16*_ = 6.542, *P* = 0.0042), and Tukey’s post-hoc indicated that GP had significantly lower RMSE compared to Probit (*P* = 0.016), Hill2P (*P* = 0.009), and Hill4P (*P* = 0.007). For MAE, ANOVA similarly revealed a significant difference (*F*_*3,16*_ = 7.103, *P* = 0.0031), with GP outperforming Probit (*P* = 0.012), Hill2P (*P* = 0.008), and Hill4P (*P* = 0.006) in post-hoc comparisons. For R², a significant difference was found by Kruskal-Wallis analysis (*H*_*3*_ = 9.204, *P* = 0.0268), and Dunn’s post-hoc tests showed that GP achieved significantly higher R² values compared to Probit (*P* = 0.034), Hill2P (*P* = 0.022), and Hill4P (*P* = 0.019).

When all dataset from four synthetic scenarios were aggregated, significant differences among models for RMSE (ANOVA, *F*_*3,75*_ = 6.124, *P* = 0.0009), MAE (ANOVA, *F*_*3,75*_ = 4.031, *P* = 0.010), and R² (Kruskal–Wallis, *H*_*3*_ = 8.984, *P* = 0.030) were detected. Tukey’s HSD was applied for RMSE and MAE, and Dunn’s test for R². For RMSE, Hill4P and GP performed significantly better (*P* < 0.05). For MAE, Probit performed significantly poorer than Hill2P, Hill4P, and GP. For R², same trend was observed to MAE, where Probit’s performance significantly poorer than others. These results indicate that Probit consistently underperformed when multiple landing trajectory scenarios presence together.

### Ranking concordance across repellency metrices

To assess the consistency of compound rankings based on different repellency indices, rankings derived from ET_50_ and AUC values for each model using Spearman rank correlation analysis were compared.

**Fig. 2:**
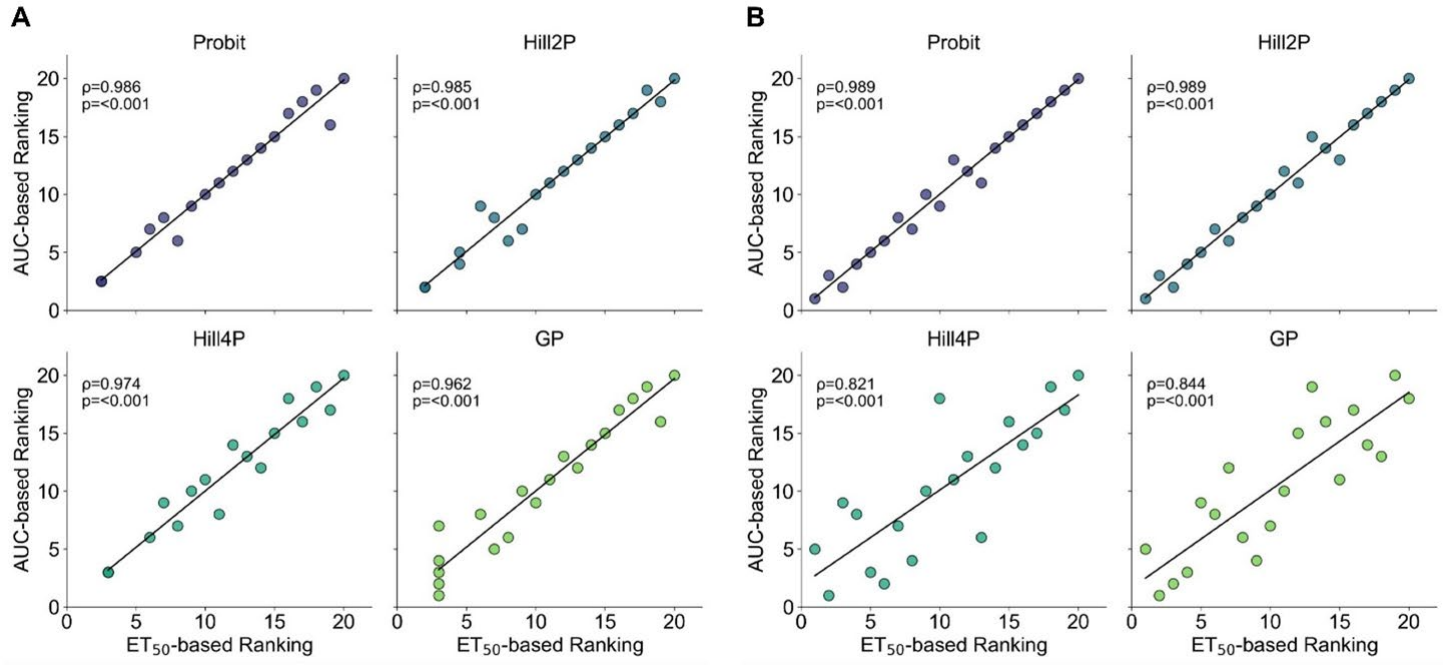
Comparison of repellency rankings derived from ET_50_ and AUC metrics. Spearman rank correlations were calculated for experimental data (**A**) and synthetic data (**B**), assessing the consistency between the two indices

In experimental datasets, a similarly high degree of concordance was observed across all models. Spearman correlation coefficients were 0.986 for Probit (*P* < 0.001), 0.985 for Hill2P (*P* < 0.001), 0.974 for Hill4P (*P* < 0.001), and 0.962 for GP (*P* < 0.001) (Fig. 2A). These results indicate that ET_50_- and AUC-based rankings were highly consistent regardless of the modeling approach, with particularly strong alignment across all models in the experimental datasets.

A similarly high level of concordance was observed in the synthetic datasets. Spearman correlation coefficients were 0.989 for Probit (*P* < 0.001), 0.989 for Hill2P (*P* < 0.001), 0.821 for Hill4P (*P* = 0.0001), and 0.844 for GP (*P* < 0.001) (Fig. 2B).

### Repellent activity in experimental data and ranking concordance across models

**Fig. 3:**
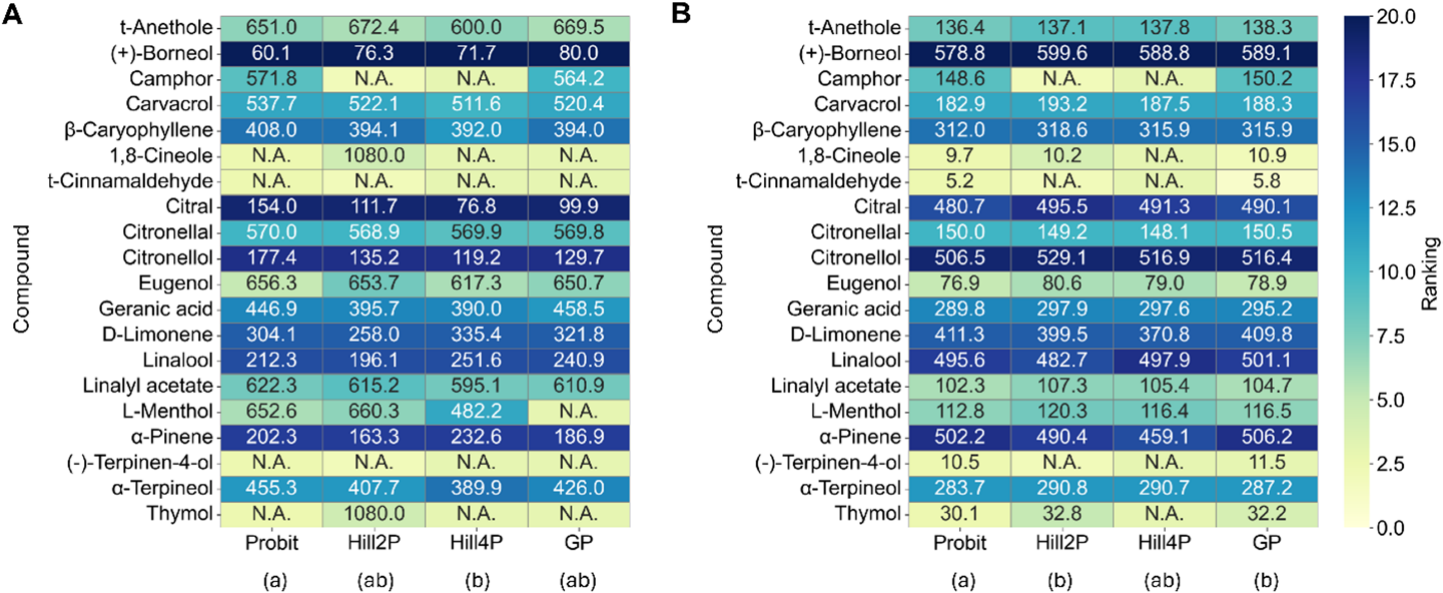
Model-dependent diversification of compound rankings in experimental data based on ET_50_ (A) and AUC (B). Each cell shows the estimated mean value; missing values due to model non-convergence are displayed as N.A. However, these non-converged cases are still ranked based on their presumed relative potency (i.e., being the most or least potent), and are included accordingly in the ranking color scale. Rankings were statistically compared using Friedman’s test (α = 0.05), followed by Nemenyi’s post-hoc test. Statistically distinct model groups are indicated by different letters in parentheses beneath each model label

To evaluate differences in estimated repellent activity across models, ET_50_ and AUC obtained from the experimental datasets were analyzed. In ET_50_-based comparisons, Friedman test revealed statistically significant differences among models (*χ²* = 7.9714, *P* = 0.0466). Post-hoc Nemenyi tests indicated that Hill4P differed significantly from Probit (*P* = 0.0175), whereas no significant differences were observed among the other model pairs (Fig. 3A).

Similarly, AUC-based comparisons demonstrated significant differences across models (Friedman test, *χ²* = 12.2571, *P* = 0.0066). Post-hoc analyses revealed that Hill2P differed significantly from Probit (*P* = 0.0020), and GP also differed significantly from Probit (*P* = 0.0438) (Fig. 3B).

### Repellent activity in synthetic data and ranking concordance across models

**Fig. 4:**
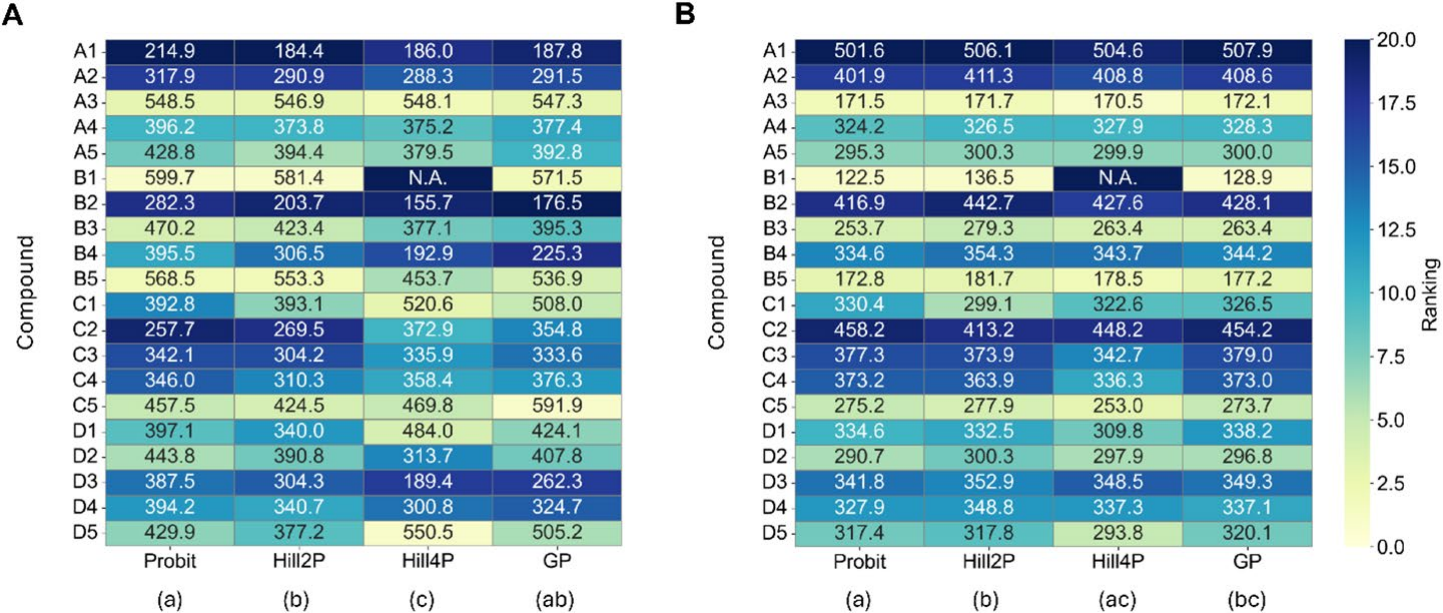
Model-dependent diversification of compound rankings in synthetic data based on ET_50_ (A) and AUC (B). Each cell shows the estimated mean value; missing values due to model non-convergence are displayed as N.A. However, these non-converged cases are still ranked based on their presumed relative potency (i.e., being the most or least potent), and are included accordingly in the ranking color scale. Rankings were statistically compared using Friedman’s test (α = 0.05), followed by Nemenyi’s post-hoc test. Statistically distinct model groups are indicated by different letters in parentheses beneath each model label

To assess whether similar trends held under simulated conditions, the synthetic datasets were analyzed. Although the synthetic datasets were generated from four distinct scenario types (A to D), they were collectively analyzed in this section to evaluate how each model performs across a diverse range of curve shapes and landing dynamics, mirroring the variability observed in experimental data. To compare the estimated repellent activity across different modeling approaches, the ET_50_ and AUC values obtained from synthetic datasets were analyzed. In ET_50_-based comparisons, Friedman test revealed statistically significant differences among models (*χ*^*2*^ = 11.6526, *P* = 0.0087). Post-hoc Nemenyi tests indicated that Hill2P differed significantly from Probit (*P* = 0.0047) and Hill4P also differed significantly from Probit (*P* = 0.0272), whereas no significant differences were observed between GP and the other models (Fig. 4A).

Similarly, AUC-based comparisons demonstrated significant differences across models (Friedman test, *χ*^*2*^ = 14.0526, *P* = 0.0028). Post-hoc analyses revealed that Hill2P differed significantly from both Probit (*P* = 0.0118) and Hill4P (*P* = 0.0272), while GP showed a significant difference from Probit (*P* = 0.0272) (Fig. 4B).

### Comparative repellent activities of botanical volatiles

These comparisons relied exclusively on the Gaussian-process (GP) model because (i) it can generate a reliable curve for every compound unless the end-point landing proportion remains below 50% (in which case ET_50_ is, by definition, inestimable) and (ii) across the experimental dataset the GP model yielded the lowest RMSE and MAE and the highest R², outperforming Probit, Hill2P, and Hill4P.

For ET_50_ values, the solvent control landed earliest, with an ET_50_ of 54.5 (95% CI: 47.1–63.7) min. Its 95% CI overlapped with that of (+)-borneol, which had ET_50_ of 80.00 (62.95–94.35) min, indicating no detectable repellent activity. In contrast, five compounds, 1,8-cineole, t-cinnamaldehyde, L-menthol, (-)-terpinen-4-ol, and thymol, never reached 50% cumulative landing, so ET_50_ could not be calculated. These were considered as the strongest repellents, although no comparison among them was available (Fig. 3A & Supplementary Table S1).

Among volatiles with calculable ET_50s_, t-anethole was the most potent, with an ET_50_ of 669.5 (628.8–708.0) min. Its 95% CI overlapped that of eugenol, which had an ET_50_ of 650.7 (629.1–667.2) min, marking these two as the strongest repellents among the compounds with quantified ET_50_s (Fig. 3A & Supplementary Table S1).

For AUC, the control produced the largest value, 653.5 (631.8–655.0) as expected. Unlike when compared based on ET_50_s, (+)-Borneol showed significant repellency with an AUC of 589.1 (568.2–589.2), whose interval did not overlap that of the control. Unlike ET_50_, AUCs were calculated for every compound regardless of end-point landing proportion. The smallest AUC, and therefore strongest protection, was recorded for t-cinnamaldehyde at 5.8 (5.0–5.8), followed by 1,8-cineole at 10.9 (9.4–10.9) and (-)-terpinen-4-ol at 10.9 (9.4–10.9).

## Discussion

This study introduced the Pole-dance bioassay, a novel no-choice method for assessing time-dependent repellent effects against *T. urticae*. The performance of four distinct statistical models, probit, Hill2P, Hill4P, and GP for analyzing the resulting time-response data were compared. Our findings demonstrate that while all models can capture basic repellent dynamics, GP regression offers superior flexibility and fit, particularly for experimental data exhibiting complex trajectory shapes or incomplete landing at the end-point. Furthermore, ET_50_ and AUC values were used as indicators for repellent activities.

The Pole-dance bioassay was developed to address limitations inherent in existing repellency testing methodologies. Many conventional assays, including choice tests, can be confounded by factors such as mite indecision or reversible movements between treated and untreated zones, complicating the interpretation of true repellency (Dunn et al. 2019; Manu et al. 2021). While no-choice assays like the bridge assay exist, our Pole-dance method might offer an advantage by preventing mites from returning to the introduction point or escaping via webbing, ensuring that observed movement onto the treated surface directly reflects the decay of repellency over time (Snyder et al. 2011; Tak and Isman 2017; Dawood and Snyder 2020). This setup specifically measures the inhibition of host acceptance rather than escape responses, a potential factor in evaluating practical pest management potential.

Accurate quantification of time-dependent repellency relies on appropriate statistical modeling. Traditional models like probit and Hill equations, assume specific curve shapes (Elamir et al. 2019). However, biological responses, especially behavioral ones can deviate significantly from these curves, potentially leading to biased parameter estimates and misinterpretation of compound efficacy if models are applied rigidly. Our results highlight this challenge: while probit and Hill models performed adequately in simple sigmoidal scenarios, they struggled to fit complex landing patterns observed experimentally and in synthetic Scenario C, sometimes failing to converge. Furthermore, while the Hill4P offers flexibility by estimating the minimum (E_min_) and maximum (E_max_) landing proportions, its ET_50_ parameter represents the time to reach 50% of the fitted range (E_max_ −E_min_), not necessarily 50% of the total mites landed. Caution must therefore be exercised when comparing Hill4P-derived ET_50_ values, especially if E_max_​ is substantially below 1.0, as it reflects a different conceptual point than the ET_50_ from models assuming a 0.0–1.0 range (Jiang et al. 2019). In contrast, the GP demonstrated flexibility, accommodating diverse curve shapes, including incomplete landing and fluctuations in landing rates.

As well as assessing for fit statistics, Two key metrics were used to quantify repellency, ET_50_ and AUC. ET_50_ provides an intuitive measure of the median-based tendency of repellency decay, sensitive to early-to-mid phase dynamics. AUC, conversely, captures the overall repellency effect across the entire observation period, with lower values indicating stronger, more sustained repellency. Our analysis revealed a strong and statistically significant positive correlation between rankings derived from ET_50_ and AUC across all models for both experimental and synthetic data. Notably, in the synthetic dataset, the Spearman correlation coefficients for Hill4P (ρ = 0.821) and GP (ρ = 0.844) were slightly lower than those for Probit and Hill2P (both ρ = 0.989). This modest reduction in coefficient may reflect the greater sensitivity of Hill4P and GP models in fitting diverse curve shapes, which could lead to differential ordering of ET50s across compounds. This may be because ET_50_ values are more sensitive to curve type. For example, in incomplete landing scenarios, estimated ET_50_ in Hill4P model is not the true 50% of total introduced individuals, but the 50% of asymptotic final landing proportion. Consequently, the ET_50_ may represent a different biological meaning than in models that assume complete landing, reducing its alignment with AUC-based rankings. Overall, however, all models still demonstrated broadly consistent rankings, indicating that both metrics generally identify similar trends in repellency potency across compounds.

Nevertheless, the choice of metric could influence conclusions about whether a compound exhibits statistically significant repellent activity compared to the control, based on 95% CI overlap. For instance, although (+)-Borneol ranked among the least potent compounds by both metrics, its 95% CI for ET_50_ overlapped with that of the solvent control, suggesting no significant repellency based on this metric. In contrast, its AUC value’s 95% CI did not overlap with the control’s, indicating significant repellency when assessed over the entire time course. This exemplifies how ET_50_ and AUC can provide complementary information for screening potential repellents, in spite of their ranking concordances.

​ A key advantage of using AUC is its universal applicability across all compounds tested. Unlike ET_50_, which is not able to be defined if fewer than 50% of mites landed during the observation period, AUC provides a quantifiable measure of repellency regardless of the ultimate landing percentage. This proved essential in our study for evaluating and ranking the most potent repellents, such as t-cinnamaldehyde, 1,8-cineole, (-)-terpinen-4-ol, and thymol, for which ET_50_ values could not be determined due to their strong effects.

The relative importance of ET_50_ versus AUC may depend on the intended use context of the repellent. A high ET_50_ suggests a strong initial barrier effect, where host contact is delayed for an extended period. This may be valuable for short-term prevention of invasion or entry deterrence, such as in contact repellency (Luker 2024). However, for clearer interpretation in such contexts, alternative performance metrics like ET_20_ or complete protection time may be more appropriate (Fradin and Day, 2002; Paluch, 2009). On the other hand, a low AUC emphasizes the overall persistence of repellency across the observation period. Notably, lower AUC values can arise not only from a low final landing rate but also from a delayed onset of cumulative landing that peaks at later time points, cases in which ET_50_ alone may be less informative. AUC therefore provide a more comprehensive indicator of repellency durability over time. In practice, this would be particularly beneficial for applications requiring long-term protection, such as spatial repellency in agricultural settings (Achee et al. 2012; Deletre et al. 2016). Both metrics offer distinct but complementary perspectives and should be interpreted in alignment with the specific pest management context.

While this study advocates the utility of the Pole-dance bioassay and GP fittings, there are certain limitations when interpreting the findings. The experiments were conducted under stable laboratory conditions, which may not fully represent the variable environmental factors encountered in agricultural fields that can affect volatile persistence and mite behavior. Additionally, *T. urticae* colony used has been maintained in the laboratory for over 10 years without pesticide exposure, thereby responses might differ in field populations with different genetic backgrounds or histories of chemical exposure. Another limitation is that repellency was assessed using only a single dosage for the screening purpose. Furthermore, potential habituation or desensitization to repellents over time was not evaluated beyond the 720-minute observation period (Stockton et al. 2021; Jeon and Tak 2024). Future research should prioritize validating the repellent effects of the most promising compounds identified (e.g., t-cinnamaldehyde, 1,8-cineole, (-)-terpinen-4-ol) under semi-field or field conditions as well as mite strains. Studies investigating the persistence of these specific volatiles on plant surfaces are also crucial for assessing their practical applicability. Additionally, as the volatiles often occurs simultaneously to make mixtures, exploring potential synergistic effects by testing blends of the top-performing volatiles identified in this study could lead to more effective repellent formulations (Masoumi et al. 2016; An and Tak 2022).

Regarding the model parameters, our modeling framework treated replicate-level variation as fixed and pooled all observations per compound. Future work may benefit from incorporating random effects through mixed-effects models to better account for biological and technical variability inherent in behavioral assays. Additionally, while the GP regression used in this study was not constrained to be monotonic, this may lead to minor biologically implausible fluctuations in the fitted curve, despite the cumulative nature of landing behavior. future studies may consider using monotonic GP variants (e.g., via *deepgp* or *rgpstuff* R package) for more realistic enforcement of curve shape. Furthermore, A limitation in the synthetic data generation should be acknowledged. The parameters for the hypothetical compounds were deliberately selected to represent a diverse range of curve shapes rather than being empirically derived, as the 20 tested compounds were insufficient for this purpose. While this approach allowed for model and metrics comparisons across varied hypothetical scenarios, the further generalization and empirical validation of landing curve scenarios would benefit future time-dependent repellency studies.

In conclusion, utilizing the novel Pole-dance bioassay, GP regression proved superior for analyzing complex time-dependent repellency, demonstrating flexibility regardless of the final landing proportion or curve shape. In terms of repellency indices, ET_50_- and AUC-based rankings correlated well, but AUC also provided complementary insights into repellency dynamics above 50% landing and offered quantifiable metrics even when ET_50_ could not be calculated due to strong repellent effects. This study is expected to establish a robust screening system for repellents against spider mites, combining a high-throughput bioassay with statistically rigorous analysis for effective comparison among candidate compounds.

## Supplementary Information

Below is the link to the electronic supplementary material.

Supplementary Material 1

## Data Availability

The raw data is available as supplementary file (data_curated.csv).

## Abbreviations

Median effective time: ET_50_
Area under curve: AUC
Two-parametric Hill equation: Hill2P
Four-parametric Hill equation: Hill4P
Gaussian process: GP
Root Mean Squared Error: RMSE
Mean Absolute Error: MAE

## References

[CR1] Achee NL, Bangs MJ, Farlow R, Killeen GF, Lindsay S, Logan JG, Moore SJ, Rowland M, Sweeney K, Torr SJ, Zwiebel LJ, Grieco JP (2012) Spatial repellents: from discovery and development to evidence-based validation. Malar J 11:164 22583679 10.1186/1475-2875-11-164 PMC3453515

[CR2] Adesanya AW, Lavine MD, Moural TW, Lavine LC, Zhu F, Walsh DB (2021) Mechanisms and management of acaricide resistance for *Tetranychus urticae* in agroecosystems. J Pest Sci 94:639–663

[CR3] An H, Tak J-H (2022) Miticidal and repellent activity of thirty essential oils and their synergistic interaction with vanillin against *Tetranychus urticae* Koch (acari: tetranychidae). Ind Crops Prod 182:114872

[CR4] Antonious GF, Snyder JC (2006) Natural products: repellency and toxicity of wild tomato leaf extracts to the two-spotted spider mite, *Tetranychus urticae* Koch. Journal of Environmental Science and Health, Part B 41:43–55 10.1080/03601230500234893 16393894

[CR5] Badolo A, Ilboudo-Sanogo E, Ouédraogo AP, Costantini C (2004) Evaluation of the sensitivity of *Aedes aegypti* and *Anopheles gambiae* complex mosquitoes to two insect repellents: Deet and Kbr 3023. Trop Med Int Health 9:330–334 14996361 10.1111/j.1365-3156.2004.01206.x

[CR6] da Camara CA, Akhtar Y, Isman MB, Seffrin RC, Born FS (2015) Repellent activity of essential oils from two species of citrus against *Tetranychus urticae* in the laboratory and greenhouse. Crop Prot 74:110–115

[CR7] Dawood MH, Snyder JC (2020) The alcohol and epoxy alcohol of zingiberene, produced in trichomes of wild tomato, are more repellent to spider mites than zingiberene. Front Plant Sci 11:35 32153603 10.3389/fpls.2020.00035 PMC7047219

[CR8] Deletre E, Schatz B, Bourguet D, Chandre F, Williams L, Ratnadass A, Martin T (2016) Prospects for repellent in pest control: current developments and future challenges. Chemoecology 26:127–142

[CR9] Dunn J, Prickett J, Collins D, Macarthur R, Weaver R (2019) Choice test to determine potential attractants and repellents for the sheep scab mite, *Psoroptes ovis* (acari: psoroptidae). Exp Appl Acarol 79:187–194 31598890 10.1007/s10493-019-00416-x

[CR10] Elamir EE, Almadiy AA, Nenaah GE, Alabas AA, Alsaqri HS (2019) Comparing six mathematical link function models of the antifeedant activity of lesser grain borer exposed to sub-lethal concentrations of some extracts from *Calotropis procera*. Bioengineered 10:292–305 31284815 10.1080/21655979.2019.1641399 PMC6650199

[CR11] Faraone N, Evans R, LeBlanc J, Hillier NK (2020) Soil and foliar application of rock dust as natural control agent for two-spotted spider mites on tomato plants. Sci Rep 10:12108 32694587 10.1038/s41598-020-69060-5 PMC7374085

[CR12] Fradin MS, Day JF (2002) Comparative efficacy of insect repellents against mosquito bites. New Engl J Med 347:13–18 12097535 10.1056/NEJMoa011699

[CR13] Ilias A, Vontas J, Tsagkarakou A (2014) Global distribution and origin of target site insecticide resistance mutations in *Tetranychus urticae*. Insect Biochem Mol Biol 48:17–28 24602758 10.1016/j.ibmb.2014.02.006

[CR14] Isman MB (2006) Botanical insecticides, deterrents, and repellents in modern agriculture and an increasingly regulated world. Annu Rev Entomol 51:45–66 16332203 10.1146/annurev.ento.51.110104.151146

[CR15] Isman MB, Miresmailli S (2011) Plant essential oils as repellents and deterrents to agricultural pests recent developments in invertebrate repellents.(pp67–77). ACS

[CR16] Jakubowska M, Dobosz R, Zawada D, Kowalska J (2022) A review of crop protection methods against the twospotted spider mite—*Tetranychus urticae* Koch (acari: tetranychidae)—with special reference to alternative methods. Agriculture (Basel) 12:898

[CR17] Jeon H, Tak J-H (2024) Gustatory habituation to essential oil induces reduced feeding deterrence and neuronal desensitization in Spodoptera Litura. J Pest Sci 98, 321-336

[CR18] Jiang S, Yang L, Bloomquist JR (2019) High-throughput screening method for evaluating spatial repellency and vapour toxicity to mosquitoes. Med Vet Entomol 33:388–396 30907445 10.1111/mve.12377

[CR19] Luker HA (2024) A critical review of current laboratory methods used to evaluate mosquito repellents. Front Insect Sci 4:1320138 38469342 10.3389/finsc.2024.1320138 PMC10926509

[CR20] Manu N, Schilling MW, Phillips TW (2021) Natural and synthetic repellents for pest management of the storage mite *Tyrophagus putrescentiae* (Schrank)(Sarcoptiformes: Acaridae). Insects 12:711 34442277 10.3390/insects12080711 PMC8396925

[CR21] Masoumi F, Youssefi MR, Tabari MA (2016) Combination of carvacrol and thymol against the poultry red mite (*Dermanyssus gallinae*). Parasitol Res 115:4239–4243 27452880 10.1007/s00436-016-5201-4

[CR22] Nerio LS, Olivero-Verbel J, Stashenko E (2010) Repellent activity of essential oils: a review. Bioresour Technol 101:372–378 19729299 10.1016/j.biortech.2009.07.048

[CR23] Oliveira JL, Campos AE, Pereira T, Pasquoto R, Lima R, Grillo DJd, d. Andrade FA, Fraceto (2018) Zein nanoparticles as eco-friendly carrier systems for botanical repellents aiming sustainable agriculture. J Agric Food Chem 66:1330–1340 29345934 10.1021/acs.jafc.7b05552

[CR24] Paluch G, Grodnitzky J, Bartholomay L, Coats J (2009) Quantitative structure-activity relationship of botanical sesquiterpenes: Spatial and contact repellency to the yellow fever mosquito. J Agric Food Chem 57:7618–7625 19645502 10.1021/jf900964e

[CR25] Razuvaeva A, Ulyanova E, Skolotneva E, Andreeva I (2023) Species identification of spider mites (tetranychidae: tetranychinae): a review of methods. Vavilov J Genet Breed 27:240 10.18699/VJGB-23-30 PMC10244583 37293445

[CR26] Roh HS, Park KC, Park CG (2012) Repellent effect of Santalol from sandalwood oil against *Tetranychus urticae* (acari: tetranychidae). J Econ Entomol 105:379–385 22606807 10.1603/ec11262

[CR27] Snyder JC, Antonious GF, Thacker R (2011) A sensitive bioassay for spider mite (*Tetranychus urticae*) repellency: a double bond makes a difference. Exp Appl Acarol 55:215–224 21761225 10.1007/s10493-011-9472-2

[CR28] Stockton DG, Cha DH, Loeb GM (2021) Does habituation affect the efficacy of semiochemical oviposition repellents developed against *Drosophila suzukii*? Environ Entomol 50:1322–1331 34532743 10.1093/ee/nvab099

[CR29] Tak J-H, Isman MB (2017) Acaricidal and repellent activity of plant essential oil-derived terpenes and the effect of binary mixtures against *Tetranychus urticae* Koch (acari: Tetranychidae). Ind Crops Prod 108:786–792

[CR30] Wood MJ, Bull JC, Kanagachandran K, Butt TM (2024) Development and laboratory validation of a plant-derived repellent blend, effective against *Aedes aegypti* [diptera: culicidae], *Anopheles Gambiae* [diptera: culicidae] and *Culex quinquefasciatus* [diptera: culicidae]. PLoS One 19:e0299144 38512948 10.1371/journal.pone.0299144 PMC10956804

[CR31] World Health Organization (2009) Guidelines for efficacy testing of mosquito repellents for human skin Guidelines for efficacy testing of mosquito repellents for human skin

[CR32] Wu W, Yang Y, Feng Y, Ren X, Li Y, Li W, Huang J, Kong L, Chen X, Lin Z (2022) Study of the repellent activity of 60 essential oils and their main constituents against *Aedes albopictus*, and nano-formulation development. Insects 13:1077 36554987 10.3390/insects13121077 PMC9782114

[CR33] Yoon J, Tak J-H (2018) Toxicity and repellent activity of plant essential oils and their blending effects against two spotted spider mites, *Tetranychus urticae* Koch. Korean J Appl Entomol 57:199–207

