# Supplementary material for: Comparison of statistical models for time-dependent repellency using the novel Pole-dance bioassay against Tetranychus urticae Koch
Source: Exp Appl Acarol. 2025 Aug 21;95(3):34. doi: 10.1007/s10493-025-01058-y (PMC12370553; doi:10.1007/s10493-025-01058-y)
Supplement: Supplementary file 1 — Supplementary Material 1 [file 10493_2025_1058_MOESM1_ESM.docx]

Supplementary Information for:

Comparison of Statistical Models for Time-Dependent Repellency Using the Novel Pole-Dance Bioassay against Tetranychus urticae Koch

Junho Yoon

Research Institute of Agricultural and Life Sciences, Seoul National University, Seoul, 08826, South Korea

**
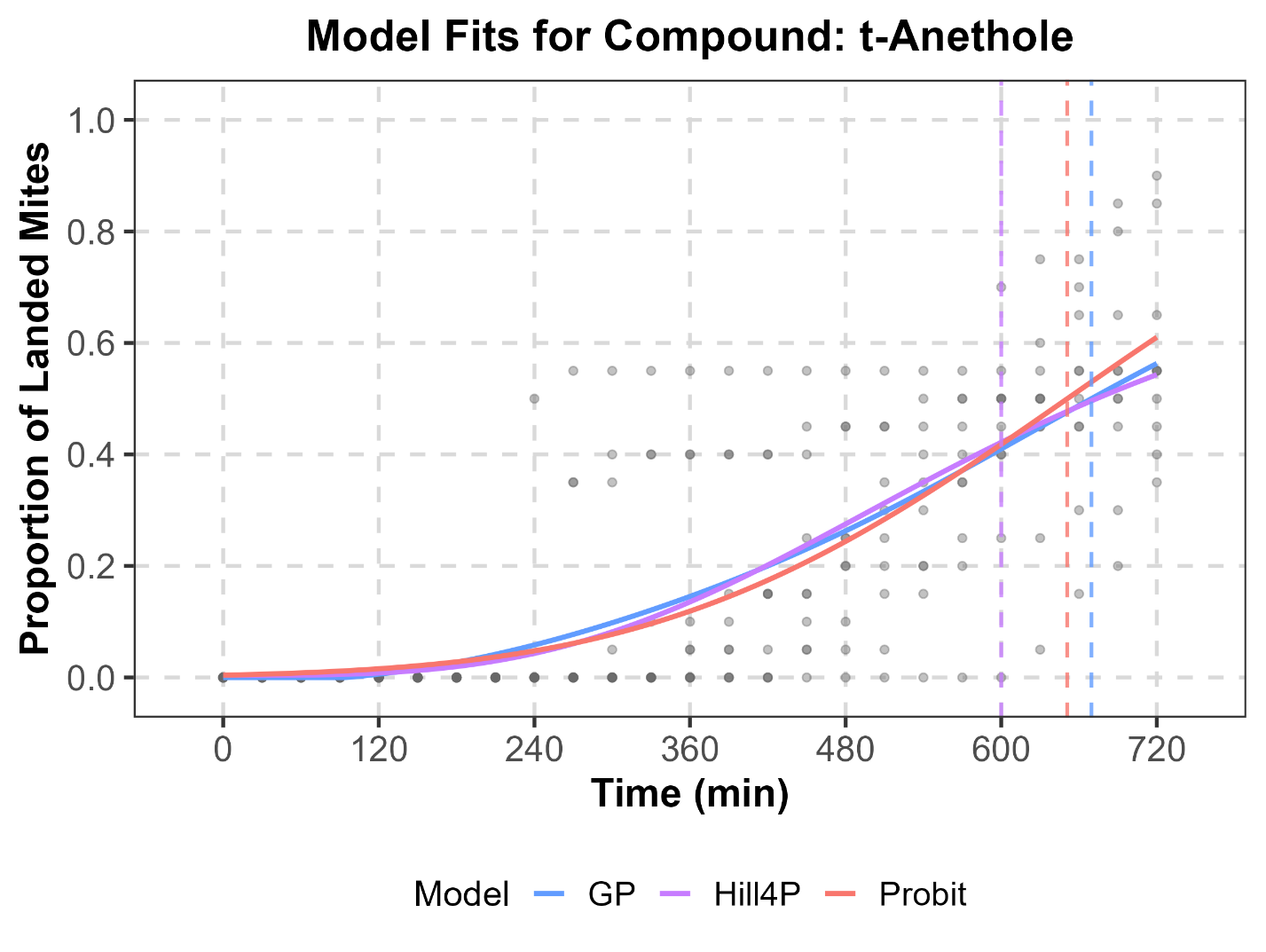
**

**Supplementary Fig. S1.** Comparison of observed time-dependent landing data for *T. urticae* exposed to *t*-Anethole (points) with fitted trajectories from Probit, Hill2P (not converged), Hill4P, and GP models. Dashed vertical lines indicate ET_50_ estimates from each model, where available.


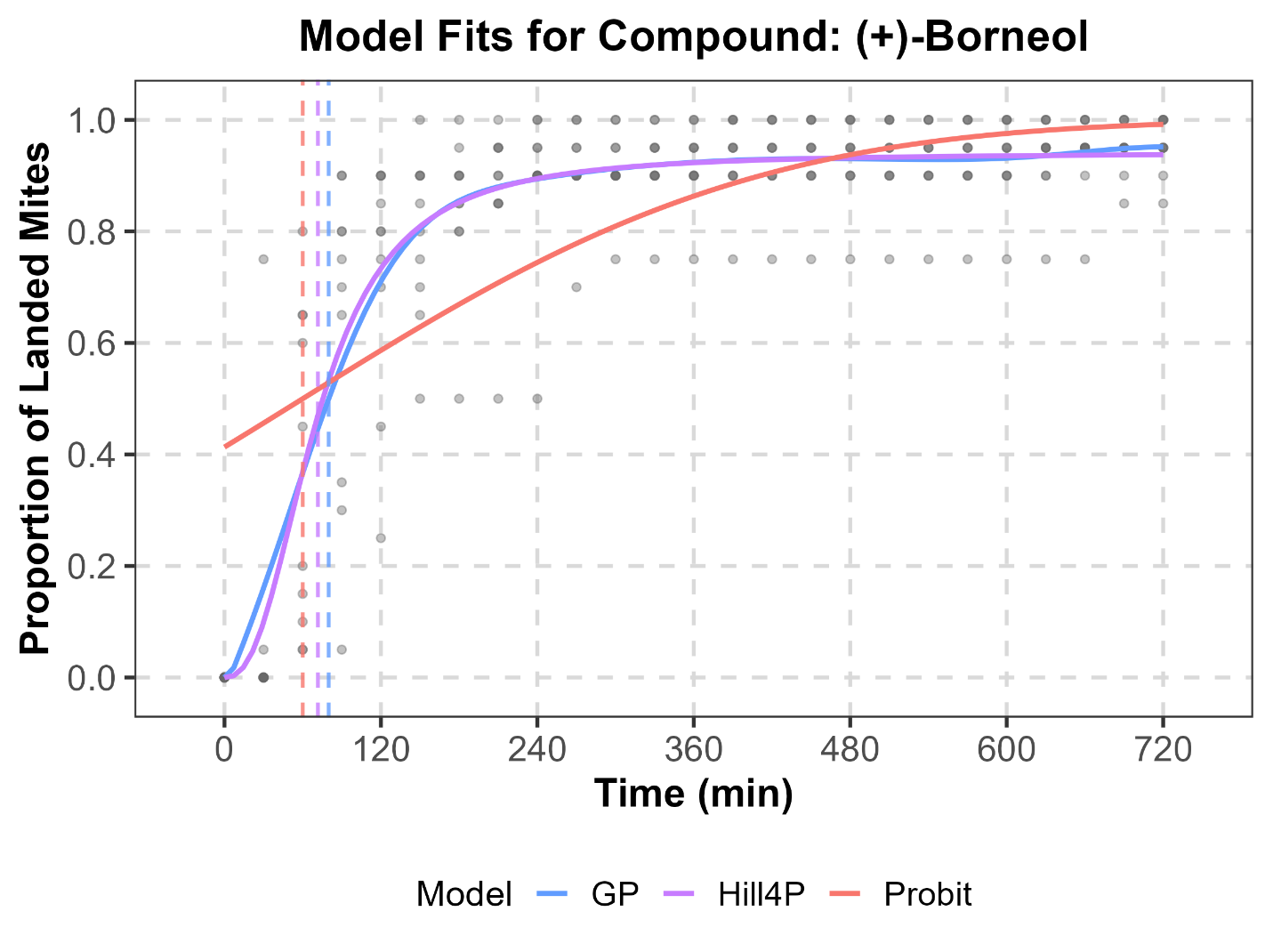


**Supplementary Fig. S2.** Comparison of observed time-dependent landing data for *T. urticae* exposed to (+)-borneol (points) with fitted trajectories from Probit, Hill2P (not converged), Hill4P, and GP models. Dashed vertical lines indicate ET_50_ estimates from each model, where available.


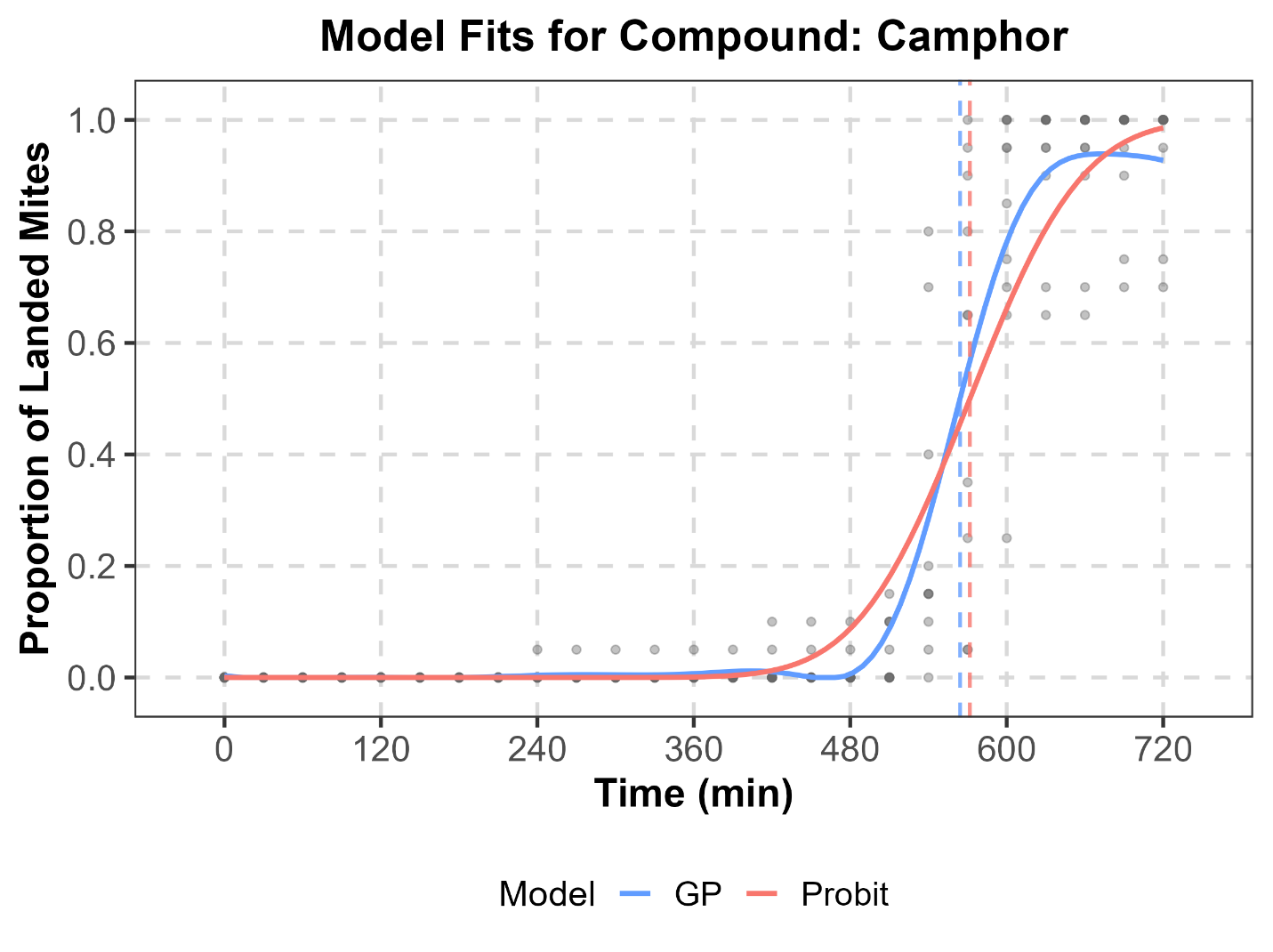


**Supplementary Fig. S3.** Comparison of observed time-dependent landing data for *T. urticae* exposed to camphor (points) with fitted trajectories from Probit, Hill2P (not converged), Hill4P (not converged), and GP models. Dashed vertical lines indicate ET_50_ estimates from each model, where available.


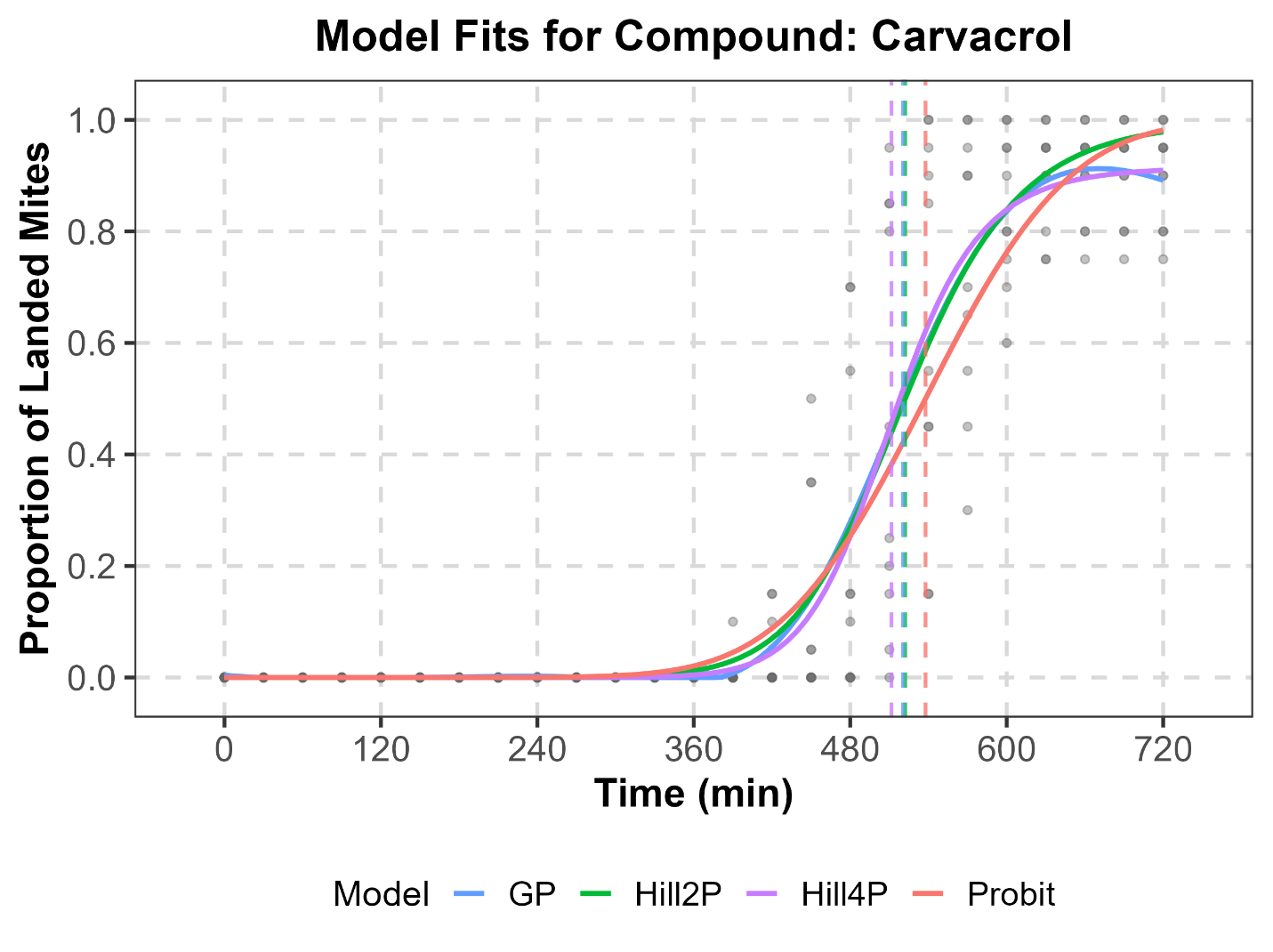


**Supplementary Fig. S4.** Comparison of observed time-dependent landing data for *T. urticae* exposed to camphor (points) with fitted trajectories from Probit, Hill2P, Hill4P, and GP models. Dashed vertical lines indicate ET_50_ estimates from each model, where available.


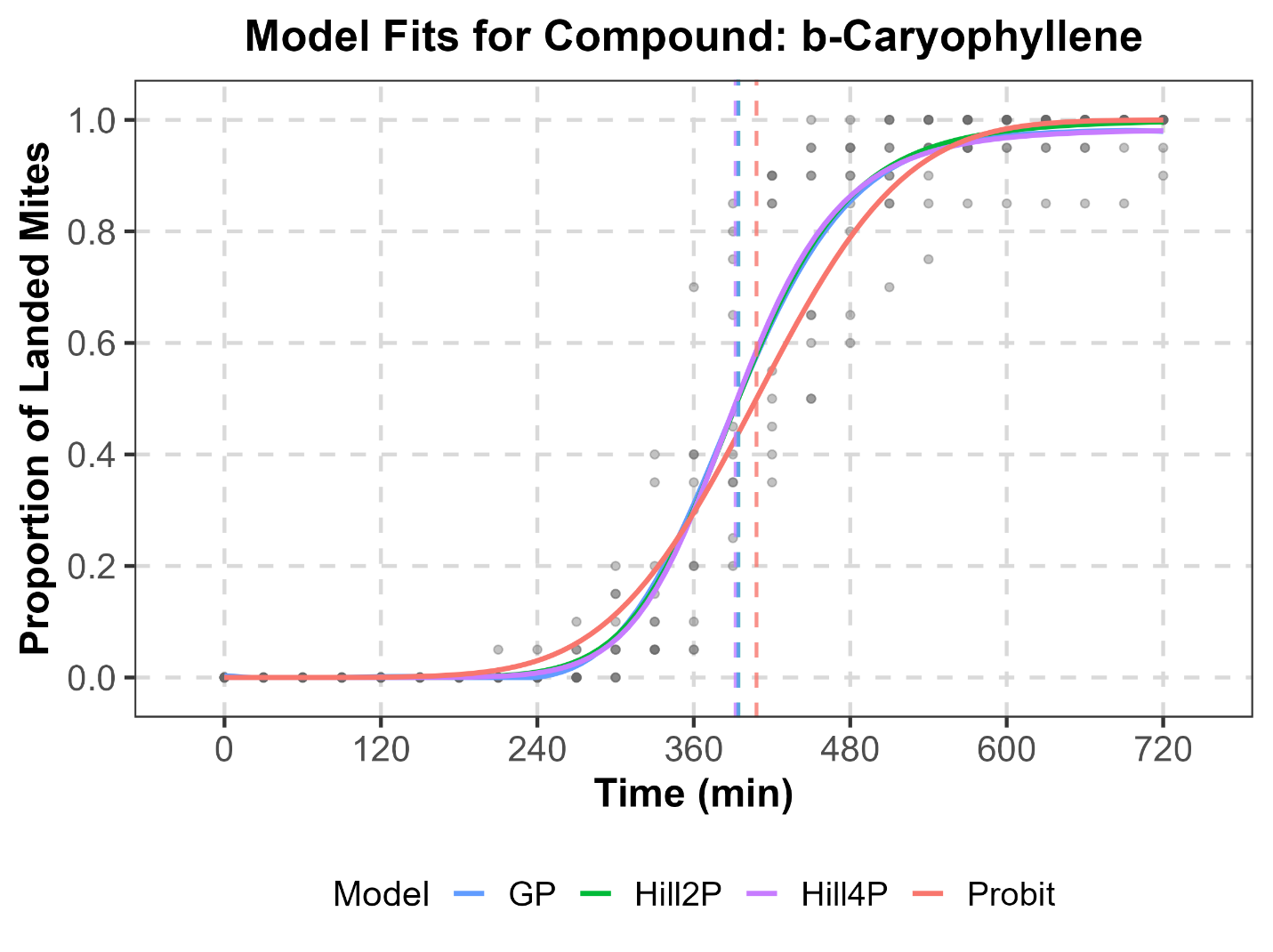


**Supplementary Fig. S5.** Comparison of observed time-dependent landing data for *T. urticae* exposed to β-caryophyllene (points) with fitted trajectories from Probit, Hill2P, Hill4P, and GP models. Dashed vertical lines indicate ET_50_ estimates from each model, where available.


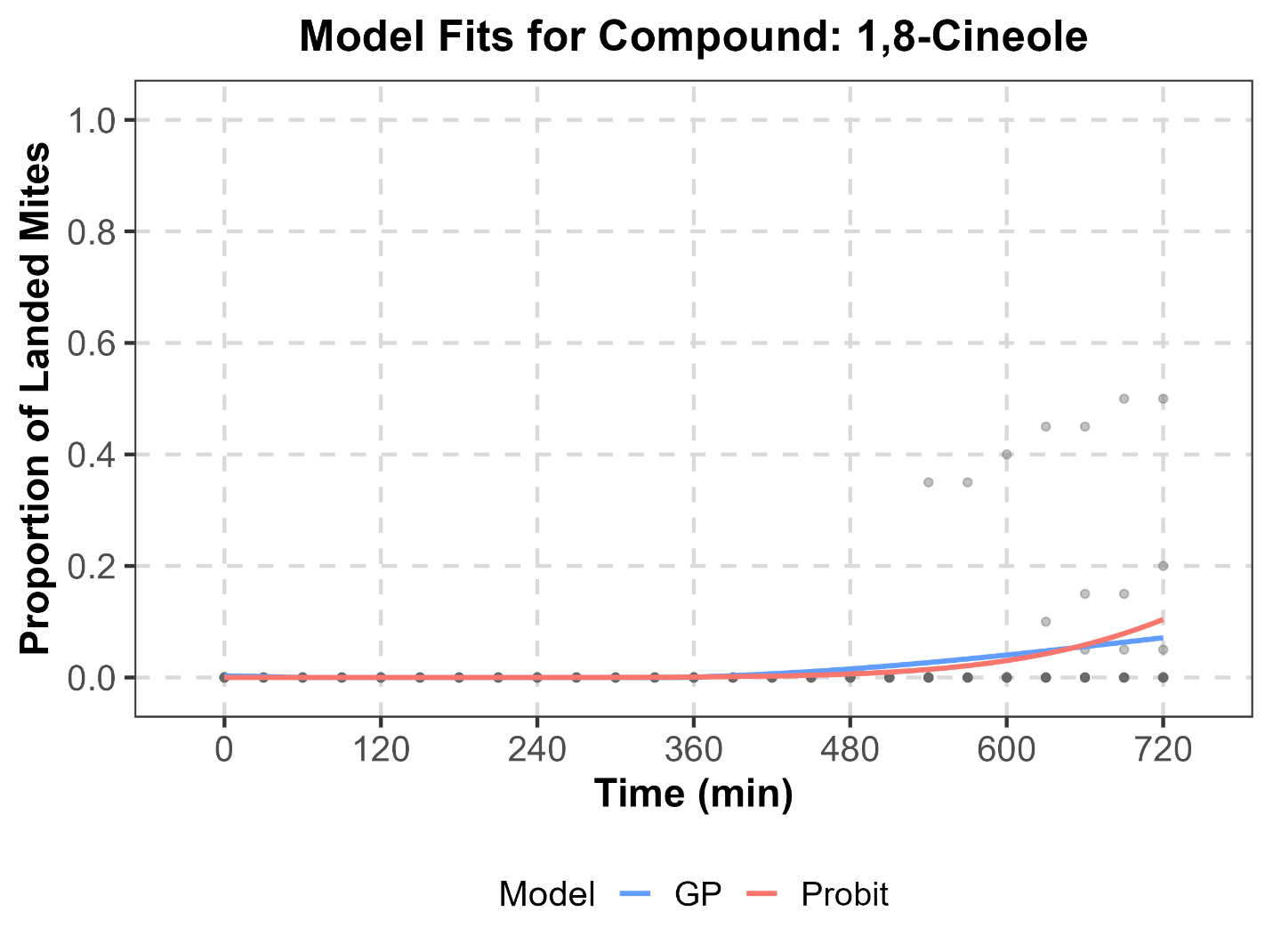


**Supplementary Fig. S6.** Comparison of observed time-dependent landing data for *T. urticae* exposed to 1,8-cineole (points) with fitted trajectories from Probit, Hill2P (not converged), Hill4P (not converged), and GP models. Dashed vertical lines indicate ET_50_ estimates from each model, where available.


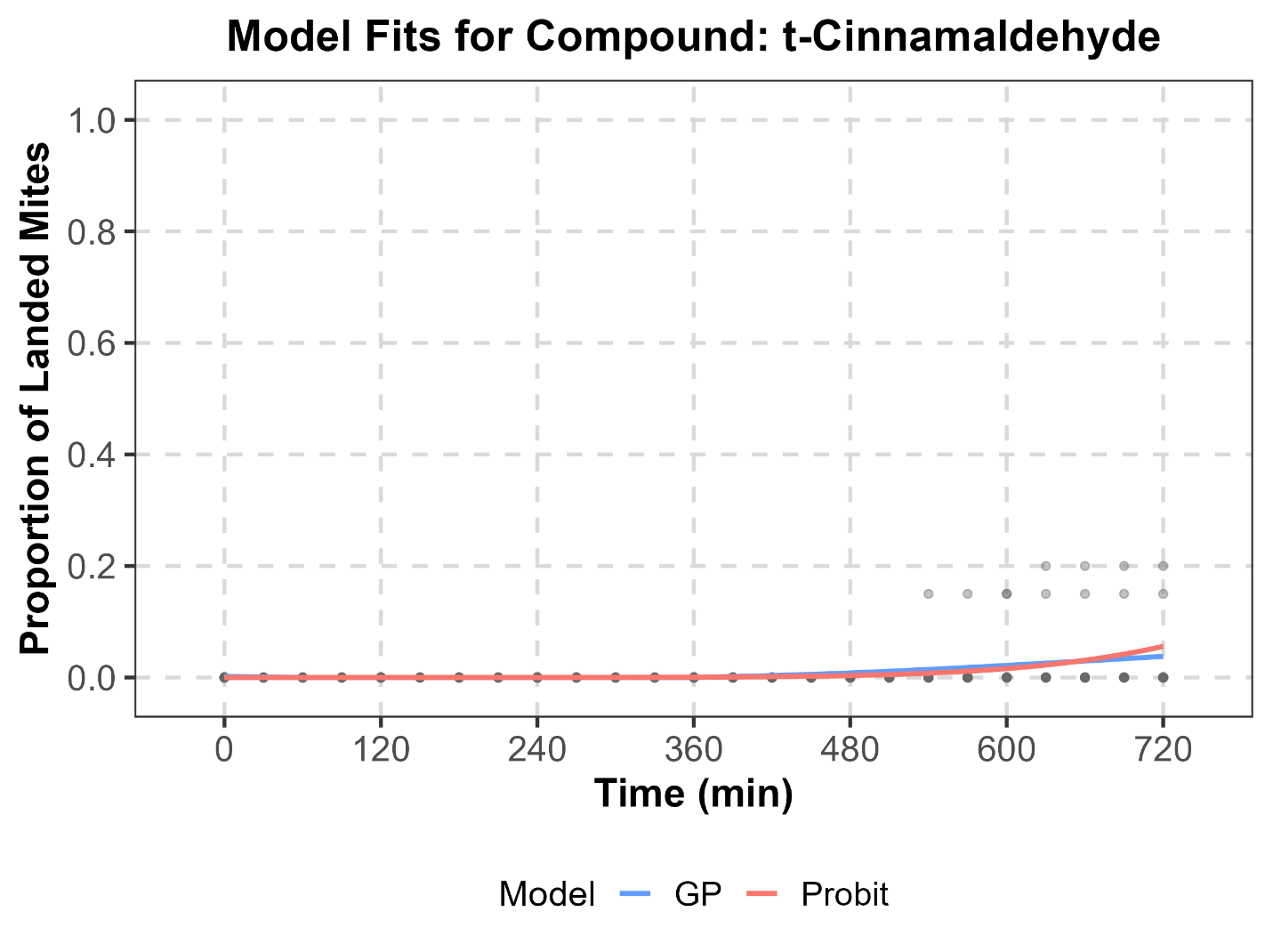


**Supplementary Fig. S7.** Comparison of observed time-dependent landing data for *T. urticae* exposed to *t*-cinnamaldehyde (points) with fitted trajectories from Probit, Hill2P (not converged), Hill4P (not converged), and GP models. Dashed vertical lines indicate ET_50_ estimates from each model, where available.


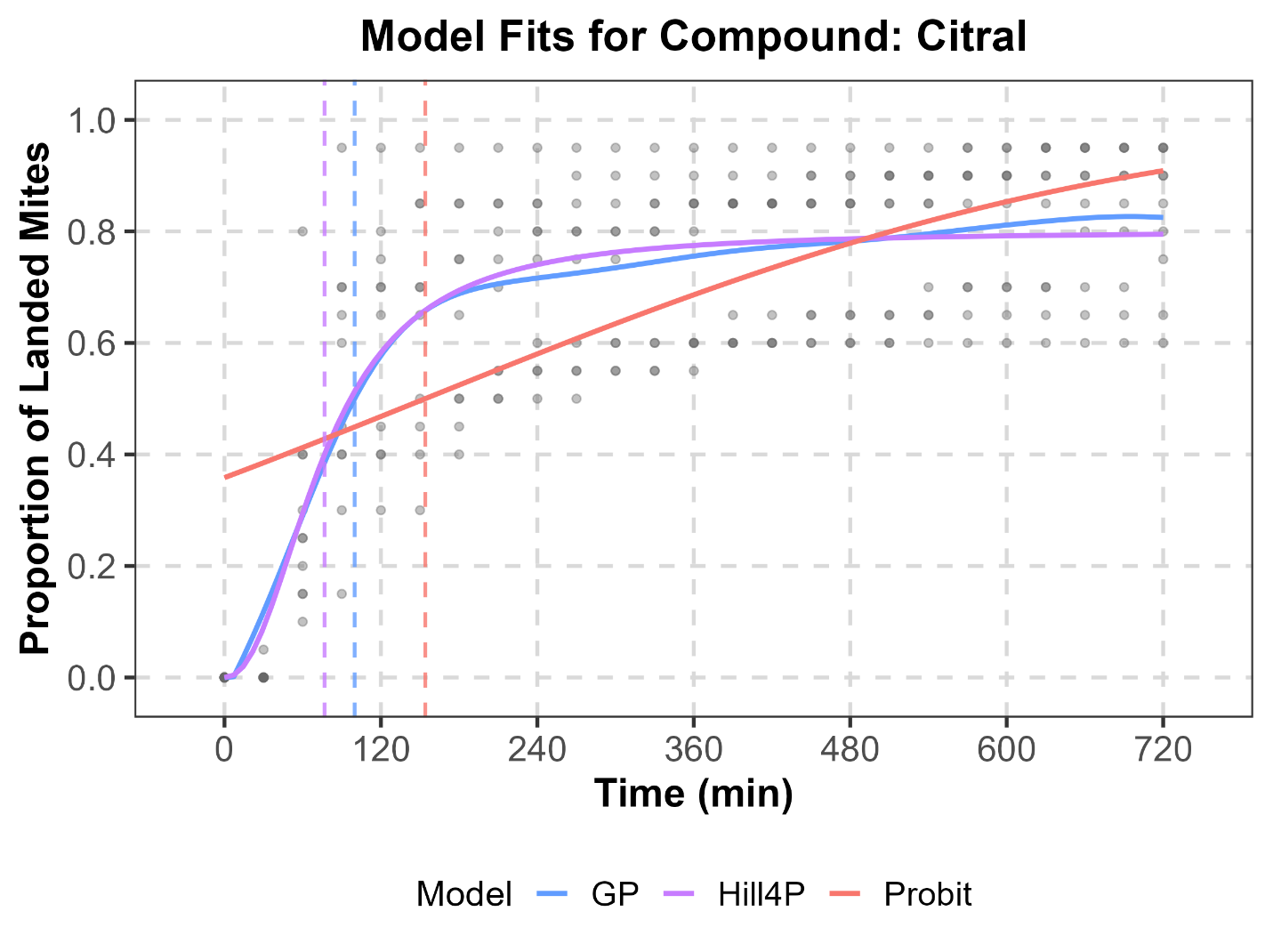
 **Supplementary Fig. S8.** Comparison of observed time-dependent landing data for *T. urticae* exposed to citral (points) with fitted trajectories from Probit, Hill2P, Hill4P (not converged), and GP models. Dashed vertical lines indicate ET_50_ estimates from each model, where available.


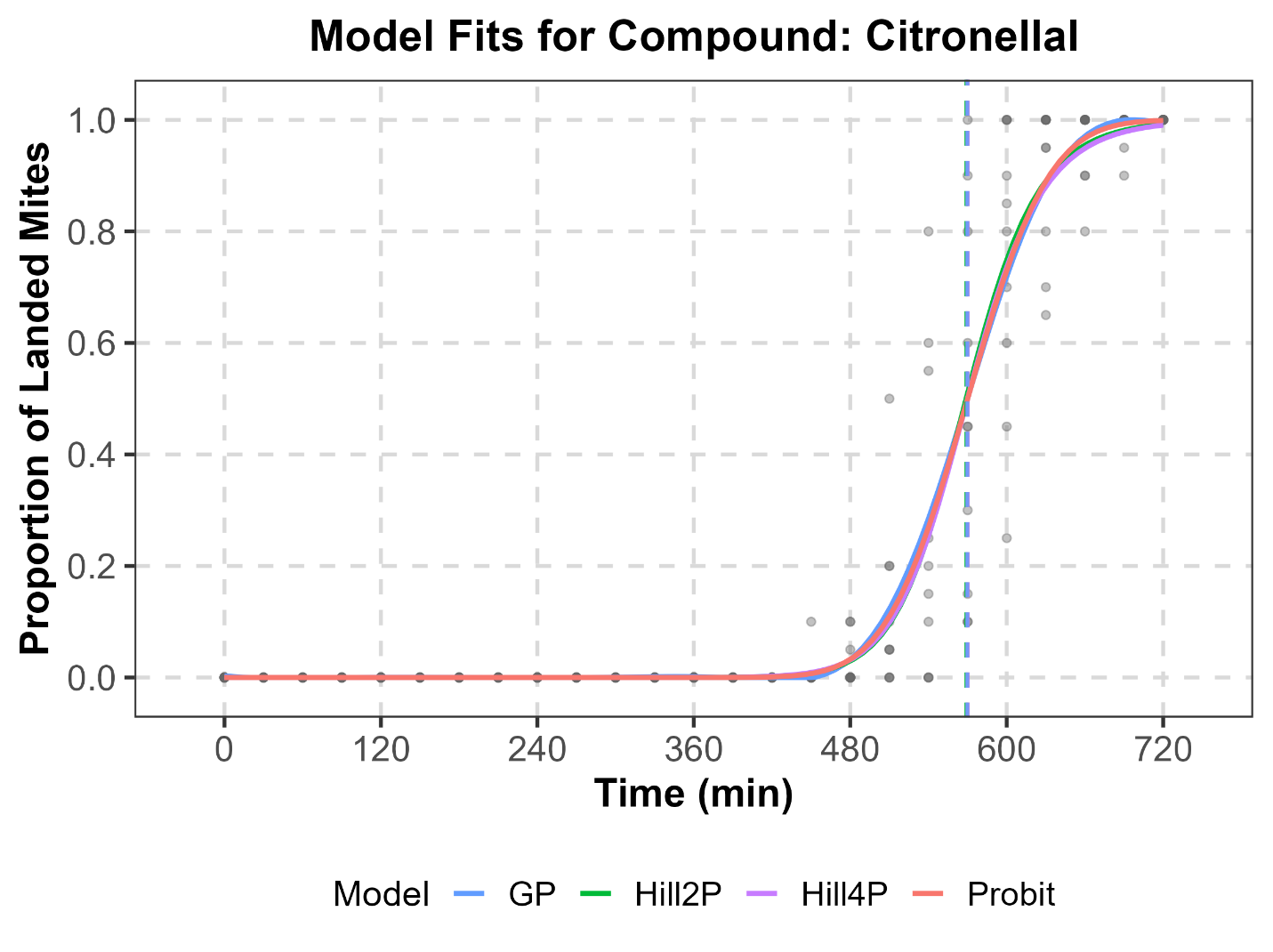


**Supplementary Fig. S9.** Comparison of observed time-dependent landing data for *T. urticae* exposed to citronellal (points) with fitted trajectories from Probit, Hill2P, Hill4P, and GP models. Dashed vertical lines indicate ET_50_ estimates from each model, where available.


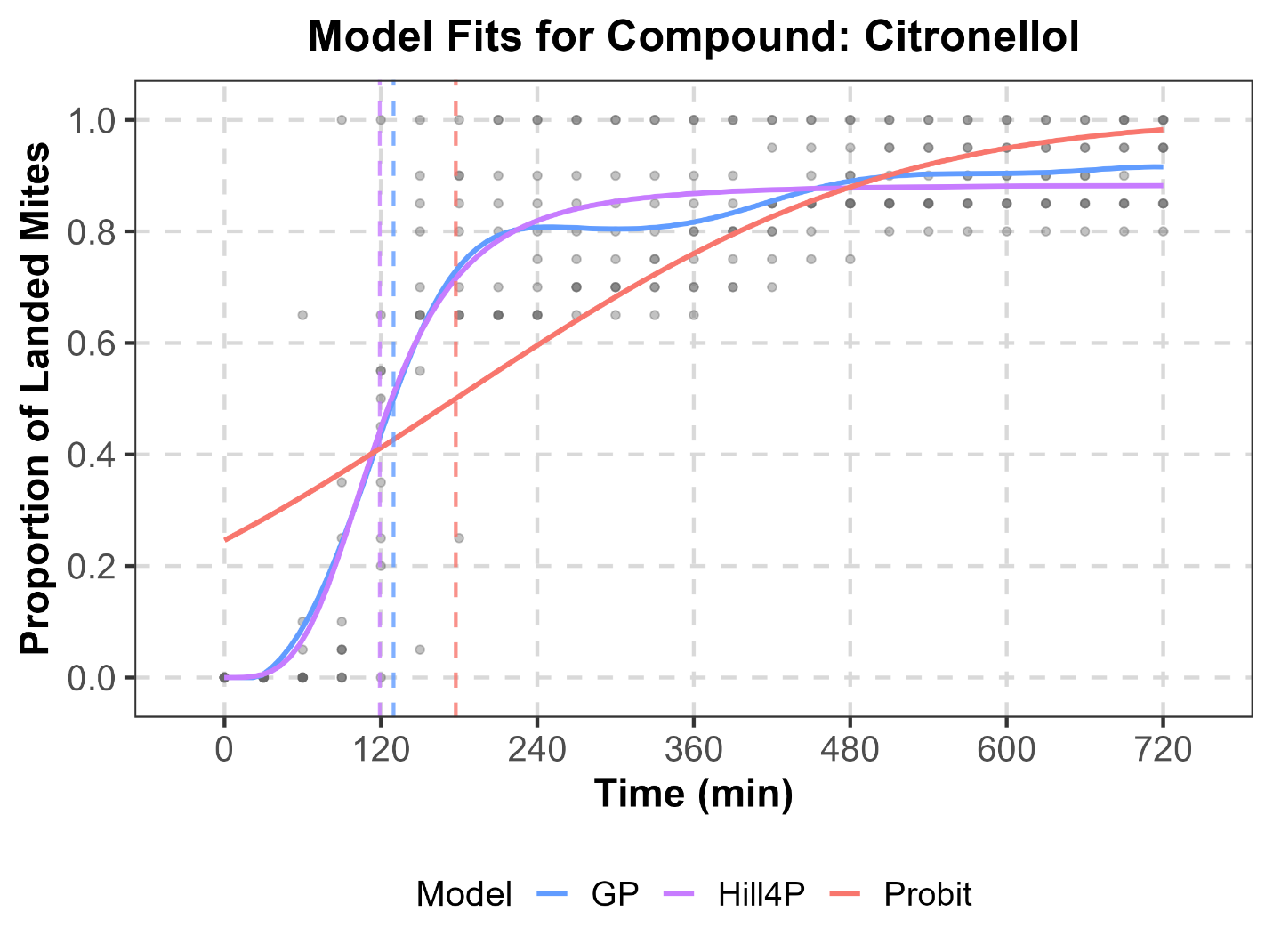


**Supplementary Fig. S10.** Comparison of observed time-dependent landing data for *T. urticae* exposed to citronellal (points) with fitted trajectories from Probit, Hill2P (not converged), Hill4P, and GP models. Dashed vertical lines indicate ET_50_ estimates from each model, where available.


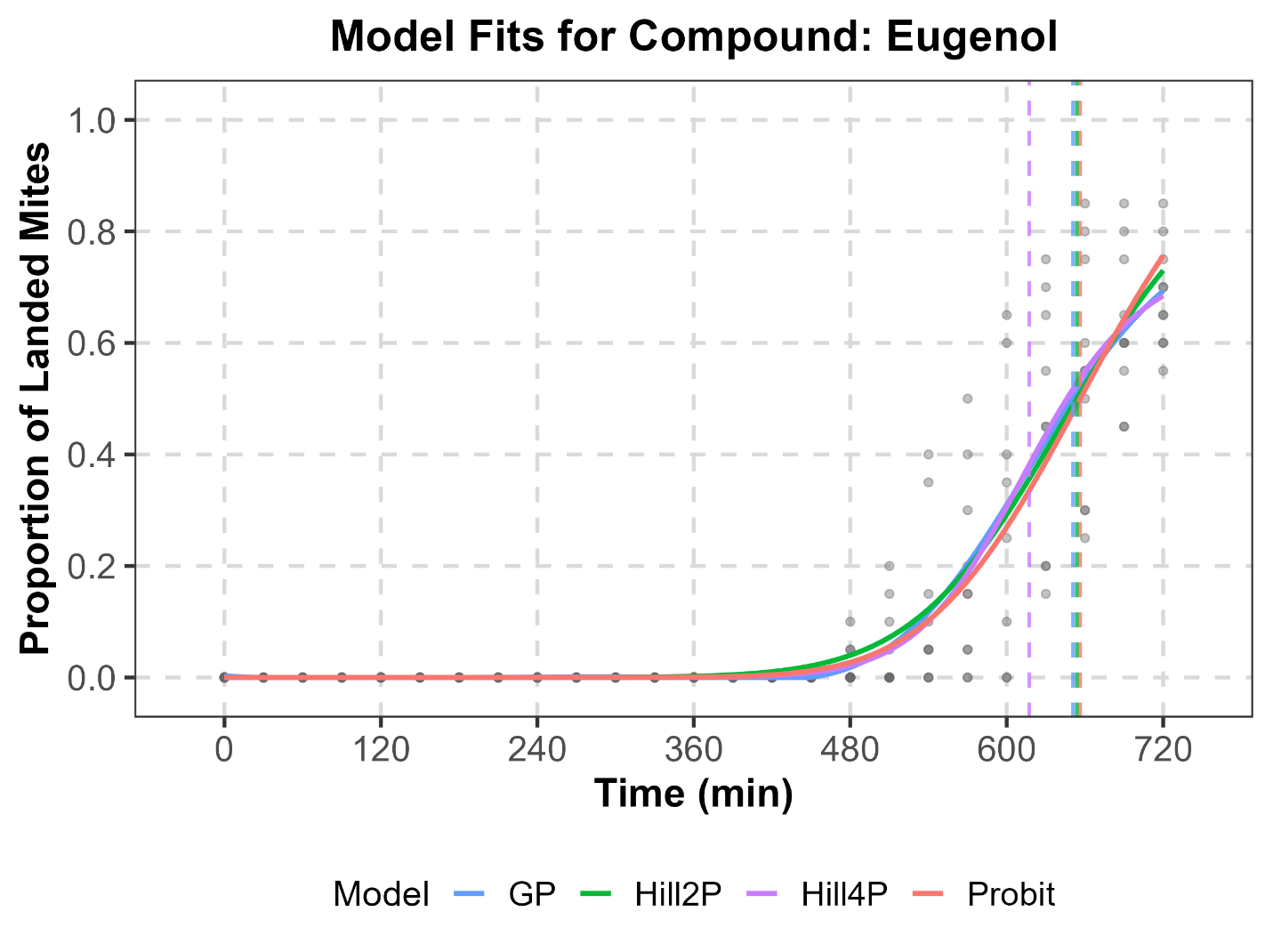


**Supplementary Fig. S11.** Comparison of observed time-dependent landing data for *T. urticae* exposed to eugenol (points) with fitted trajectories from Probit, Hill2P, Hill4P, and GP models. Dashed vertical lines indicate ET_50_ estimates from each model, where available.


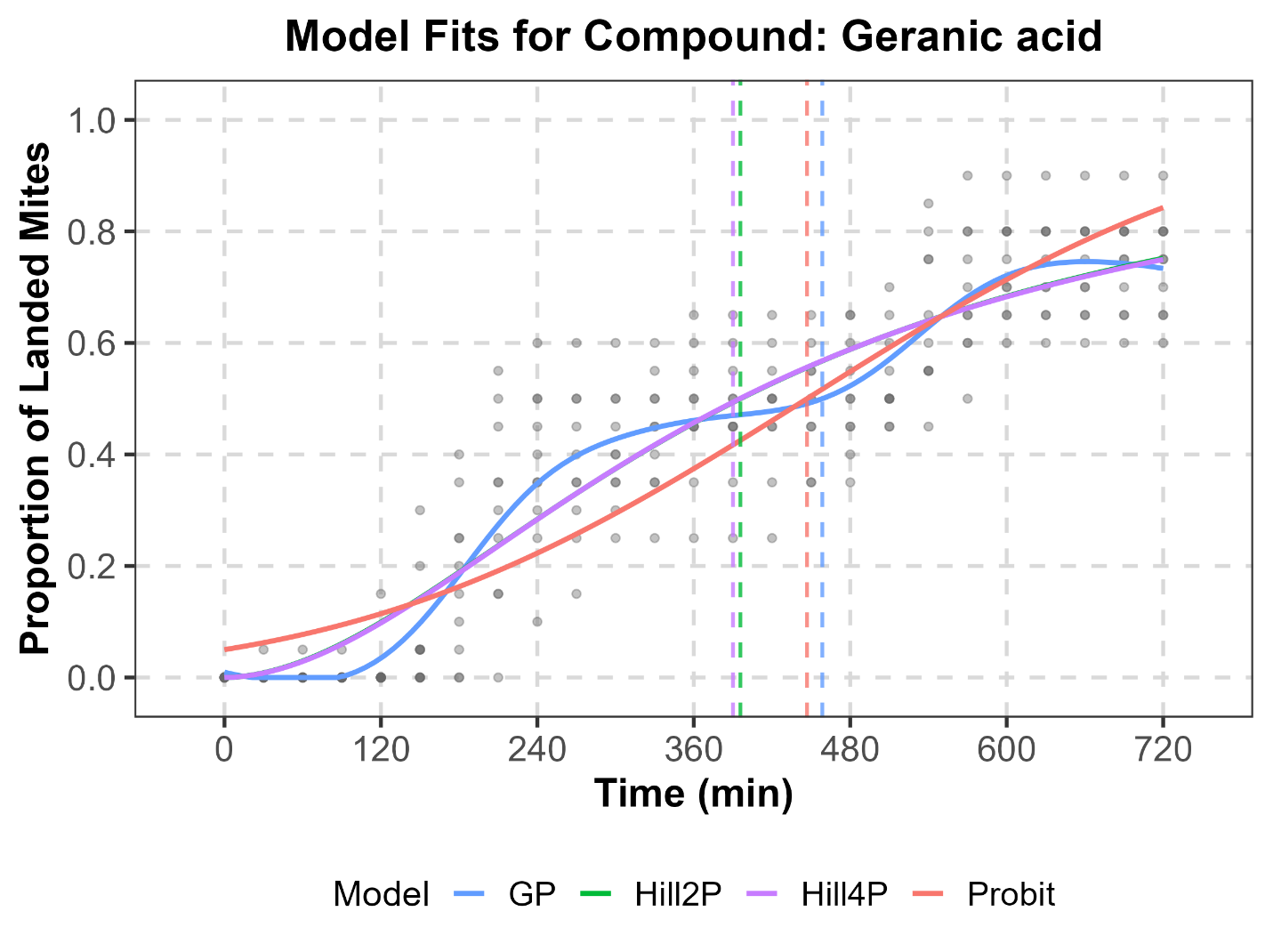


**Supplementary Fig. S12.** Comparison of observed time-dependent landing data for *T. urticae* exposed to geranic acid (points) with fitted trajectories from Probit, Hill2P, Hill4P, and GP models. Dashed vertical lines indicate ET_50_ estimates from each model, where available.


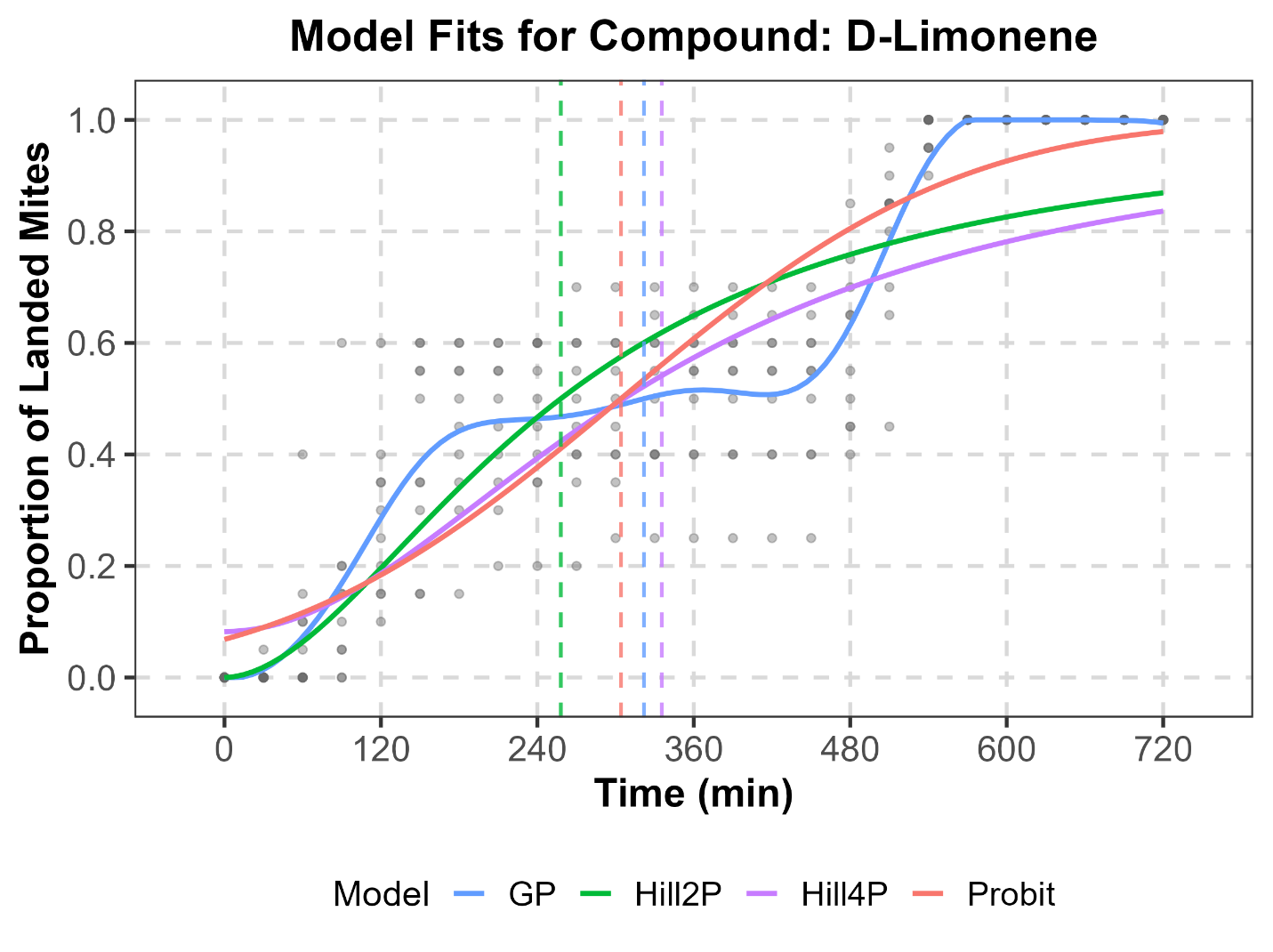


**Supplementary Fig. S13.** Comparison of observed time-dependent landing data for *T. urticae* exposed to D-limonene (points) with fitted trajectories from Probit, Hill2P, Hill4P, and GP models. Dashed vertical lines indicate ET_50_ estimates from each model, where available.


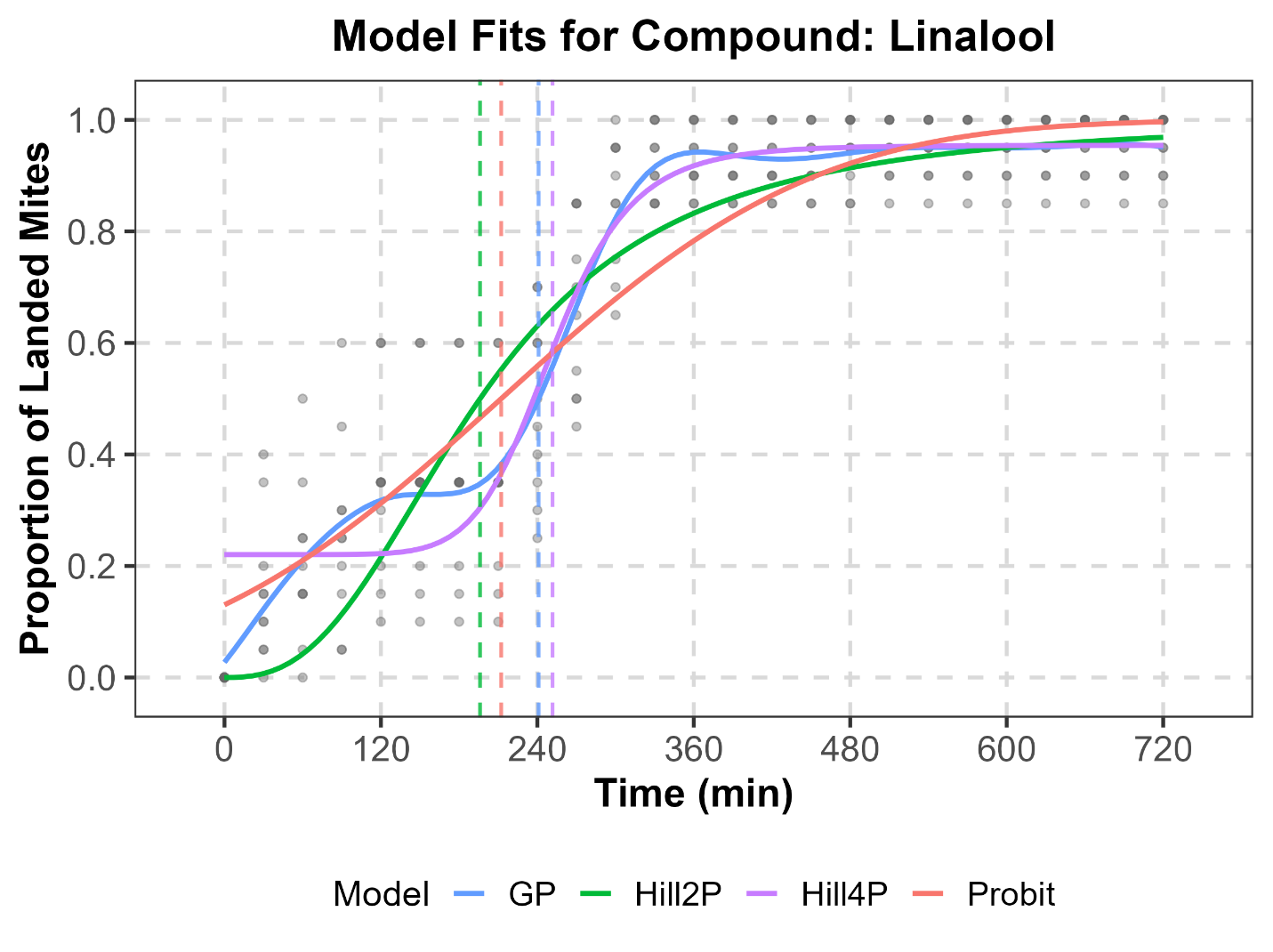


**Supplementary Fig. S14.** Comparison of observed time-dependent landing data for *T. urticae* exposed to linalool (points) with fitted trajectories from Probit, Hill2P, Hill4P, and GP models. Dashed vertical lines indicate ET_50_ estimates from each model, where available.


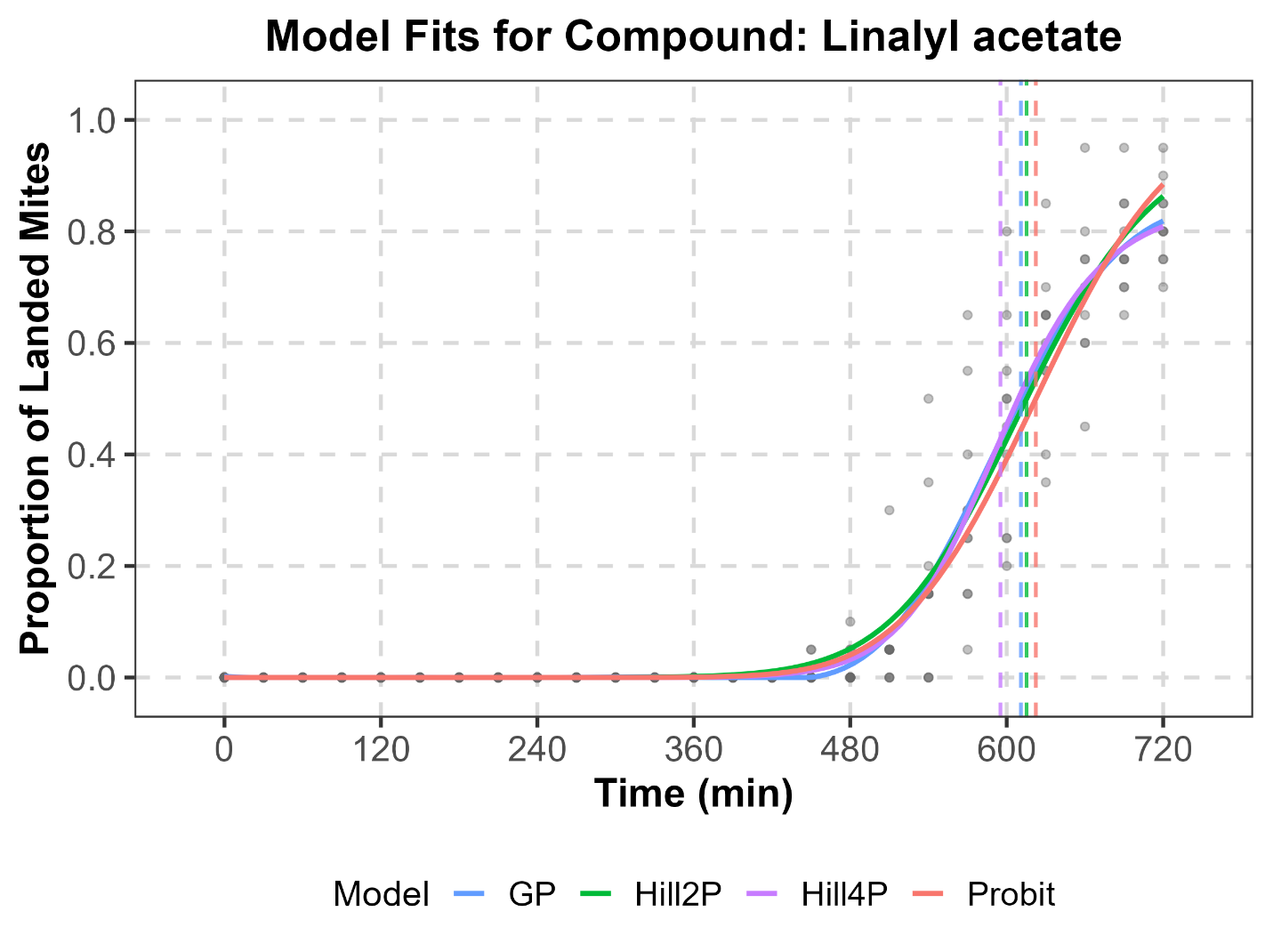


**Supplementary Fig. S15.** Comparison of observed time-dependent landing data for *T. urticae* exposed to linalyl acetate (points) with fitted trajectories from Probit, Hill2P, Hill4P, and GP models. Dashed vertical lines indicate ET_50_ estimates from each model, where available.


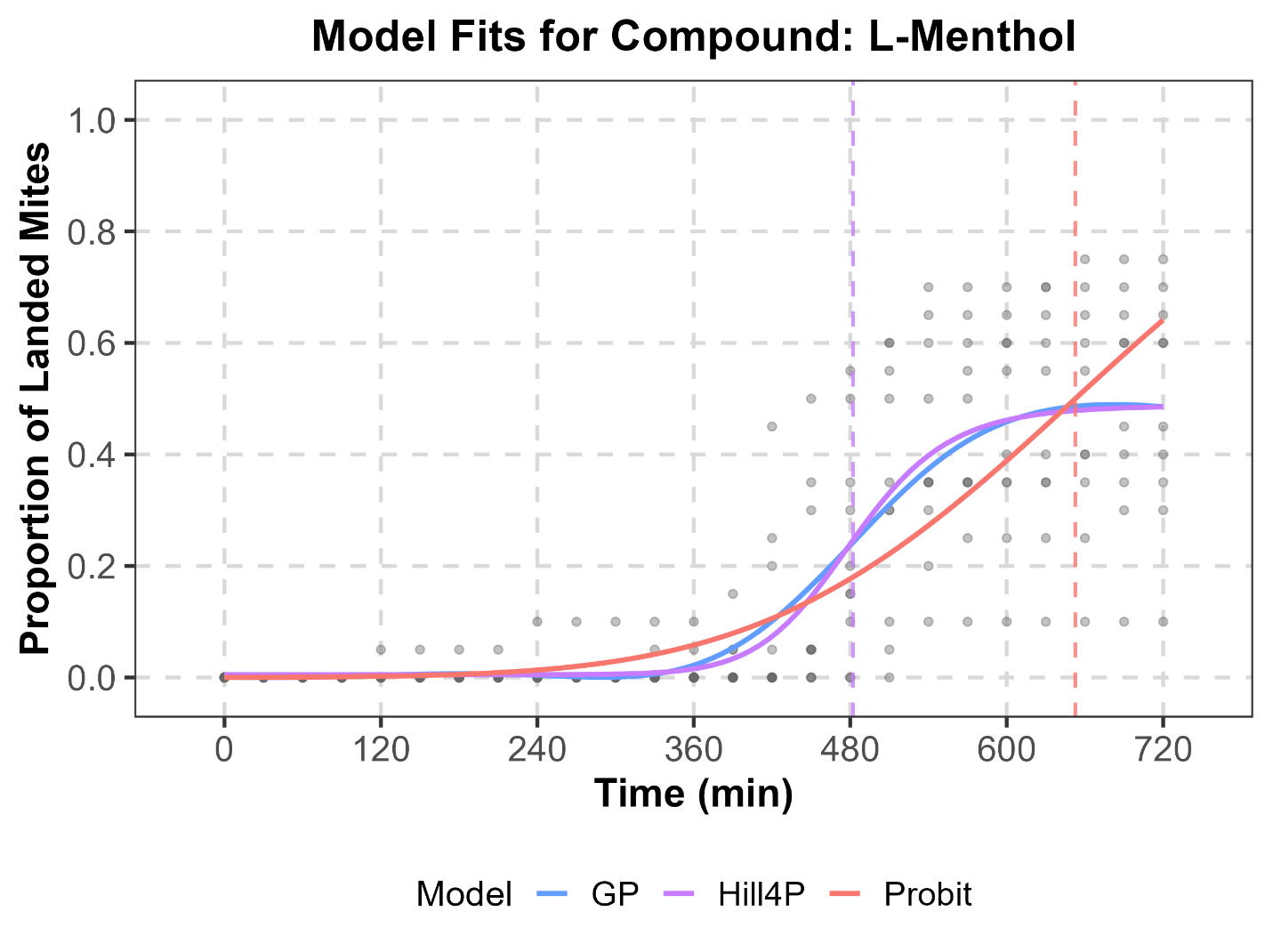


**Supplementary Fig. S16.** Comparison of observed time-dependent landing data for *T. urticae* exposed to L-Menthol (points) with fitted trajectories from Probit, Hill2P (not converged), Hill4P, and GP models. Dashed vertical lines indicate ET_50_ estimates from each model, where available.


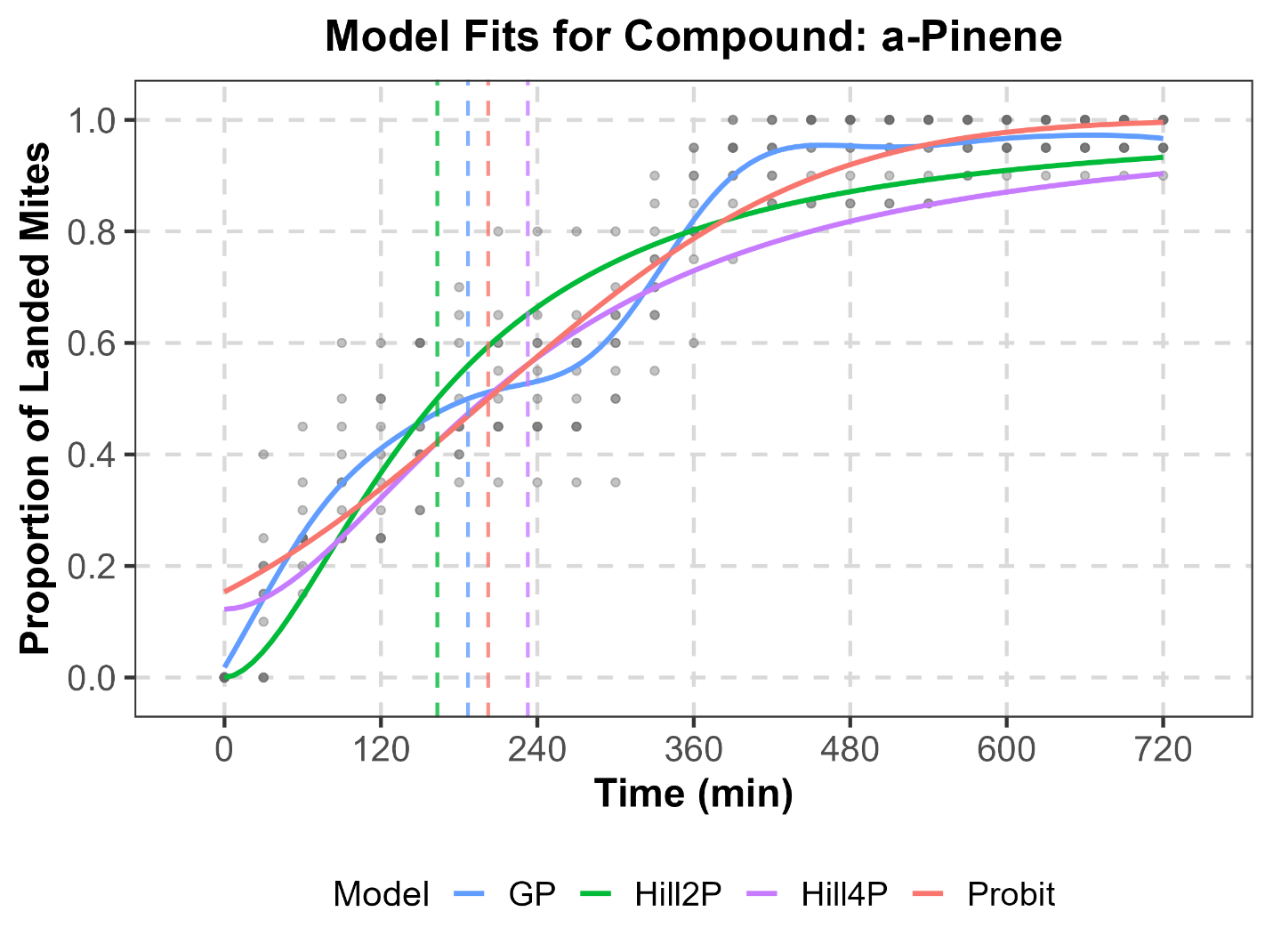


**Supplementary Fig. S17.** Comparison of observed time-dependent landing data for *T. urticae* exposed to α-pinene (points) with fitted trajectories from Probit, Hill2P, Hill4P, and GP models. Dashed vertical lines indicate ET_50_ estimates from each model, where available.


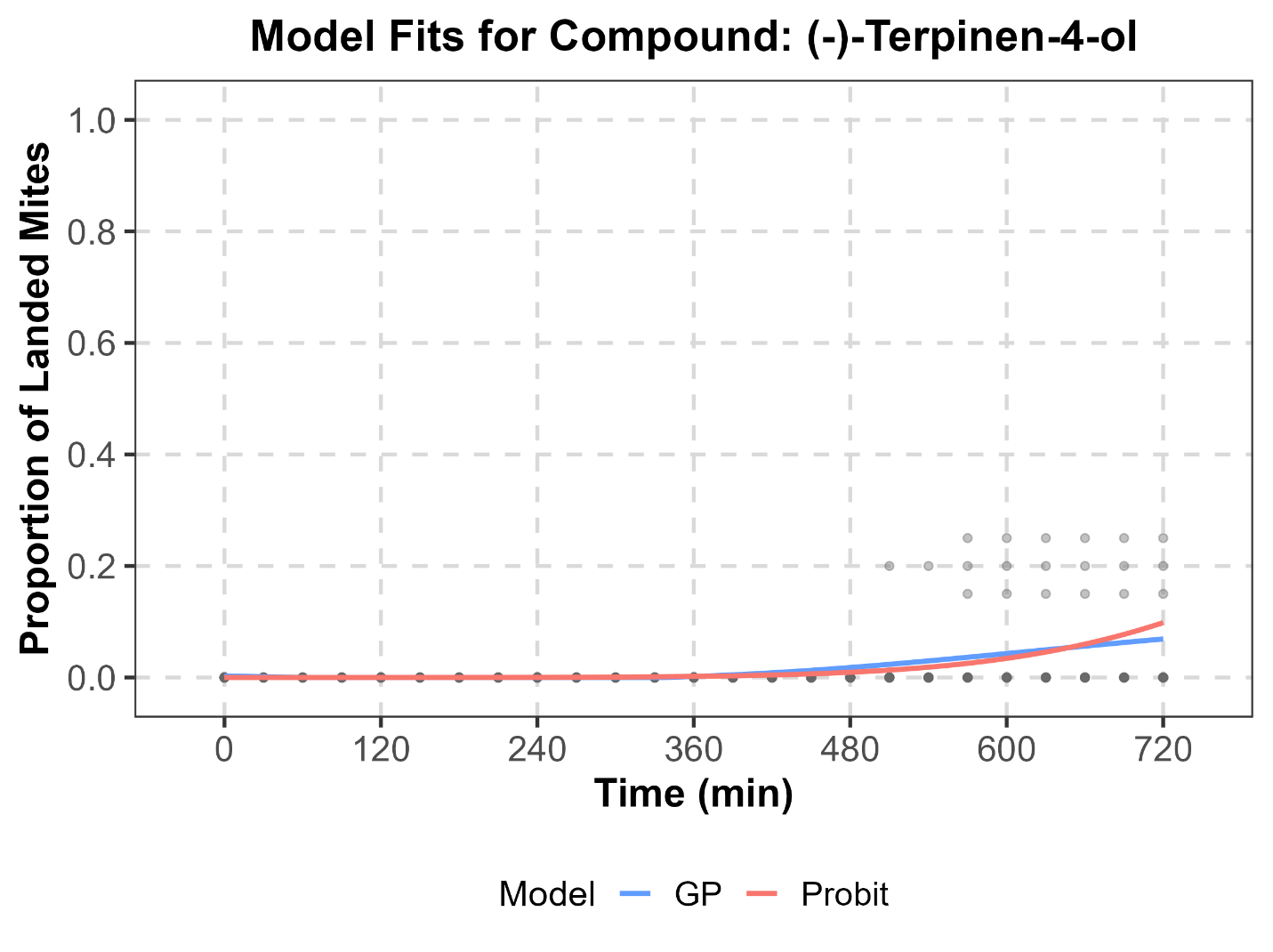


**Supplementary Fig. S18.** Comparison of observed time-dependent landing data for *T. urticae* exposed to (-)-terpinen-4-ol (points) with fitted trajectories from Probit, Hill2P, Hill4P, and GP models. Dashed vertical lines indicate ET_50_ estimates from each model, where available.


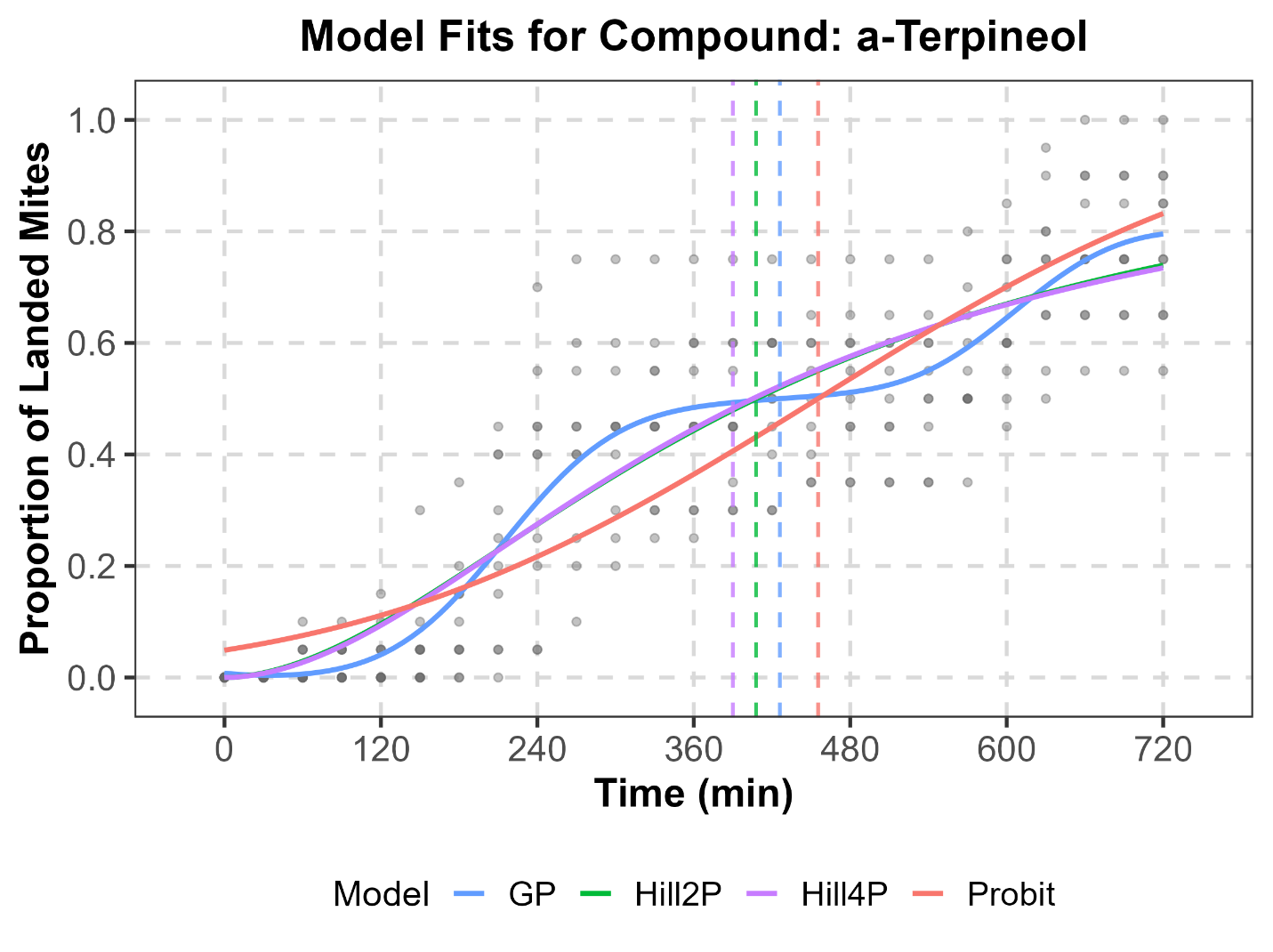


**Supplementary Fig. S19.** Comparison of observed time-dependent landing data for *T. urticae* exposed to α-terpineol (points) with fitted trajectories from Probit, Hill2P, Hill4P, and GP models. Dashed vertical lines indicate ET_50_ estimates from each model, where available.


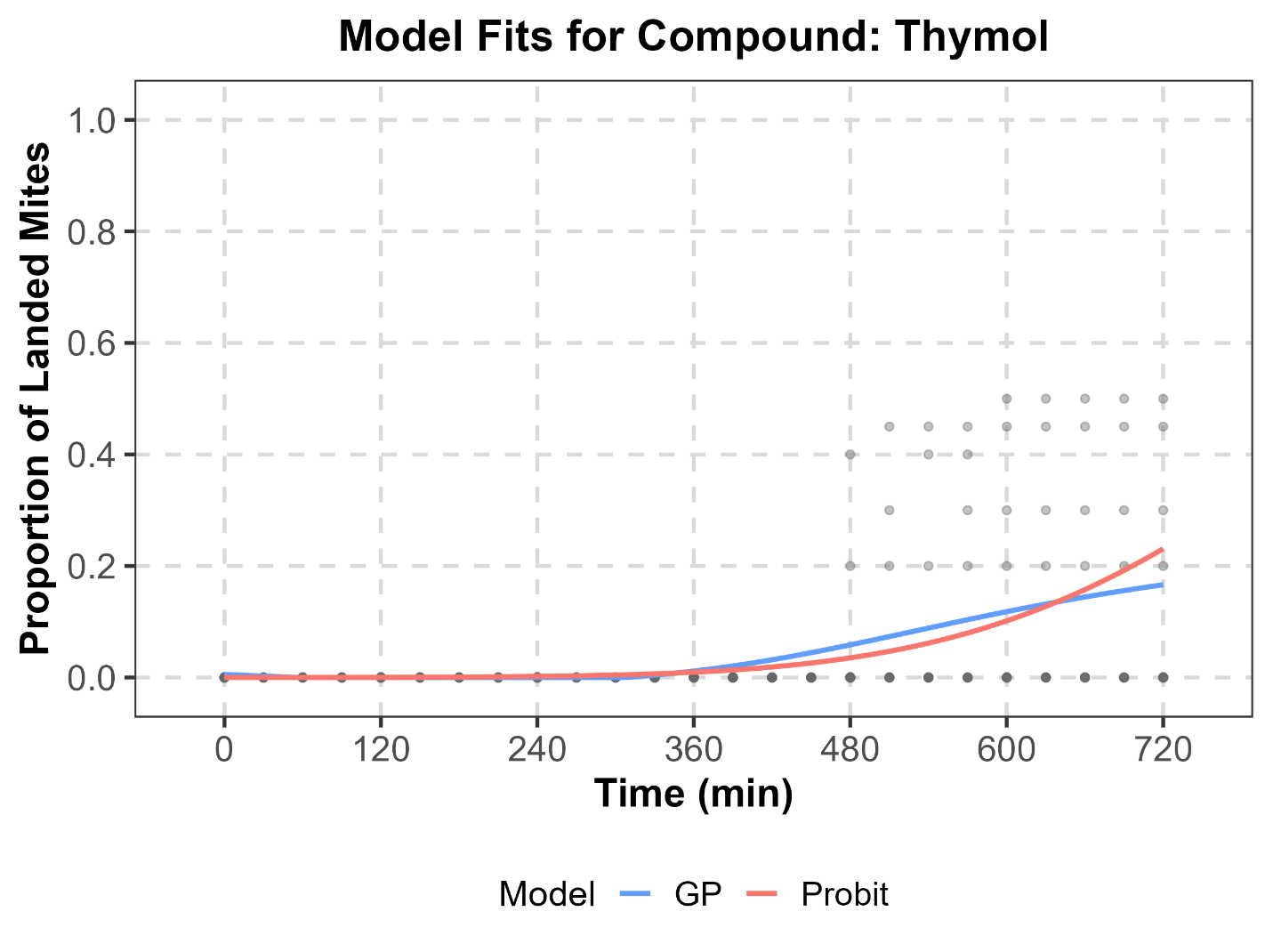


**Supplementary Fig. S20.** Comparison of observed time-dependent landing data for *T. urticae* exposed to thymol (points) with fitted trajectories from Probit, Hill2P (not converged), Hill4P (not converged), and GP models. Dashed vertical lines indicate ET_50_ estimates from each model, where available.


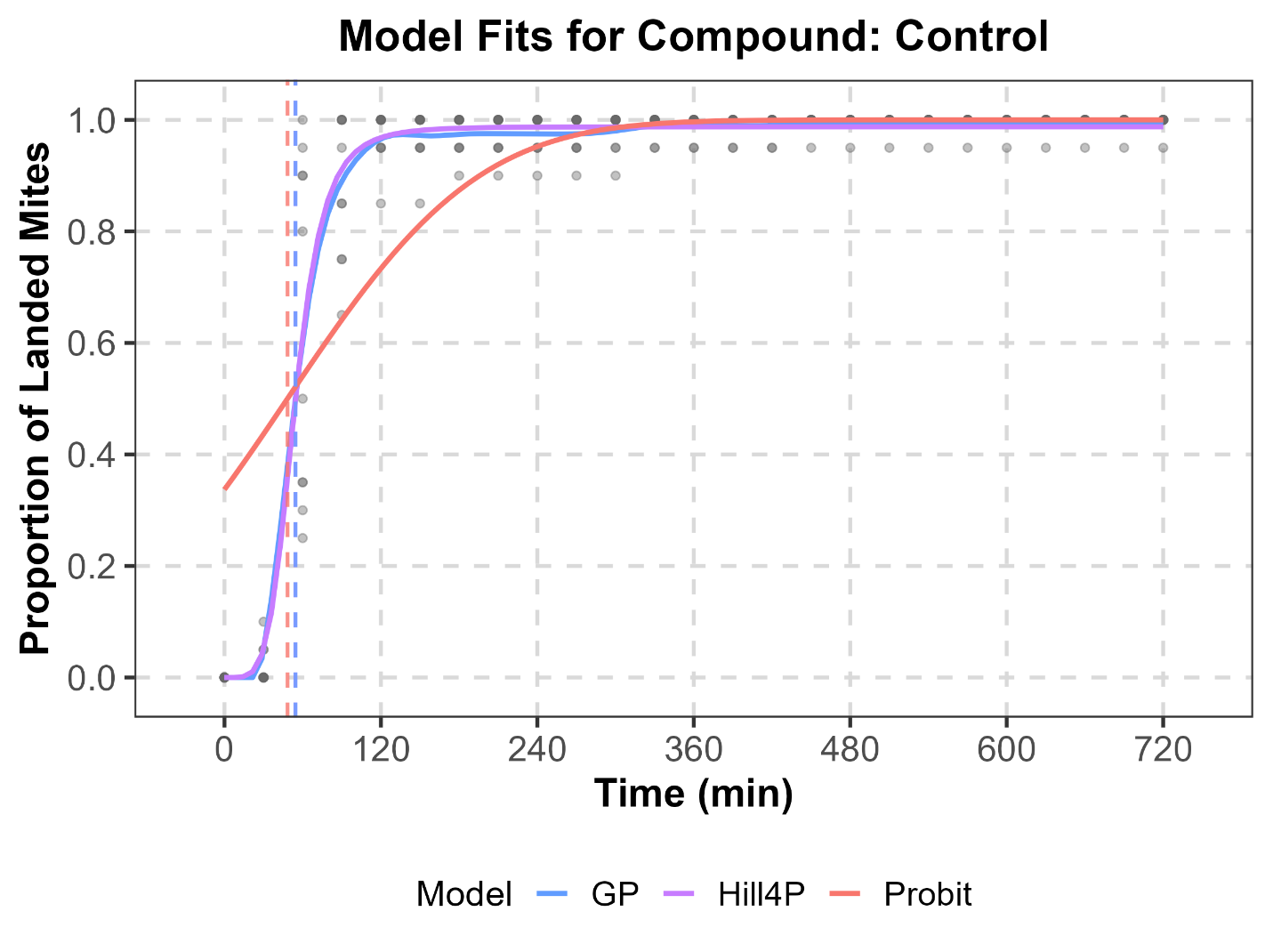


**Supplementary Fig. S21.** Comparison of observed time-dependent landing data for *T. urticae* exposed to solvent control (points) with fitted trajectories from Probit, Hill2P (not converged), Hill4P, and GP models. Dashed vertical lines indicate ET_50_ estimates from each model, where available.

**Supplementary Table S1.** Fit statistics, estimated ET_50_, and estimated AUC of experimental data.

| Compound | Model | RMSE | MAE | R^2^ | ET_50_ (min, 95% CI) | AUC (min, 95% CI) |
| --- | --- | --- | --- | --- | --- | --- |
| t-Anethole | Probit | 0.152 | 0.103 | 0.596 | 650.98 (626.595 - 677.424) | 136.414 (123.597 - 136.476) |
| t-Anethole | Hill2P | 0.152 | 0.101 | 0.597 | 672.412 (634.85 - 725.24) | 137.059 (125.434 - 137.111) |
| t-Anethole | Hill4P | 0.152 | 0.102 | 0.595 | 600.018 (510.02 - 717.321) | 137.834 (126.305 - 137.888) |
| t-Anethole | GP | 0.151 | 0.103 | 0.601 | 669.543 (628.842 - 708.017) | 138.267 (126.409 - 138.326) |
| (+)-Borneol | Probit | 0.190 | 0.141 | 0.576 | 60.081 (9.657 - 105.782) | 578.798 (550.235 - 578.781) |
| (+)-Borneol | Hill2P | 0.129 | 0.084 | 0.800 | 76.314 (61.9 - 88.365) | 599.569 (578.178 - 599.796) |
| (+)-Borneol | Hill4P | 0.125 | 0.079 | 0.810 | 71.704 (60.552 - 87.645) | 588.831 (568.453 - 589.189) |
| (+)-Borneol | GP | 0.127 | 0.081 | 0.806 | 79.999 (62.947 - 94.352) | 589.094 (568.249 - 589.228) |
| Camphor | Probit | 0.121 | 0.060 | 0.898 | 571.765 (560.043 - 582.982) | 148.578 (127.422 - 148.893) |
| Camphor | Hill2P | NA | NA | NA | NA | NA |
| Camphor | Hill4P | NA | NA | NA | NA | NA |
| Camphor | GP | 0.110 | 0.051 | 0.915 | 564.183 (550.829 - 578.487) | 150.238 (130.028 - 150.69) |
| Carvacrol | Probit | 0.142 | 0.075 | 0.870 | 537.702 (524.643 - 549.683) | 182.874 (161.803 - 183.065) |
| Carvacrol | Hill2P | 0.136 | 0.072 | 0.881 | 522.107 (506.435 - 537.849) | 193.197 (172.092 - 193.504) |
| Carvacrol | Hill4P | 0.134 | 0.069 | 0.884 | 511.583 (492.196 - 529.927) | 187.526 (167.907 - 187.818) |
| Carvacrol | GP | 0.134 | 0.070 | 0.883 | 520.435 (499.537 - 537.539) | 188.262 (168.731 - 188.472) |
| β-Caryophyllene | Probit | 0.105 | 0.059 | 0.945 | 408.043 (398.851 - 417.596) | 311.962 (290.353 - 312.161) |
| β-Caryophyllene | Hill2P | 0.098 | 0.054 | 0.952 | 394.128 (383.525 - 405.383) | 318.565 (296.955 - 318.844) |
| β-Caryophyllene | Hill4P | 0.097 | 0.056 | 0.952 | 392.034 (380.079 - 403.696) | 315.947 (294.755 - 316.305) |
| β-Caryophyllene | GP | 0.097 | 0.056 | 0.952 | 394 (379.929 - 406.819) | 315.874 (294.628 - 316.142) |
| 1,8-Cineole | Probit | 0.070 | 0.024 | 0.106 | NA | 9.658 (7.625 - 9.707) |
| 1,8-Cineole | Hill2P | 0.069 | 0.024 | 0.111 | 1080 (771.814 - 1080) | 10.216 (8.408 - 10.248) |
| 1,8-Cineole | Hill4P | NA | NA | NA | NA | NA |
| 1,8-Cineole | GP | 0.069 | 0.025 | 0.112 | NA | 10.892 (9.398 - 10.907) |
| t-Cinnamaldehyde | Probit | 0.034 | 0.013 | 0.117 | NA | 5.151 (4.067 - 5.177) |
| t-Cinnamaldehyde | Hill2P | NA | NA | NA | NA | NA |
| t-Cinnamaldehyde | Hill4P | NA | NA | NA | NA | NA |
| t-Cinnamaldehyde | GP | 0.034 | 0.014 | 0.131 | NA | 5.8 (4.986 - 5.807) |
| Citral | Probit | 0.199 | 0.163 | 0.458 | 153.992 (82.805 - 198.785) | 480.651 (454.633 - 480.645) |
| Citral | Hill2P | 0.157 | 0.133 | 0.663 | 111.731 (94.099 - 129.758) | 495.521 (476.426 - 495.451) |
| Citral | Hill4P | 0.149 | 0.127 | 0.696 | 76.825 (64.758 - 92.28) | 491.275 (473.998 - 491.577) |
| Citral | GP | 0.149 | 0.128 | 0.697 | 99.855 (77.144 - 119.738) | 490.121 (472.155 - 490.302) |
| Citronellal | Probit | 0.106 | 0.043 | 0.924 | 570.021 (560.742 - 579.547) | 149.996 (128.411 - 150.474) |
| Citronellal | Hill2P | 0.106 | 0.043 | 0.924 | 568.885 (556.379 - 580.295) | 149.181 (127.733 - 149.627) |
| Citronellal | Hill4P | 0.106 | 0.044 | 0.924 | 569.934 (554.955 - 583.649) | 148.07 (126.744 - 148.467) |
| Citronellal | GP | 0.106 | 0.044 | 0.923 | 569.833 (556.944 - 584.467) | 150.534 (128.83 - 150.911) |
| Citronellol | Probit | 0.196 | 0.149 | 0.644 | 177.409 (138.857 - 207.677) | 506.547 (483.538 - 506.541) |
| Citronellol | Hill2P | 0.147 | 0.107 | 0.800 | 135.205 (118.654 - 150.75) | 529.079 (508.279 - 529.238) |
| Citronellol | Hill4P | 0.138 | 0.096 | 0.821 | 119.155 (103.464 - 134.027) | 516.88 (497.739 - 517.235) |
| Citronellol | GP | 0.136 | 0.093 | 0.827 | 129.714 (114.688 - 145.189) | 516.387 (496.391 - 516.716) |
| Eugenol | Probit | 0.093 | 0.044 | 0.841 | 656.312 (645.086 - 667.288) | 76.925 (61.485 - 77.159) |
| Eugenol | Hill2P | 0.092 | 0.044 | 0.845 | 653.731 (639.696 - 666.041) | 80.598 (65.572 - 80.799) |
| Eugenol | Hill4P | 0.091 | 0.041 | 0.848 | 617.259 (591.562 - 665.361) | 79.003 (64.603 - 79.243) |
| Eugenol | GP | 0.091 | 0.042 | 0.848 | 650.748 (629.115 - 667.21) | 78.917 (59.962 - 79.11) |
| Geranic acid | Probit | 0.124 | 0.103 | 0.804 | 446.865 (432.549 - 461.427) | 289.849 (271.86 - 289.885) |
| Geranic acid | Hill2P | 0.109 | 0.085 | 0.850 | 395.721 (378.225 - 411.34) | 297.943 (281.821 - 298.006) |
| Geranic acid | Hill4P | 0.109 | 0.085 | 0.850 | 390.007 (303.329 - 450.012) | 297.637 (281.549 - 297.703) |
| Geranic acid | GP | 0.099 | 0.074 | 0.876 | 458.521 (373.356 - 493.924) | 295.229 (274.02 - 295.4) |
| D-Limonene | Probit | 0.157 | 0.119 | 0.786 | 304.099 (288.301 - 319.977) | 411.327 (389.729 - 411.354) |
| D-Limonene | Hill2P | 0.167 | 0.137 | 0.758 | 257.972 (237.755 - 280.889) | 399.548 (380.757 - 399.648) |
| D-Limonene | Hill4P | 0.174 | 0.149 | 0.778 | 335.358 (293.586 - 429.325) | 370.764 (352.755 - 370.82) |
| D-Limonene | GP | 0.111 | 0.075 | 0.893 | 321.848 (223.277 - 450.801) | 409.828 (388.287 - 410.292) |
| Linalool | Probit | 0.131 | 0.100 | 0.855 | 212.273 (197.369 - 226.671) | 495.608 (473.108 - 495.629) |
| Linalool | Hill2P | 0.136 | 0.097 | 0.850 | 196.055 (174.755 - 217.342) | 482.667 (461.739 - 482.822) |
| Linalool | Hill4P | 0.118 | 0.087 | 0.880 | 251.629 (221.522 - 263.931) | 497.929 (475.671 - 498.175) |
| Linalool | GP | 0.103 | 0.074 | 0.909 | 240.935 (228.638 - 252.921) | 501.102 (480.093 - 501.336) |
| Linalyl acetate | Probit | 0.079 | 0.037 | 0.920 | 622.313 (614.515 - 630.384) | 102.286 (83.806 - 102.568) |
| Linalyl acetate | Hill2P | 0.077 | 0.037 | 0.925 | 615.23 (605.862 - 624.036) | 107.257 (89.144 - 107.488) |
| Linalyl acetate | Hill4P | 0.075 | 0.033 | 0.928 | 595.15 (577.444 - 613.649) | 105.407 (88.179 - 105.717) |
| Linalyl acetate | GP | 0.075 | 0.033 | 0.928 | 610.898 (595.878 - 623.395) | 104.709 (87.304 - 104.949) |
| L-Menthol | Probit | 0.139 | 0.086 | 0.656 | 652.576 (630.211 - 679.339) | 112.836 (99.49 - 112.933) |
| L-Menthol | Hill2P | 0.134 | 0.088 | 0.679 | 660.327 (629.122 - 702.832) | 120.269 (107.957 - 120.339) |
| L-Menthol | Hill4P | 0.126 | 0.079 | 0.717 | 482.172 (458.563 - 513.113) | 116.39 (105.859 - 116.542) |
| L-Menthol | GP | 0.126 | 0.079 | 0.715 | NA | 116.464 (105.91 - 116.569) |
| α-Pinene | Probit | 0.102 | 0.078 | 0.890 | 202.27 (189.948 - 214.704) | 502.174 (479.52 - 502.183) |
| α-Pinene | Hill2P | 0.119 | 0.094 | 0.856 | 163.257 (149.088 - 180.097) | 490.398 (470.305 - 490.573) |
| α-Pinene | Hill4P | 0.128 | 0.108 | 0.880 | 232.645 (201.699 - 269.766) | 459.134 (437.841 - 459.18) |
| α-Pinene | GP | 0.086 | 0.064 | 0.923 | 186.864 (148.914 - 253.156) | 506.182 (484.936 - 506.225) |
| (-)-Terpinen-4-ol | Probit | 0.051 | 0.024 | 0.162 | NA | 10.517 (8.575 - 10.549) |
| (-)-Terpinen-4-ol | Hill2P | NA | NA | NA | NA | NA |
| (-)-Terpinen-4-ol | Hill4P | NA | NA | NA | NA | NA |
| (-)-Terpinen-4-ol | GP | 0.050 | 0.025 | 0.187 | NA | 11.52 (10.062 - 11.533) |
| α-Terpineol | Probit | 0.148 | 0.118 | 0.734 | 455.281 (437.736 - 472.216) | 283.717 (265.597 - 283.75) |
| α-Terpineol | Hill2P | 0.136 | 0.107 | 0.775 | 407.737 (386.604 - 428.483) | 290.823 (274.964 - 290.883) |
| α-Terpineol | Hill4P | 0.136 | 0.106 | 0.775 | 389.865 (271.88 - 480.872) | 290.69 (269.758 - 290.747) |
| α-Terpineol | GP | 0.126 | 0.096 | 0.808 | 425.953 (316.123 - 532.273) | 287.168 (270.119 - 287.303) |
| Thymol | Probit | 0.111 | 0.058 | 0.203 | NA | 30.062 (25.406 - 30.12) |
| Thymol | Hill2P | 0.109 | 0.061 | 0.221 | 1080 (913.174 - 1080) | 32.825 (28.905 - 32.857) |
| Thymol | Hill4P | NA | NA | NA | NA | NA |
| Thymol | GP | 0.108 | 0.060 | 0.235 | NA | 32.242 (28.734 - 32.271) |
| Control | Probit | 0.150 | 0.081 | 0.739 | 48.359 (11.669 - 73.286) | 645.941 (623.458 - 645.909) |
| Control | Hill4P | 0.069 | 0.034 | 0.939 | 54.375 (49.692 - 62.332) | 653.699 (632.184 - 655.099) |
| Control | GP | 0.068 | 0.032 | 0.940 | 54.47 (47.071 - 63.741) | 653.492 (631.798 - 654.976) |
| Control | Hill | 0.069 | 0.029 | 0.939 | 54.556 (48.808 - 62.3) | 661.034 (639.371 - 662.251) |

**Supplementary Table S2.** Parameters used for synthetic data generation. Scenarios A and B were generated using a parametric Hill equation. Scenarios C and D were generated using a biphasic model, created by integrating two Hill equations, to simulate two-phase landing behavior. To ensure a robust evaluation across a variety of conditions, parameter values were deliberately selected to represent a diverse range of hypothetical time-response curves.

| Scenarios | Parameter | Description |
| --- | --- | --- |
| A & B | *t_start* | Time delay before the first landing occurs |
|  | *ET50* | Time after *t_start* to reach 50% of the maximum landing proportion |
|  | *slope* | The steepness (Hill slope) of the landing curve |
|  | *max_proportion^a^* | The asymptotic maximum landing proportion |
| C & D | *t_start_1* | Start time of the first landing phase |
|  | *ET50_1* | The time after *t_start_1* to reach 50% of the first phase's maximum landing proportion |
|  | *slope_1* | The Hill slope of the initial landing curve |
|  | *max_proportion_1* | The proportion of mites that land during this first phase, forming a temporary plateau |
|  | *t_start_2* | The start time of the second landing phase (the location of spline, larger than *t_start_1*) |
|  | *ET50_2* | The time after *t_start_2* to reach 50% of the remaining mites' landing |
|  | *slope_2* | The Hill slope of the secondary landing phase |
|  | *max_proportion_2^a^* | The total cumulative landing proportion after both phases are complete |

^a^Set to 1.0 for Scenario A and C (Complete landing) and < 1.0 for Scenario B and D (Incomplete Landing).

**Supplementary Table S3.** Parameter settings used in simulating the four mite landing scenarios described in Table S2.

| Scenario | Parameters (± SD) | | | | | | | |
| --- | --- | --- | --- | --- | --- | --- | --- | --- |
|  | t_start(_1) | ET50(_1) | slope(_1) | max_proportion(_1) | t_start_2 | ET50_2 | slope_2 | max_proportion_2 |
| A1 | 50 (20) | 130 (30) | 2.5 (0.3) | - | - | - | - | - |
| A2 | 150 (30) | 140 (40) | 3.0 (0.5) | - | - | - | - | - |
| A3 | 500 (30) | 40 (10) | 6.3 (1.0) | - | - | - | - | - |
| A4 | 170 (50) | 200 (60) | 2.8 (0.5) | - | - | - | - | - |
| A5 | 150 (50) | 220 (60) | 2.2 (0.5) | - | - | - | - | - |
| B1 | 550 (30) | 70 (30) | 3.5 (1.0) | 0.7 (0.2) | - | - | - | - |
| B2 | 100 (25) | 50 (20) | 3.5 (1.0) | 0.7 (0.2) | - | - | - | - |
| B3 | 290 (30) | 80 (30) | 2.8 (1.0) | 0.8 (0.2) | - | - | - | - |
| B4 | 130 (30) | 60 (20) | 4.0 (1.0) | 0.8 (0.2) | - | - | - | - |
| B5 | 280 (50) | 270 (60) | 1.6 (0.5) | 0.8 (0.2) | - | - | - | - |
| C1 | 50 (20) | 120 (20) | 2.0 (0.5) | 0.4 (0.1) | 400 (50) | 550 (50) | 3.0 (1.0) | - |
| C2 | 20 (10) | 90 (20) | 2.0 (0.5) | 0.5 (0.2) | 300 (30) | 450 (30) | 3.0 (0.5) | - |
| C3 | 130 (20) | 130 (30) | 5.0 (1.0) | 0.5 (0.2) | 350 (20) | 550 (50) | 3.0 (0.5) | - |
| C4 | 80 (30) | 120 (30) | 3.5 (1.0) | 0.5 (0.2) | 350 (50) | 550 (50) | 3.5 (1.0) | - |
| C5 | 150 (50) | 180 (20) | 2.5 (0.5) | 0.6 (0.2) | 500 (50) | 660 (30) | 5.0 (1.0) | - |
| D1 | 10 (10) | 60 (20) | 2.5 (1.0) | 0.3 (0.2) | 360 (30) | 400 (40) | 2.0 (0.5) | 0.8 (0.1) |
| D2 | 130 (50) | 140 (30) | 2.0 (0.5) | 0.5 (0.1) | 360 (30) | 400 (40) | 1.5 (0.3) | 0.7 (0.1) |
| D3 | 60 (20) | 80 (20) | 2.0 (0.5) | 0.3 (0.1) | 200 (30) | 280 (30) | 1.5 (0.3) | 0.7 (0.1) |
| D4 | 170 (50) | 200 (50) | 3.5 (1.0) | 0.5 (0.2) | 300 (30) | 380 (30) | 2.0 (0.5) | 0.8 (0.1) |
| D5 | 40 (20) | 70 (30) | 2.5 (1.0) | 0.4 (0.1) | 380 (30) | 550 (50) | 2.5 (0.5) | 0.7 (0.1) |


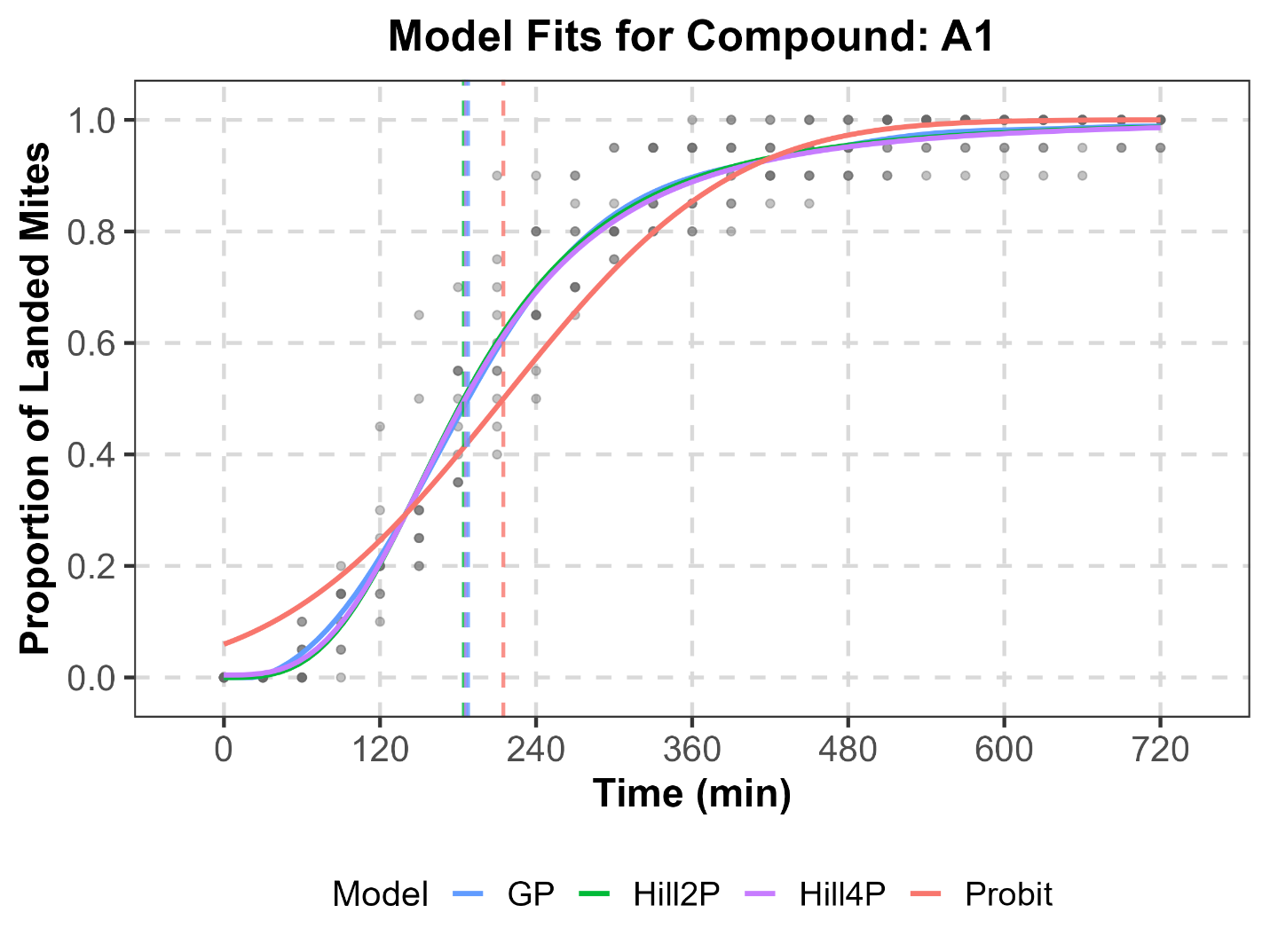


**Supplementary Fig. S22.** Comparison of simulated time-dependent landing data for *T. urticae* exposed to A1 (points) with fitted trajectories from Probit, Hill2P, Hill4P, and GP models. Dashed vertical lines indicate ET_50_ estimates from each model, where available.


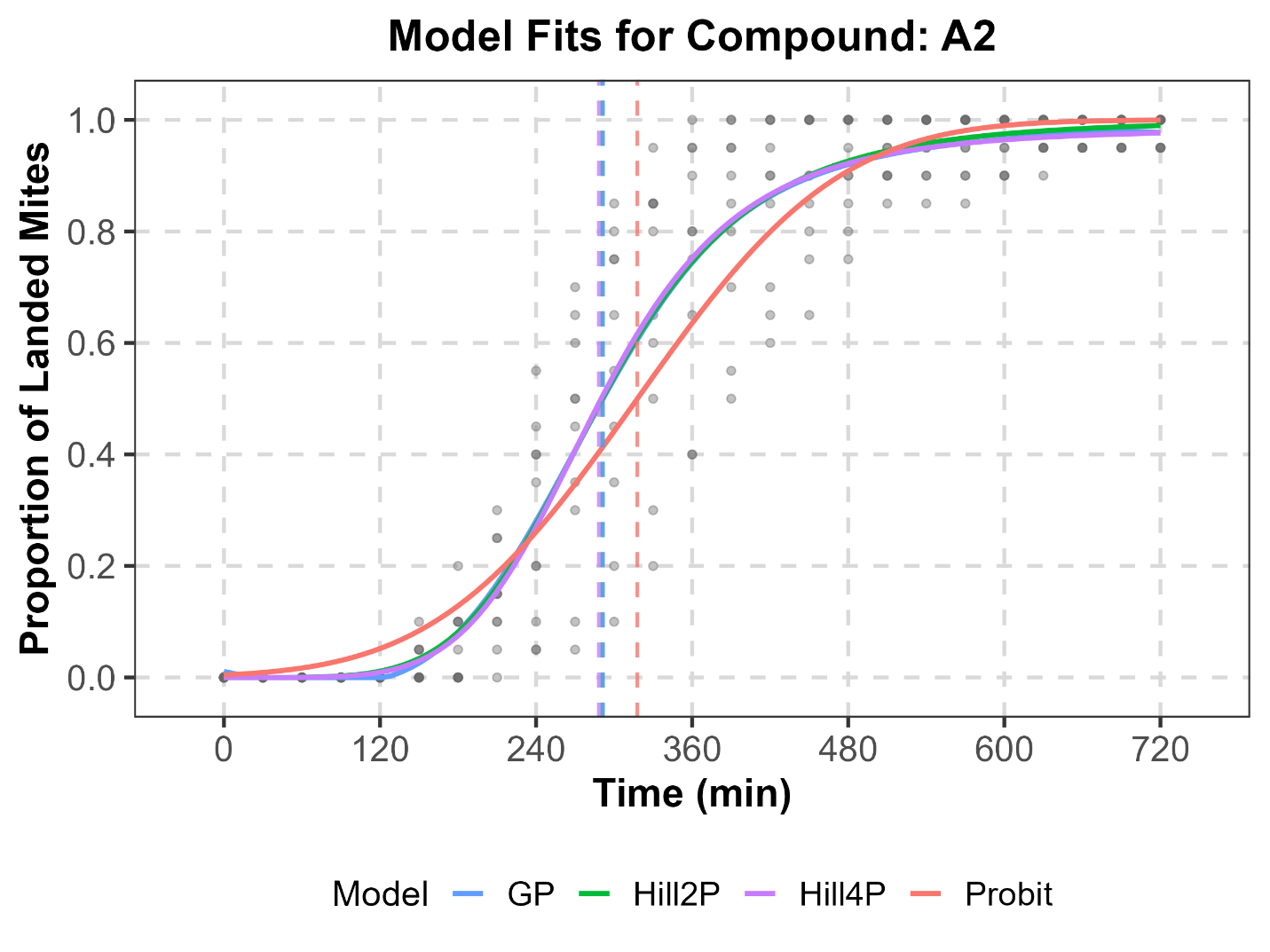


**Supplementary Fig. S23.** Comparison of simulated time-dependent landing data for *T. urticae* exposed to A2 (points) with fitted trajectories from Probit, Hill2P, Hill4P, and GP models. Dashed vertical lines indicate ET_50_ estimates from each model, where available.


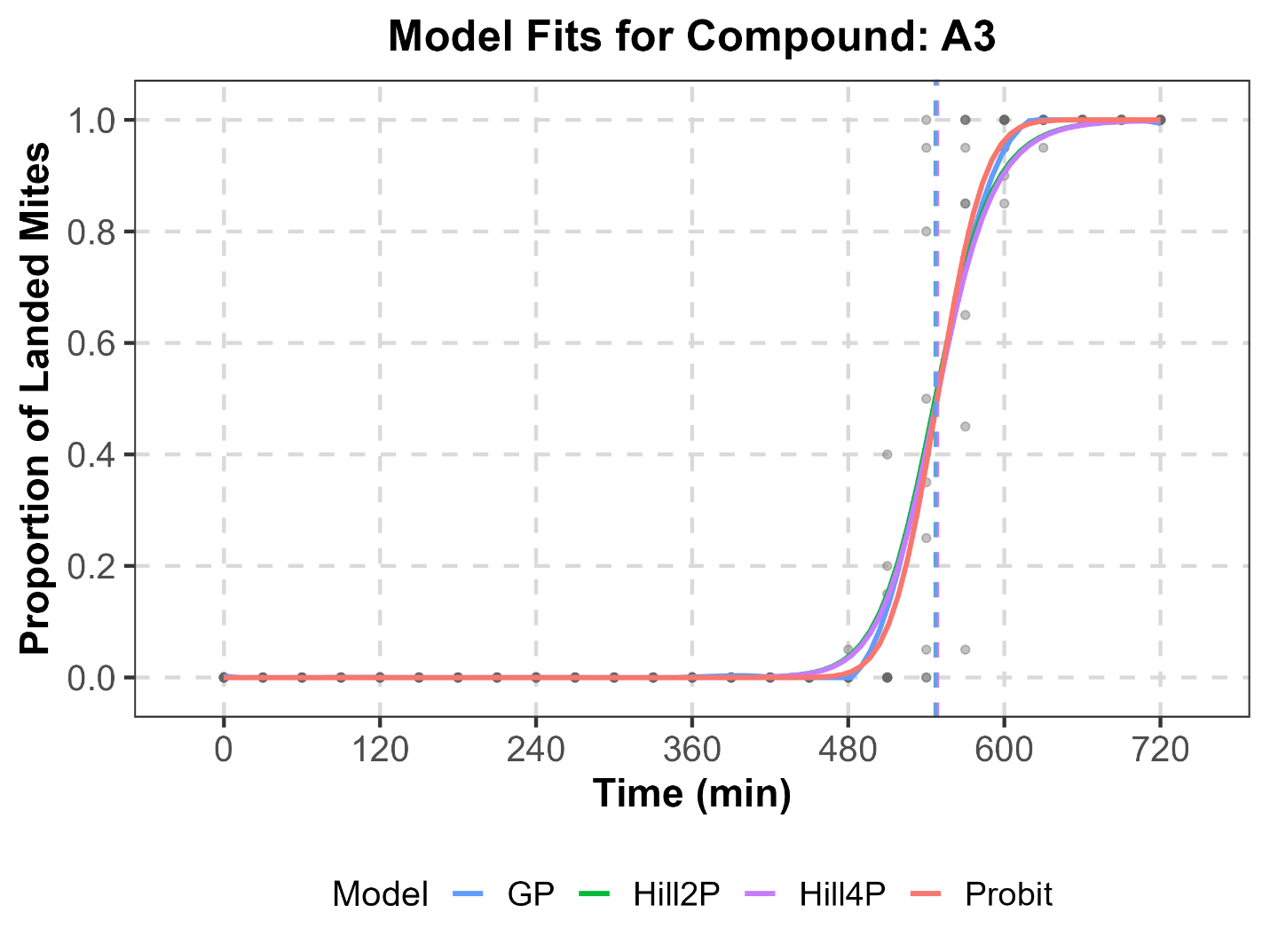


**Supplementary Fig. S23.** Comparison of simulated time-dependent landing data for *T. urticae* exposed to A3 (points) with fitted trajectories from Probit, Hill2P, Hill4P, and GP models. Dashed vertical lines indicate ET_50_ estimates from each model, where available.


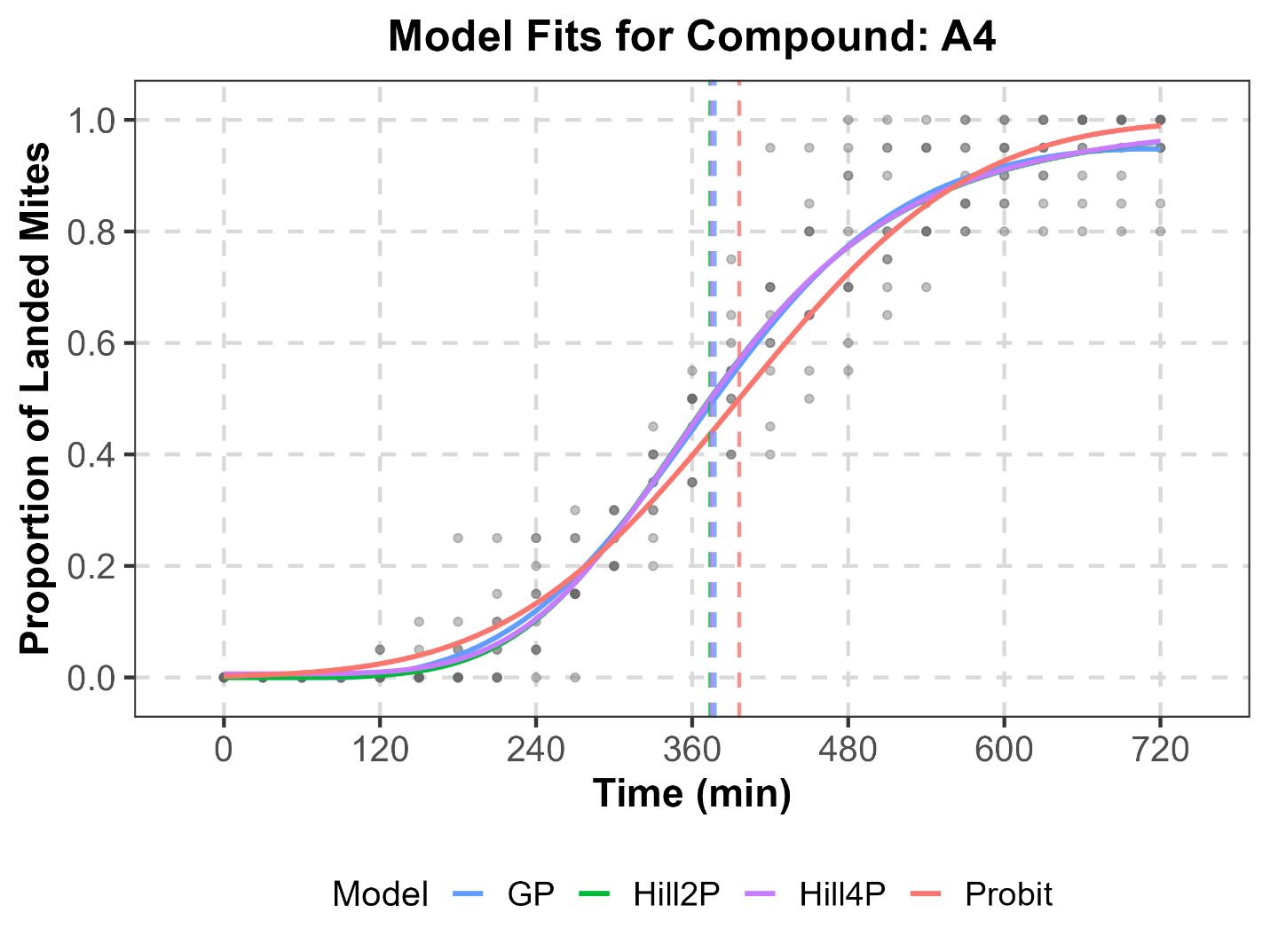


**Supplementary Fig. S24.** Comparison of simulated time-dependent landing data for *T. urticae* exposed to A4 (points) with fitted trajectories from Probit, Hill2P, Hill4P, and GP models. Dashed vertical lines indicate ET_50_ estimates from each model, where available.


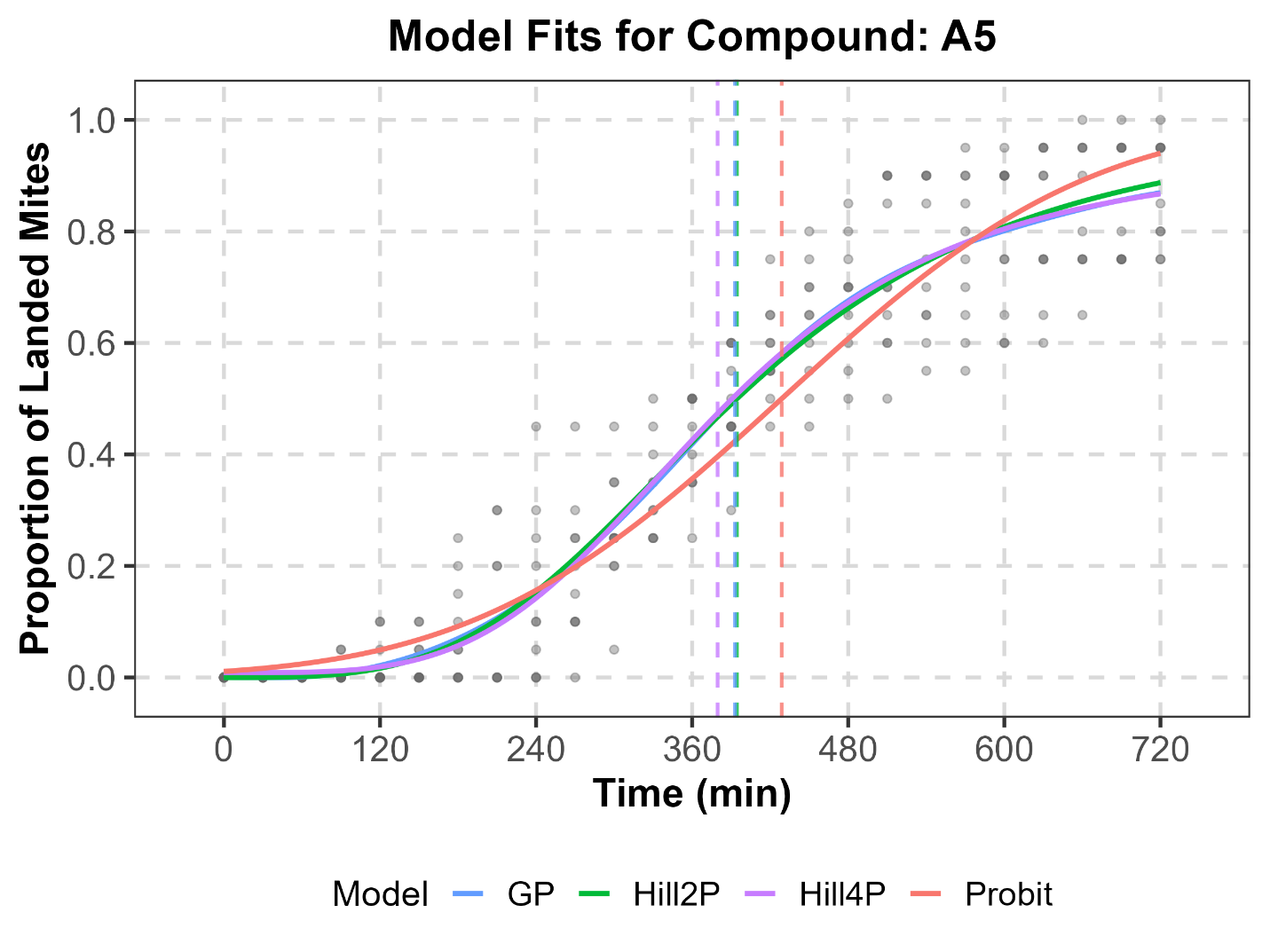


**Supplementary Fig. S25.** Comparison of simulated time-dependent landing data for *T. urticae* exposed to A5 (points) with fitted trajectories from Probit, Hill2P, Hill4P, and GP models. Dashed vertical lines indicate ET_50_ estimates from each model, where available.


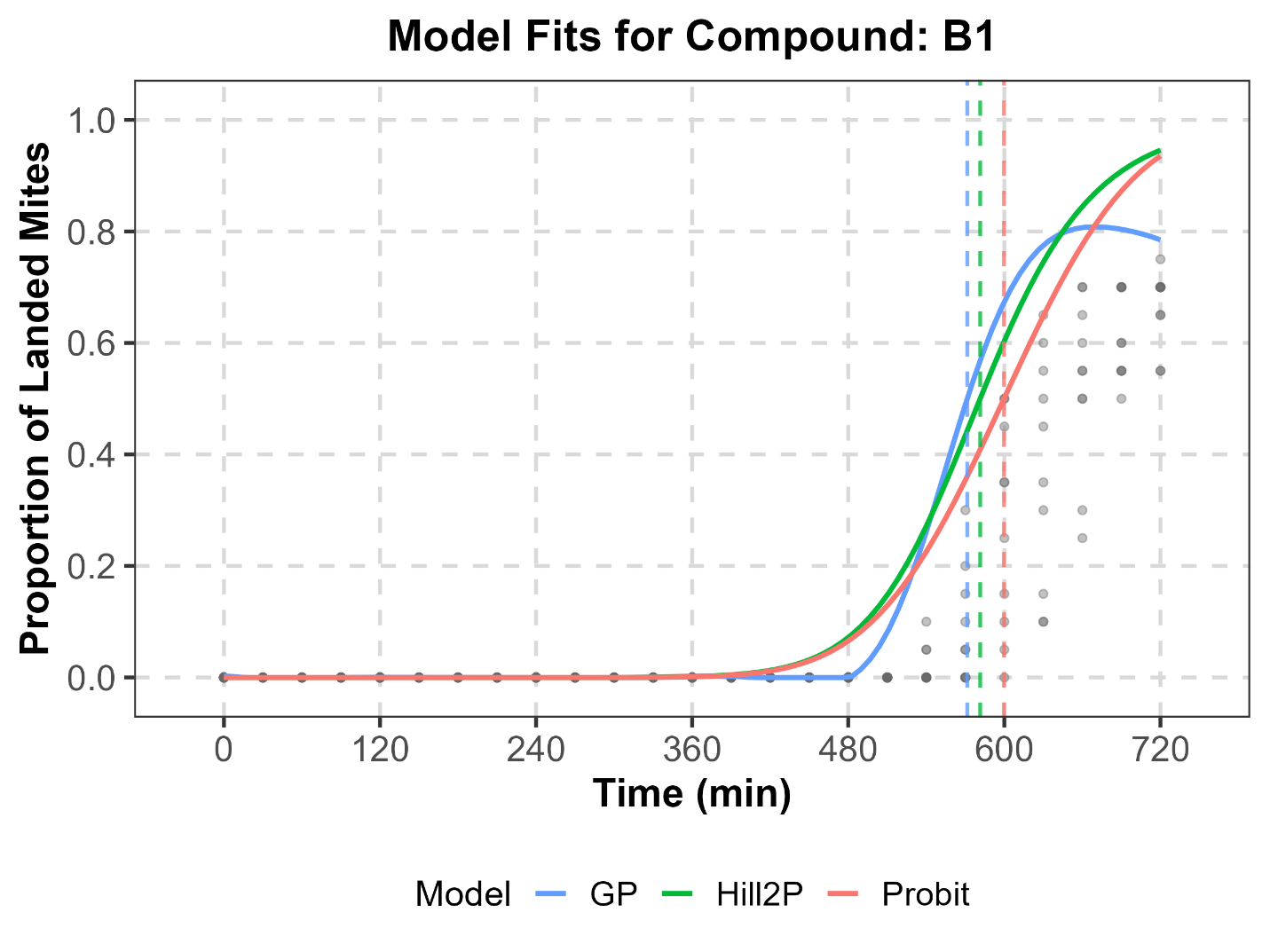


**Supplementary Fig. S26.** Comparison of simulated time-dependent landing data for *T. urticae* exposed to B1 (points) with fitted trajectories from Probit, Hill2P, Hill4P (not converged), and GP models. Dashed vertical lines indicate ET_50_ estimates from each model, where available.


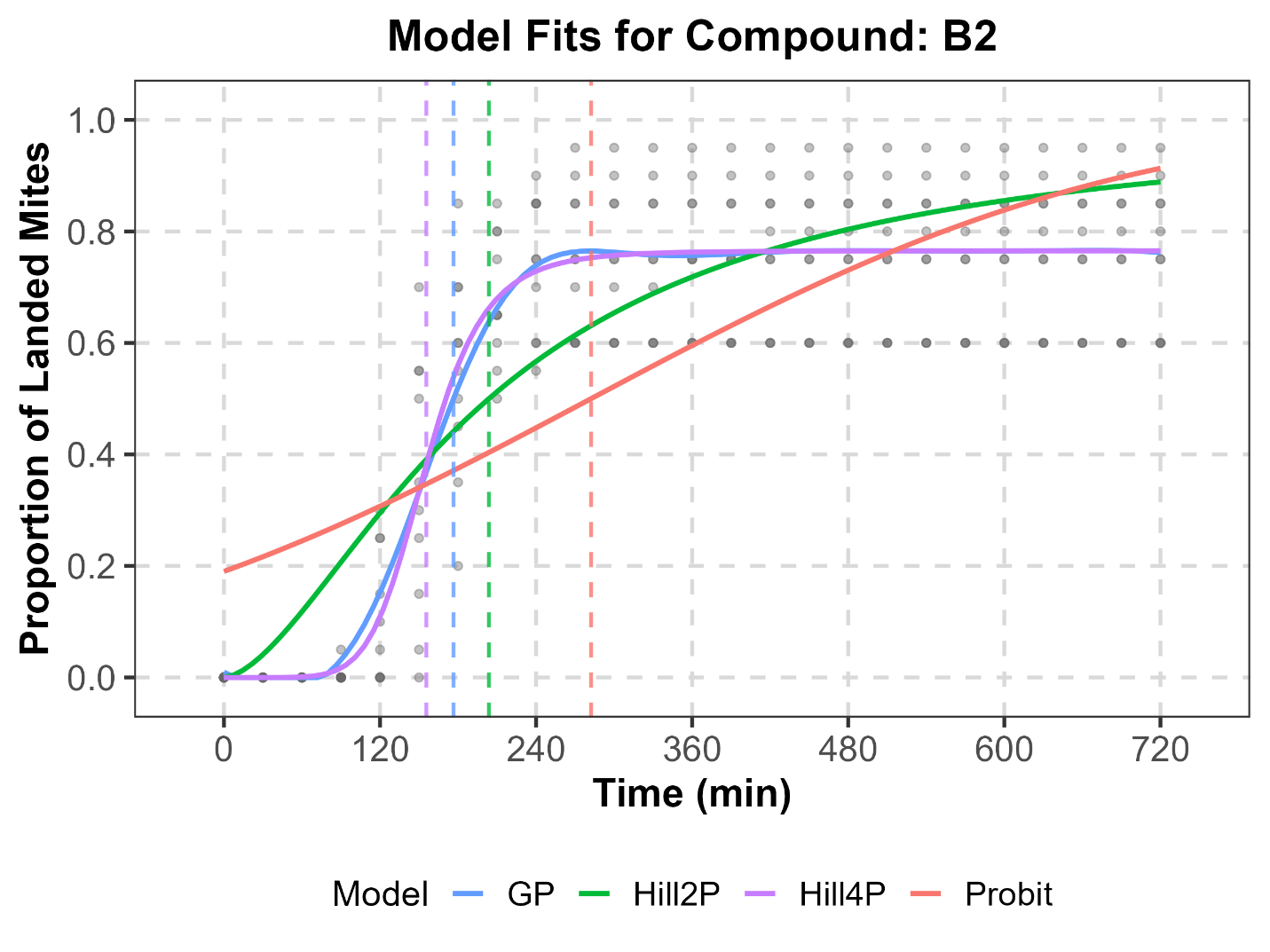


**Supplementary Fig. S27.** Comparison of simulated time-dependent landing data for *T. urticae* exposed to B2 (points) with fitted trajectories from Probit, Hill2P, Hill4P, and GP models. Dashed vertical lines indicate ET_50_ estimates from each model, where available.


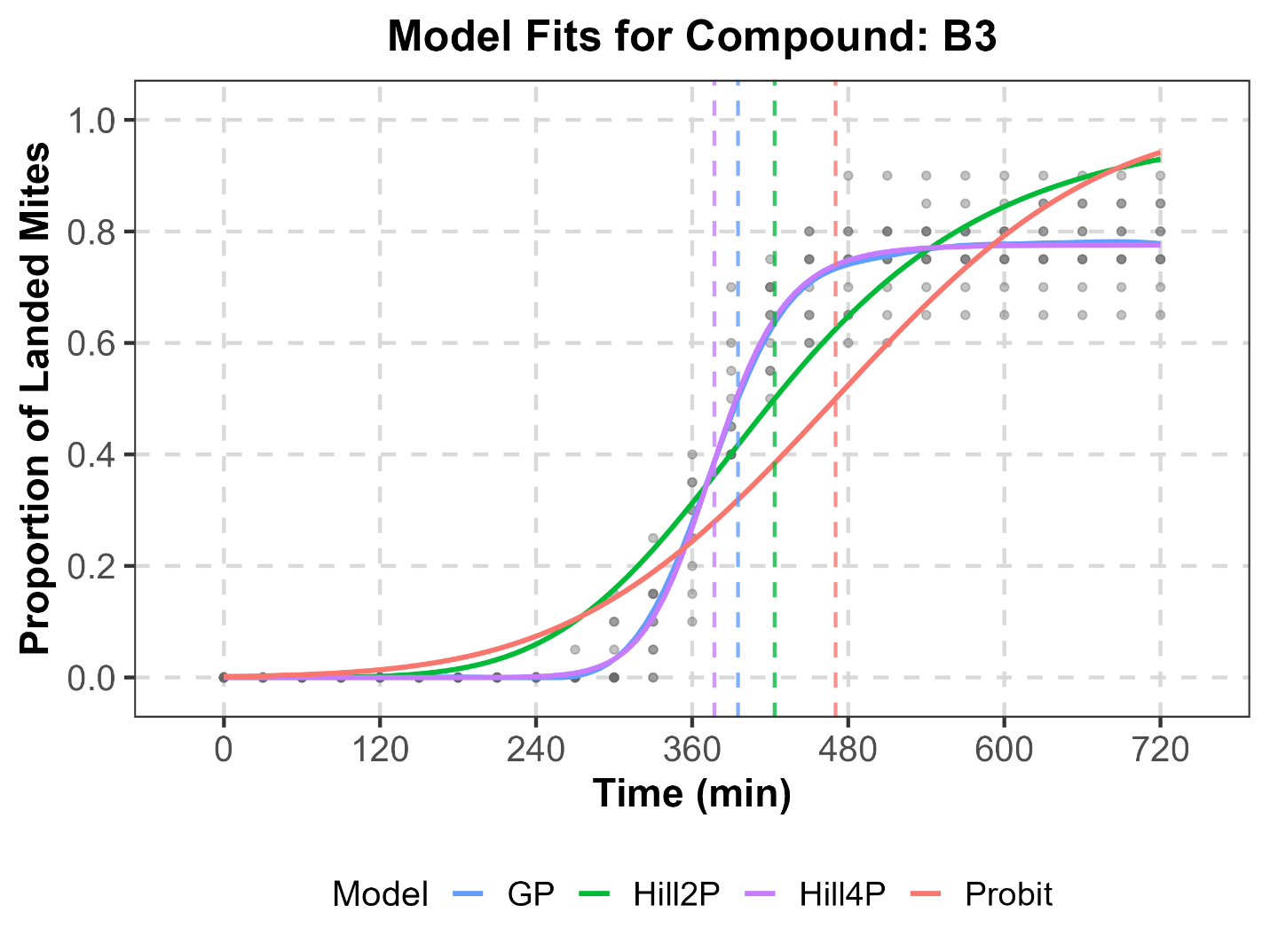


**Supplementary Fig. S28.** Comparison of simulated time-dependent landing data for *T. urticae* exposed to B3 (points) with fitted trajectories from Probit, Hill2P, Hill4P, and GP models. Dashed vertical lines indicate ET_50_ estimates from each model, where available.


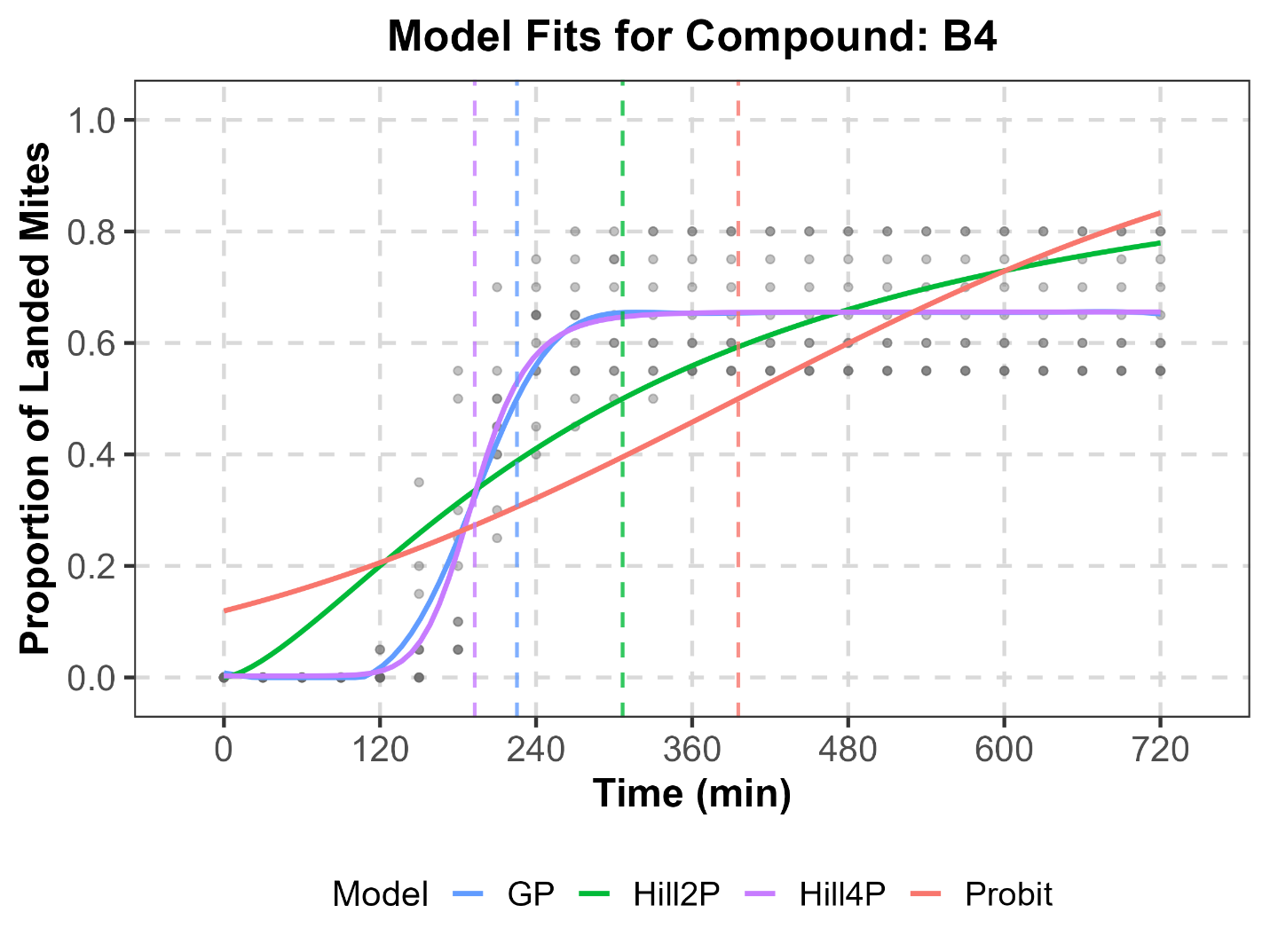


**Supplementary Fig. S29.** Comparison of simulated time-dependent landing data for *T. urticae* exposed to B4 (points) with fitted trajectories from Probit, Hill2P, Hill4P, and GP models. Dashed vertical lines indicate ET_50_ estimates from each model, where available.


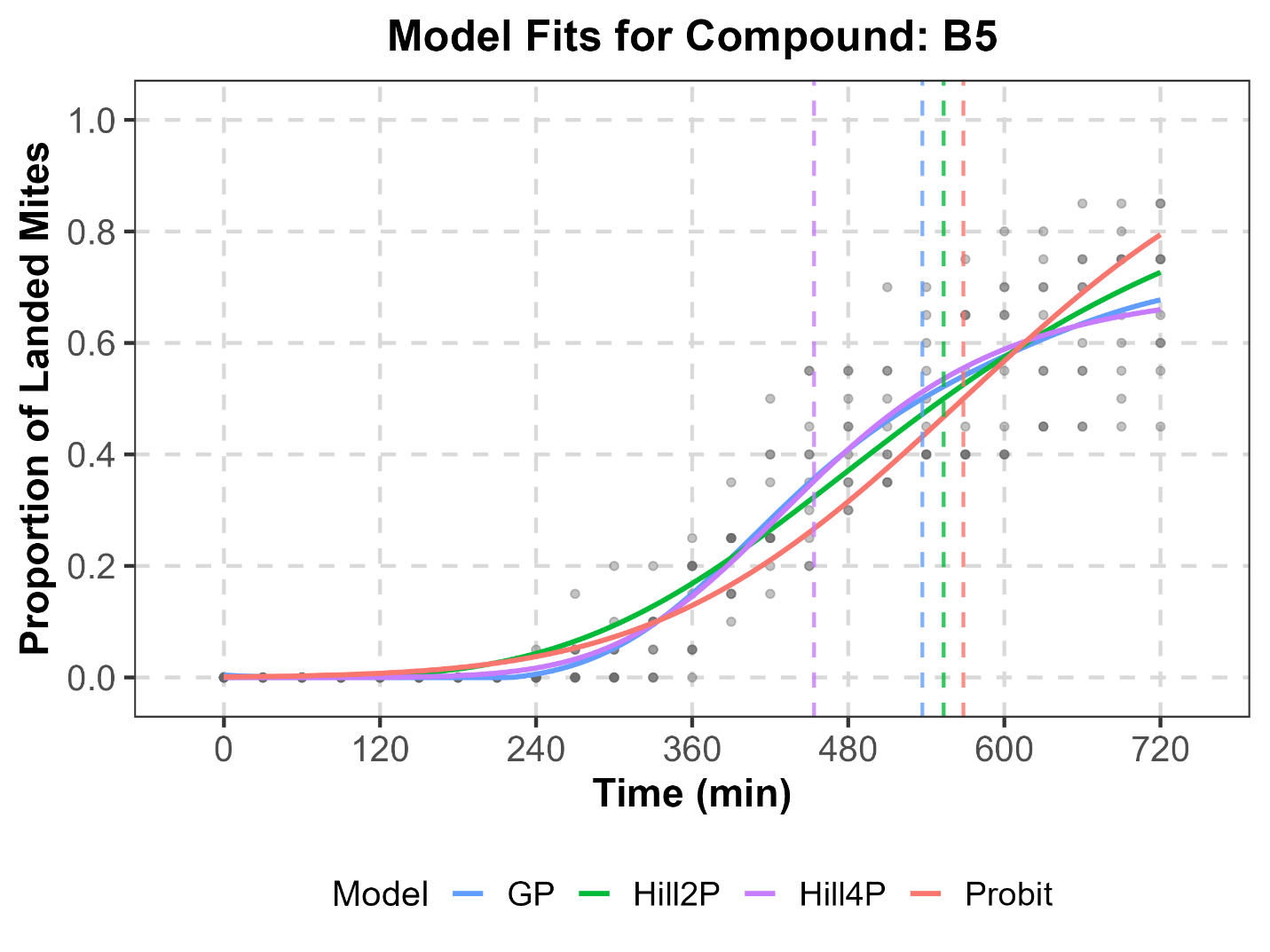


**Supplementary Fig. S30.** Comparison of simulated time-dependent landing data for *T. urticae* exposed to B5 (points) with fitted trajectories from Probit, Hill2P, Hill4P, and GP models. Dashed vertical lines indicate ET_50_ estimates from each model, where available.


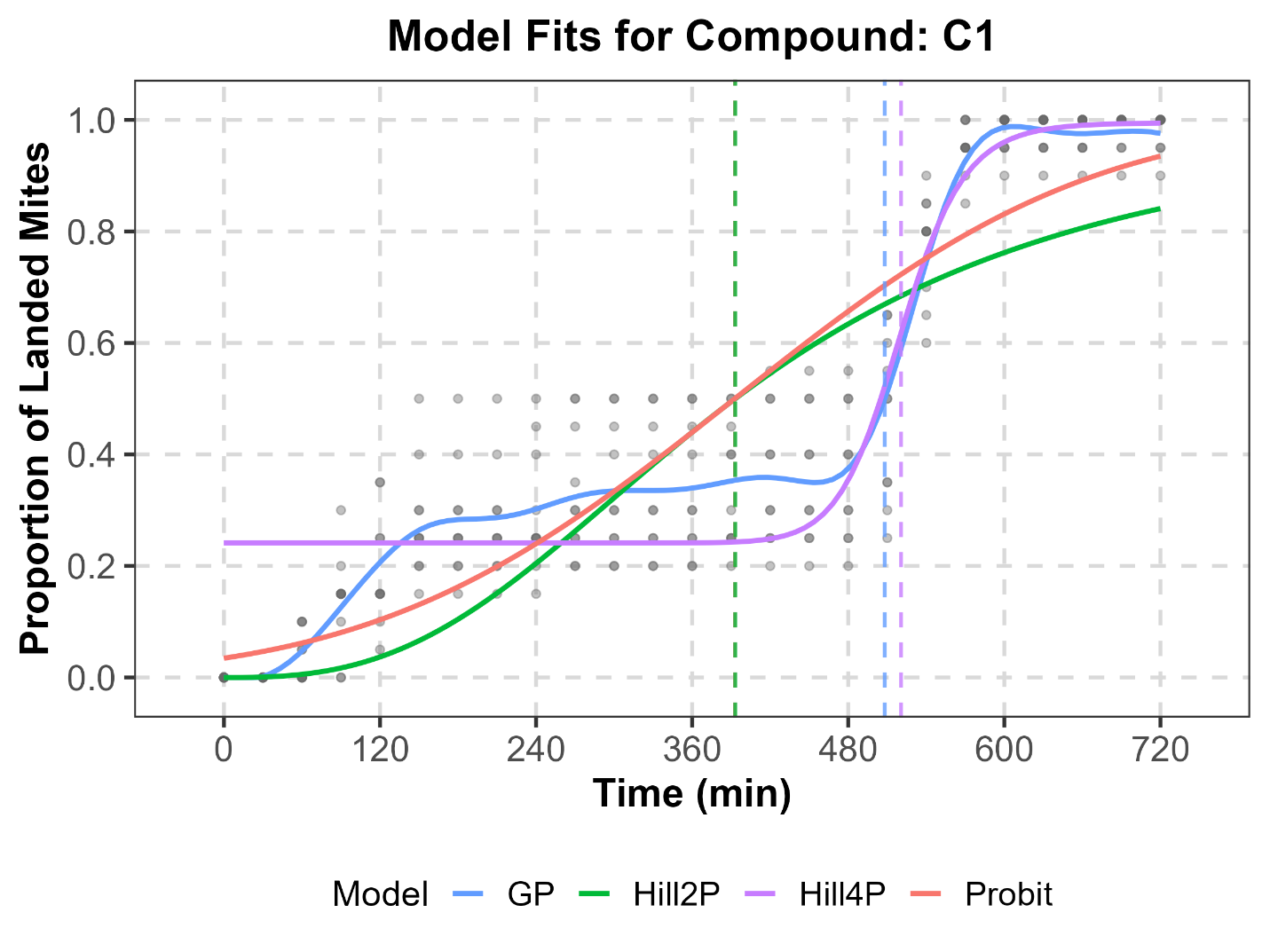


**Supplementary Fig. S31.** Comparison of simulated time-dependent landing data for *T. urticae* exposed to C1 (points) with fitted trajectories from Probit, Hill2P, Hill4P, and GP models. Dashed vertical lines indicate ET_50_ estimates from each model, where available.


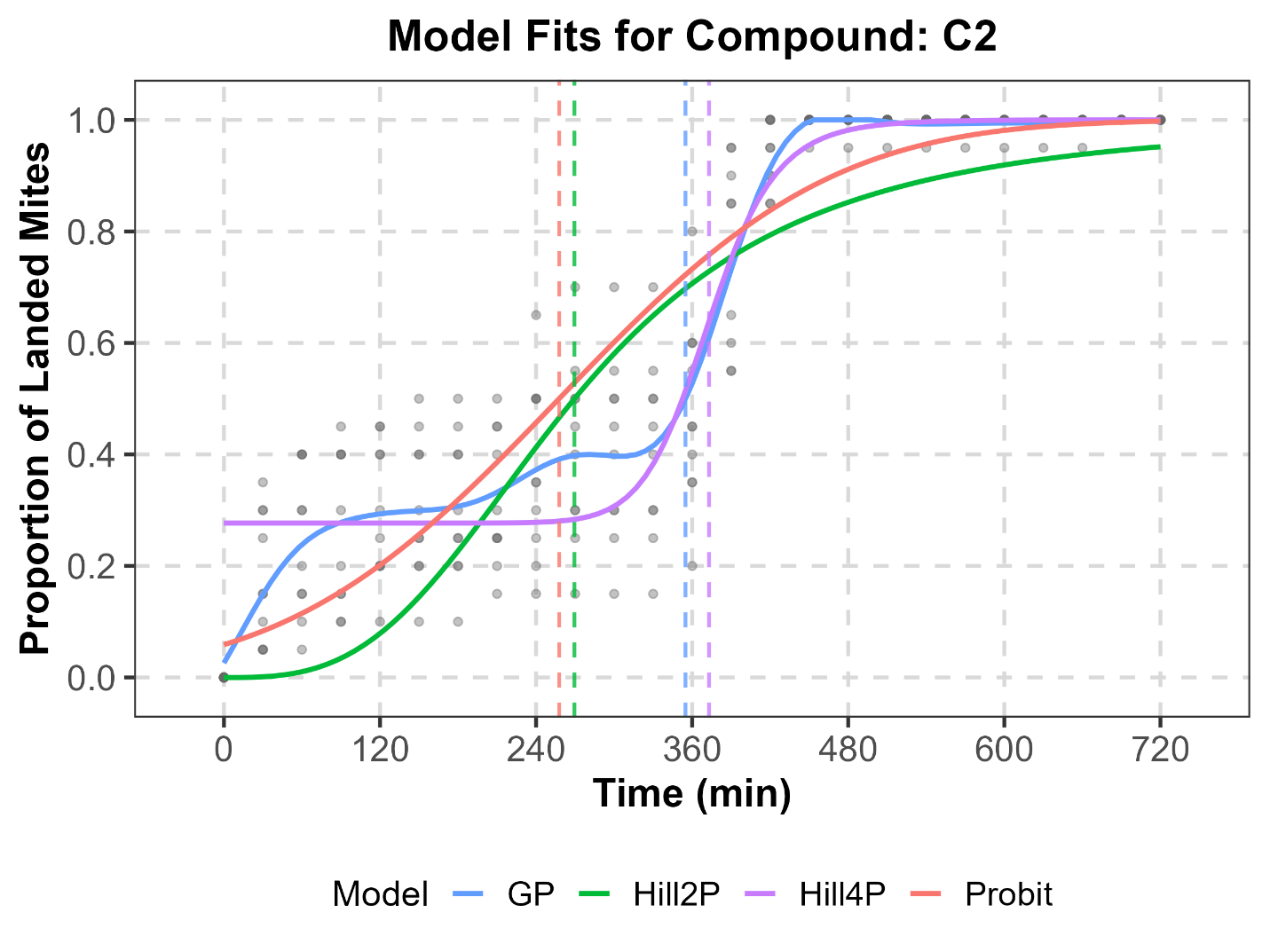


**Supplementary Fig. S32.** Comparison of simulated time-dependent landing data for *T. urticae* exposed to C2 (points) with fitted trajectories from Probit, Hill2P, Hill4P, and GP models. Dashed vertical lines indicate ET_50_ estimates from each model, where available.


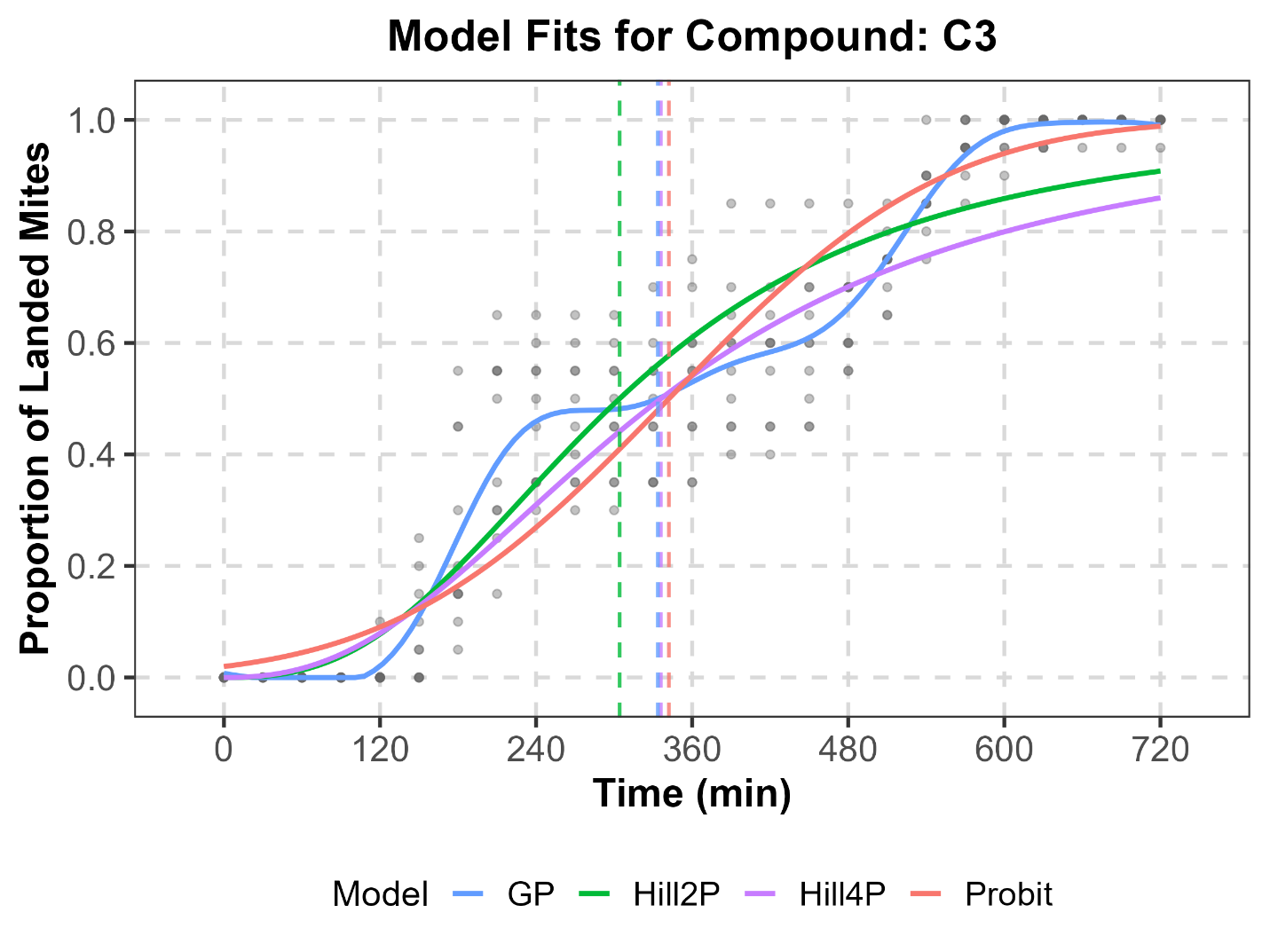


**Supplementary Fig. S33.** Comparison of simulated time-dependent landing data for *T. urticae* exposed to C3 (points) with fitted trajectories from Probit, Hill2P, Hill4P, and GP models. Dashed vertical lines indicate ET_50_ estimates from each model, where available.


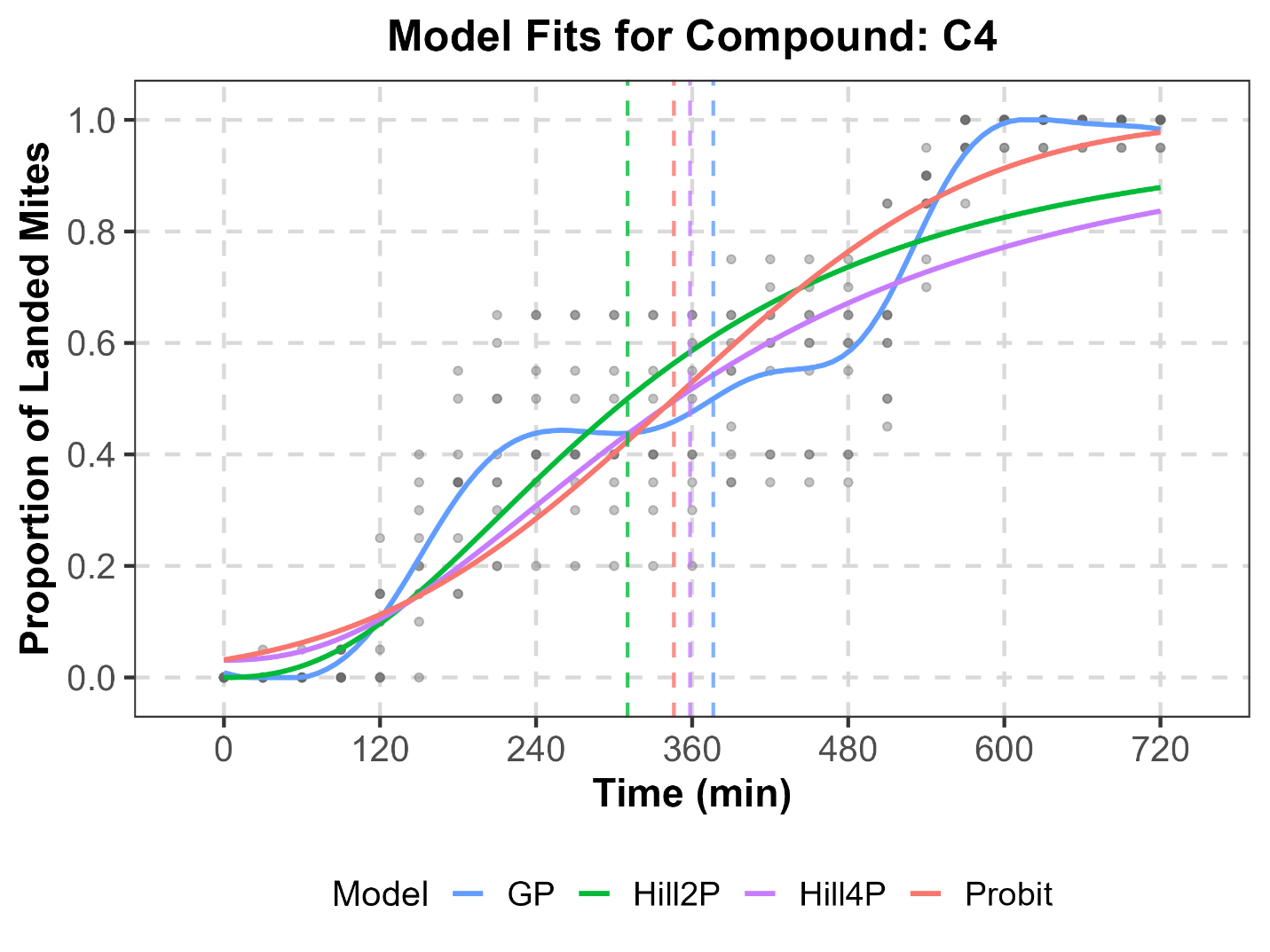


**Supplementary Fig. S34.** Comparison of simulated time-dependent landing data for *T. urticae* exposed to C4 (points) with fitted trajectories from Probit, Hill2P, Hill4P, and GP models. Dashed vertical lines indicate ET_50_ estimates from each model, where available.


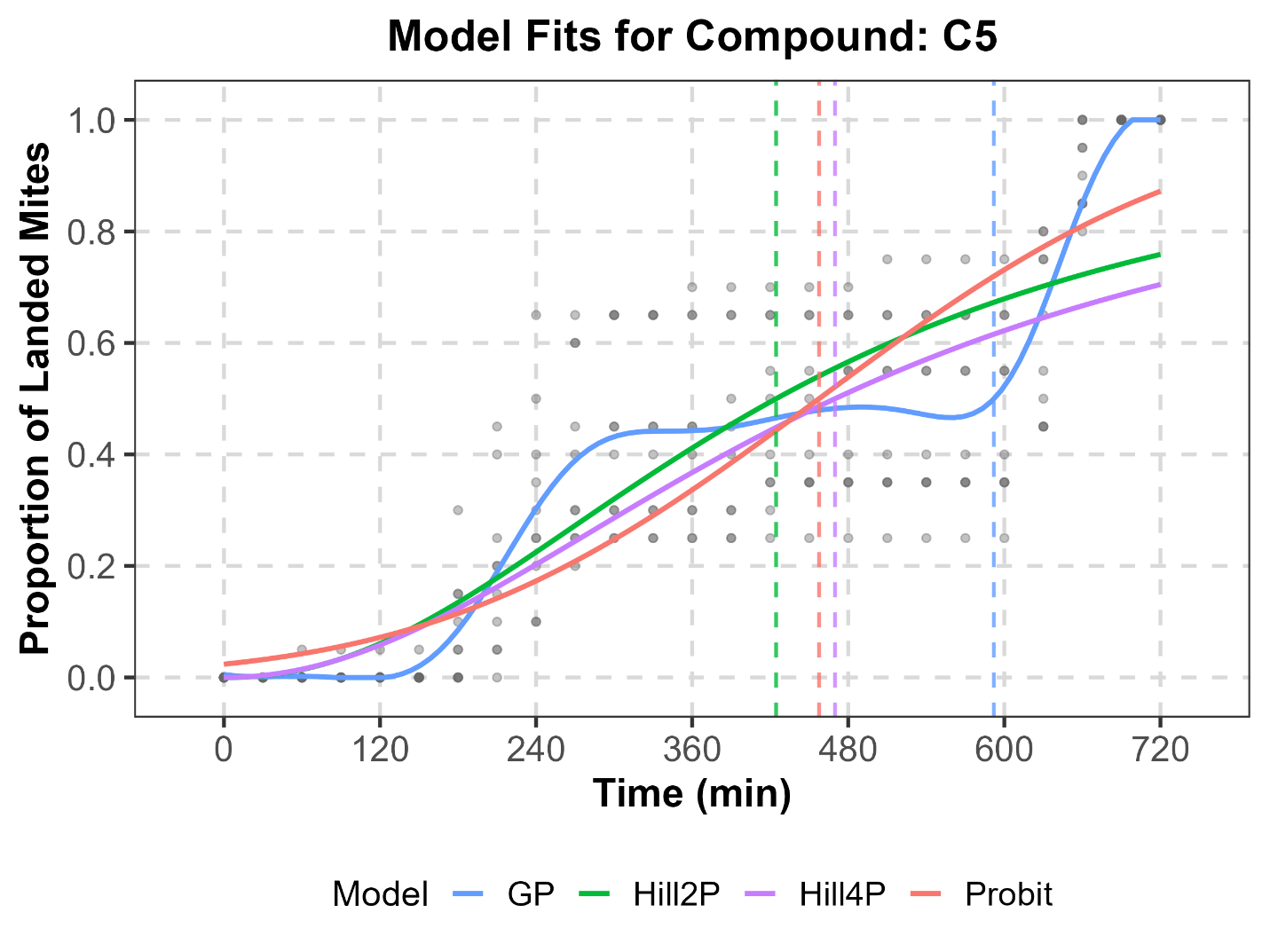


**Supplementary Fig. S35.** Comparison of simulated time-dependent landing data for *T. urticae* exposed to C5 (points) with fitted trajectories from Probit, Hill2P, Hill4P, and GP models. Dashed vertical lines indicate ET_50_ estimates from each model, where available.


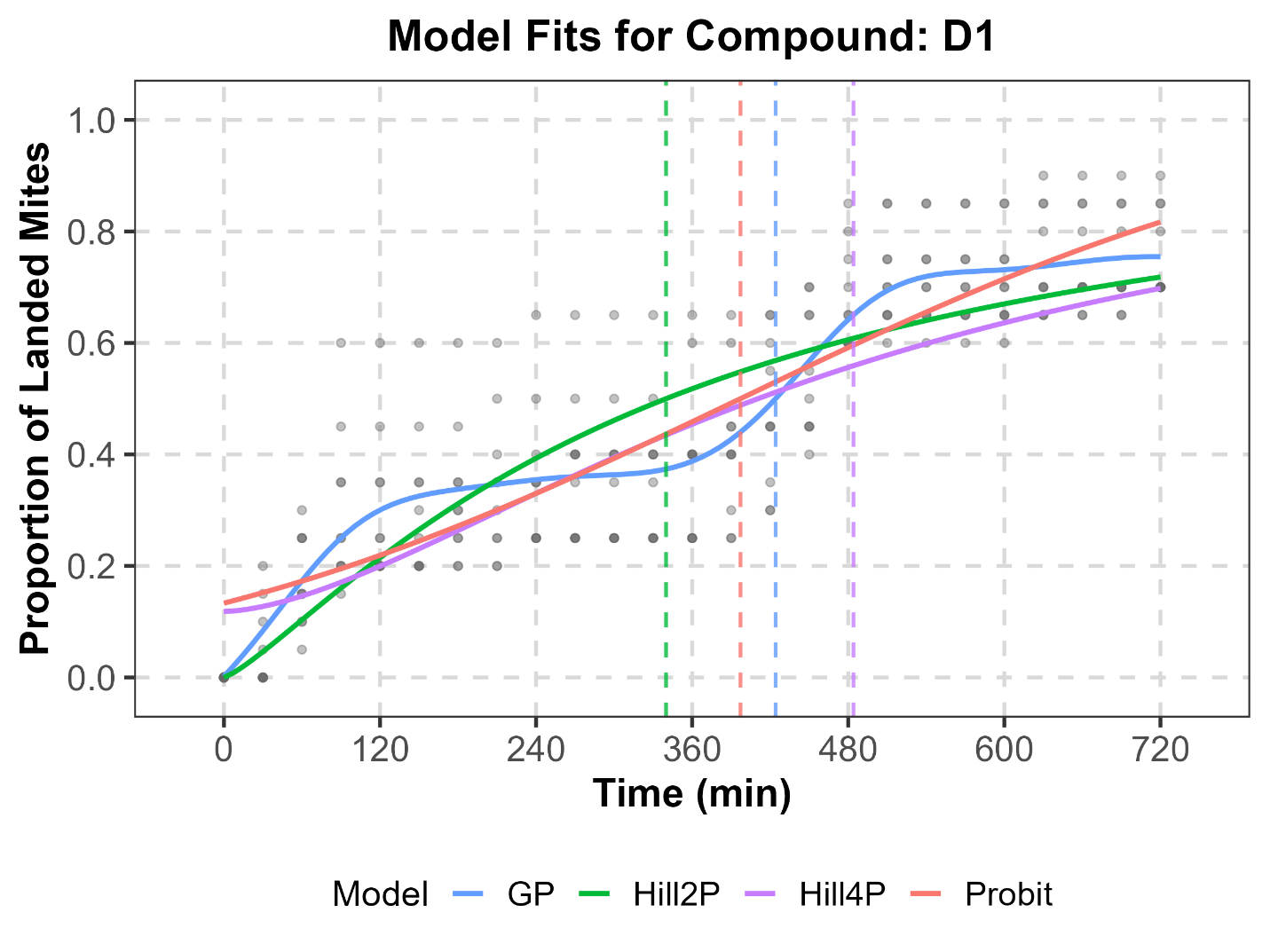


**Supplementary Fig. S36.** Comparison of simulated time-dependent landing data for *T. urticae* exposed to D1 (points) with fitted trajectories from Probit, Hill2P, Hill4P, and GP models. Dashed vertical lines indicate ET_50_ estimates from each model, where available.


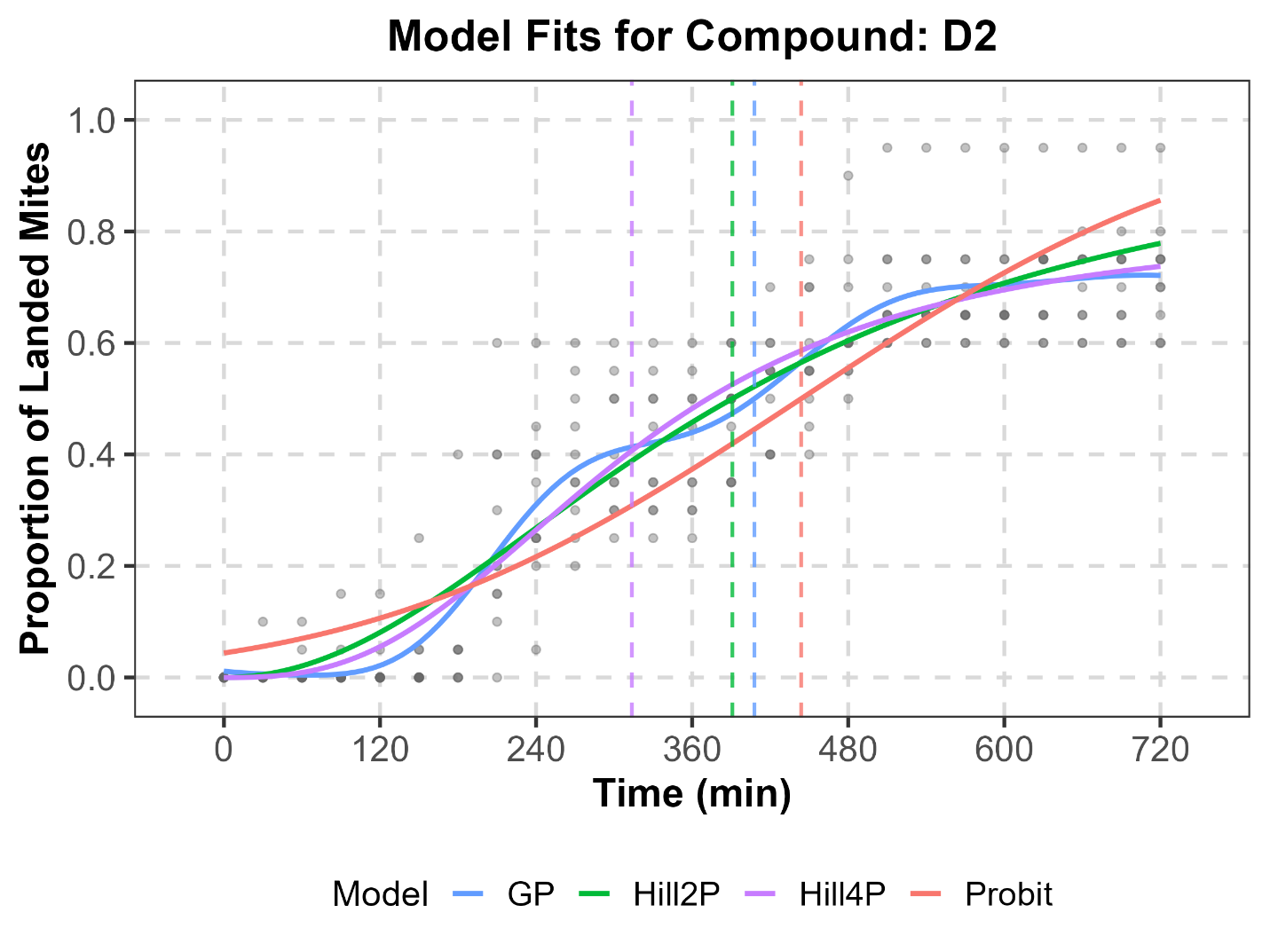


**Supplementary Fig. S37.** Comparison of simulated time-dependent landing data for *T. urticae* exposed to D2 (points) with fitted trajectories from Probit, Hill2P, Hill4P, and GP models. Dashed vertical lines indicate ET_50_ estimates from each model, where available.


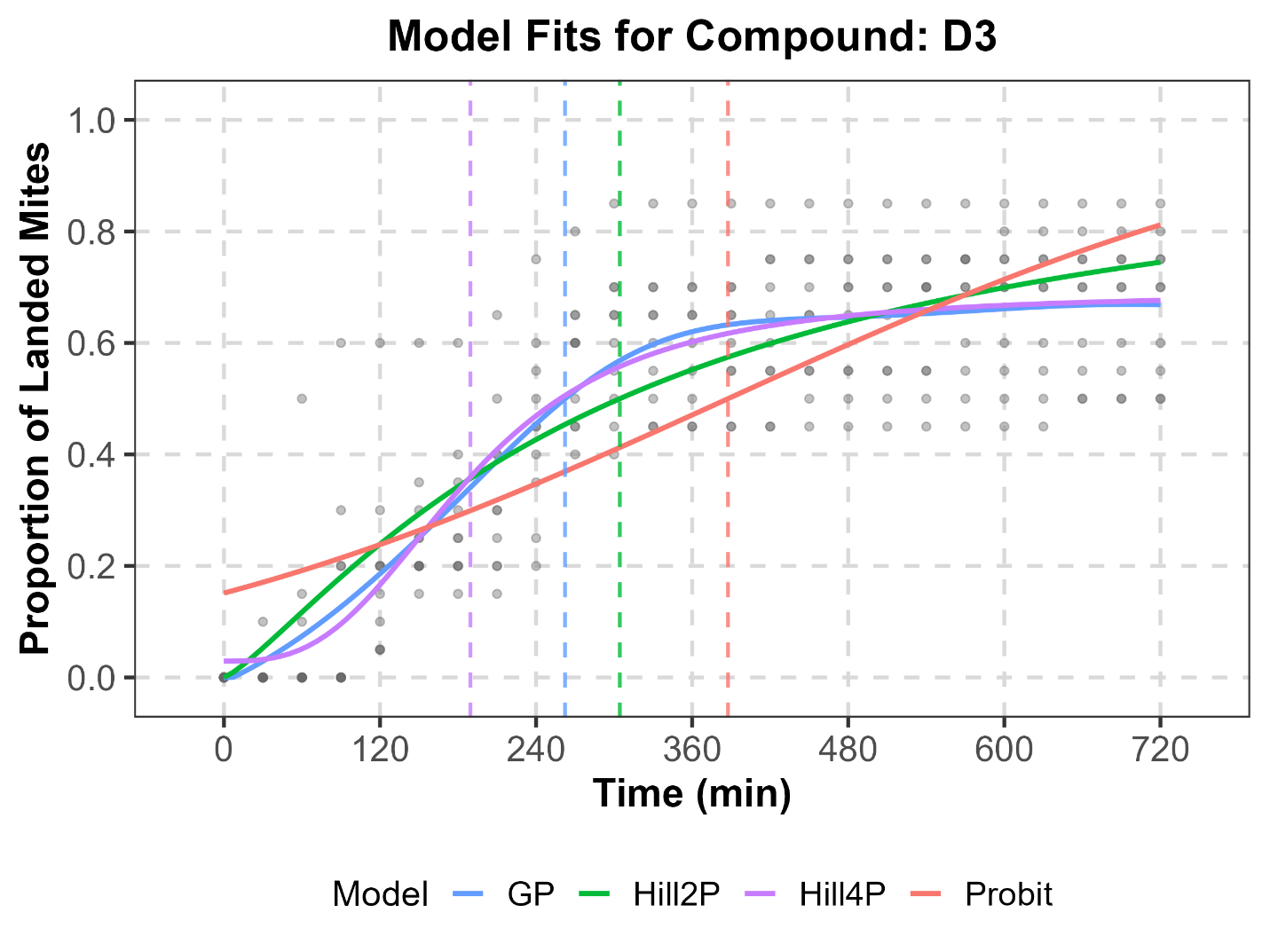


**Supplementary Fig. S38.** Comparison of simulated time-dependent landing data for *T. urticae* exposed to D3 (points) with fitted trajectories from Probit, Hill2P, Hill4P, and GP models. Dashed vertical lines indicate ET_50_ estimates from each model, where available.


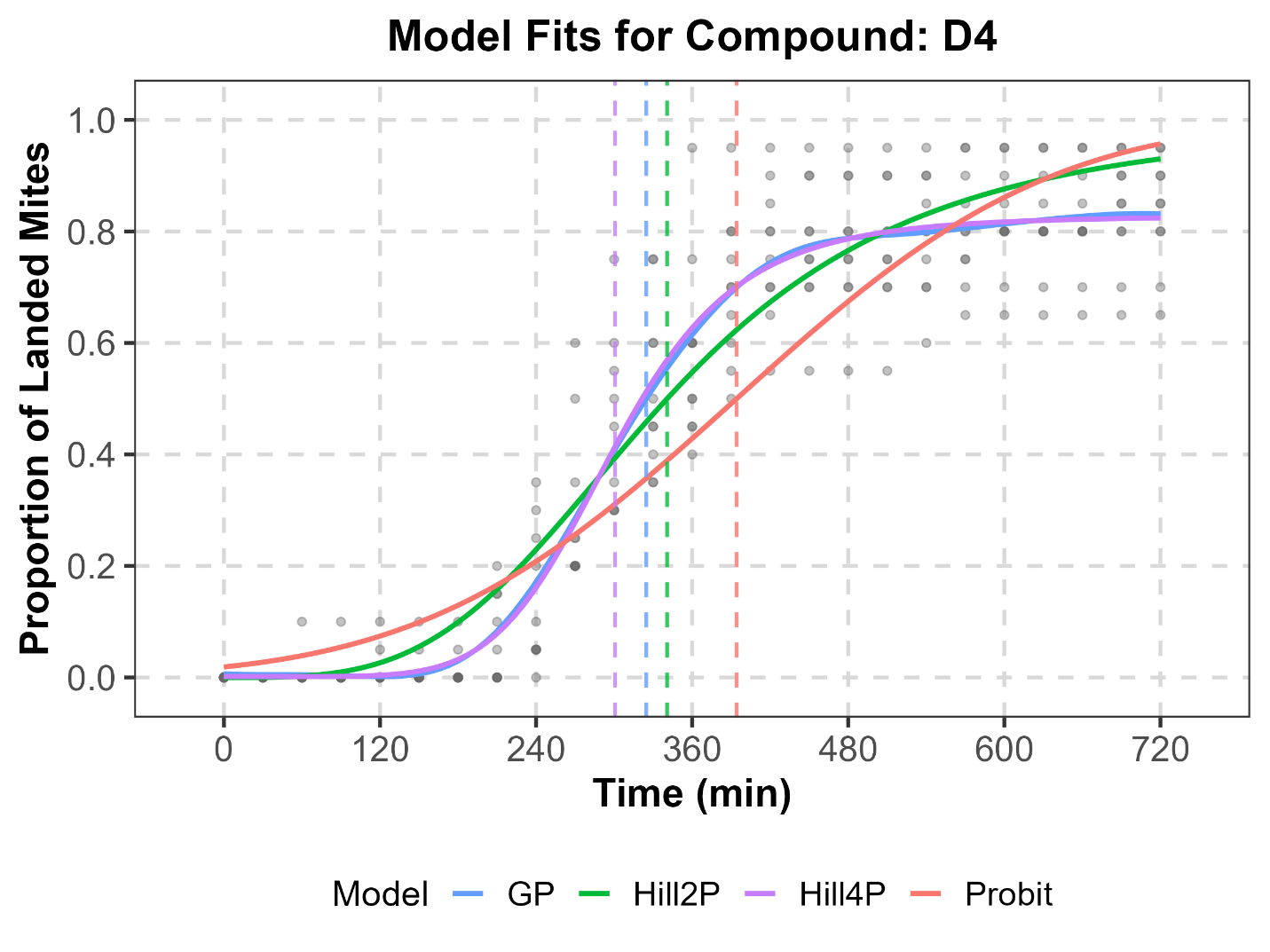


**Supplementary Fig. S39.** Comparison of simulated time-dependent landing data for *T. urticae* exposed to D4 (points) with fitted trajectories from Probit, Hill2P, Hill4P, and GP models. Dashed vertical lines indicate ET_50_ estimates from each model, where available.


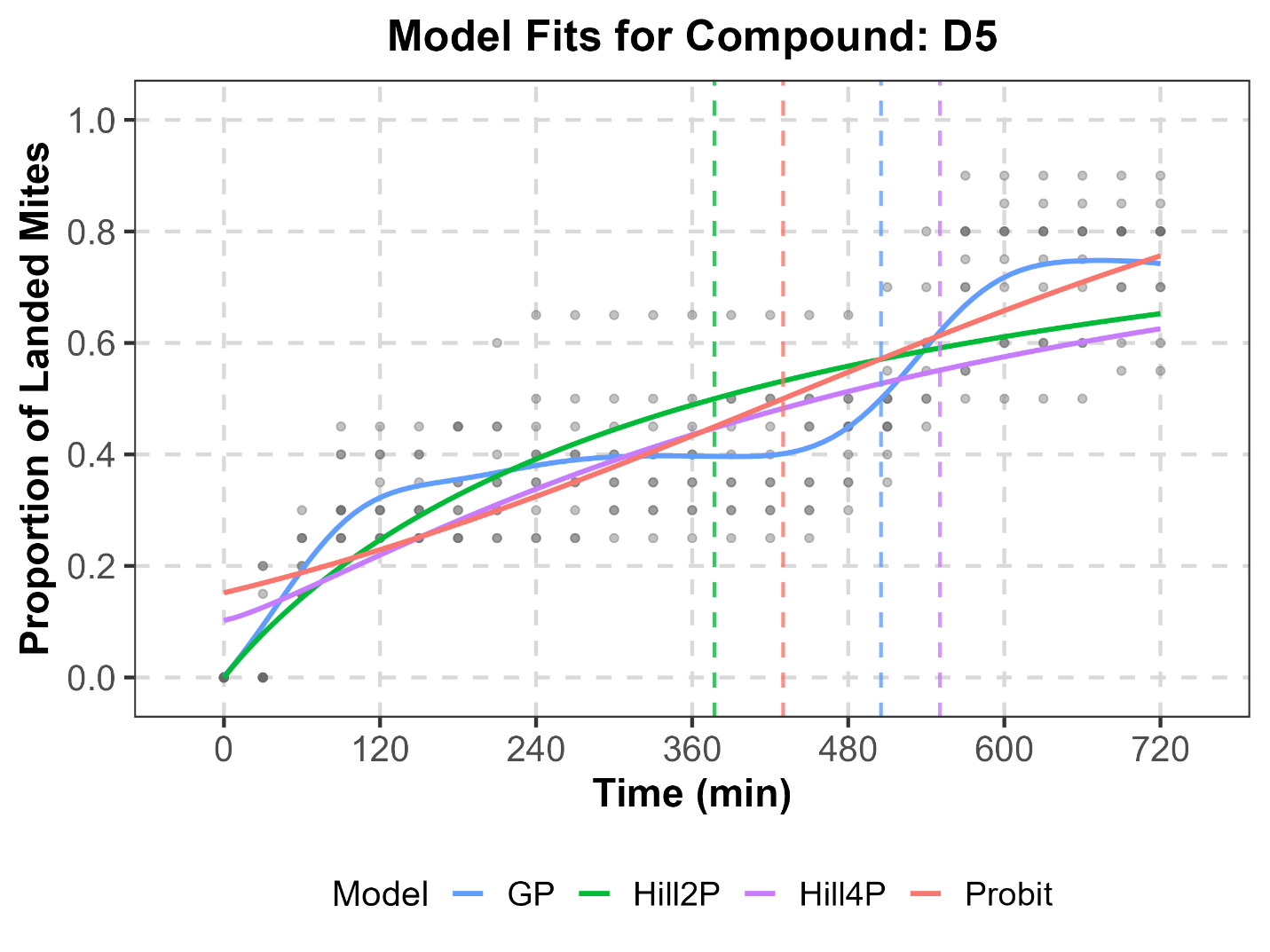


**Supplementary Fig. S40.** Comparison of simulated time-dependent landing data for *T. urticae* exposed to D5 (points) with fitted trajectories from Probit, Hill2P, Hill4P, and GP models. Dashed vertical lines indicate ET_50_ estimates from each model, where available.

**Supplementary Table S3.** Fit statistics, estimated ET_50_, and estimated AUC of synthetic data

| Compound | Model | RMSE | MAE | R^2^ | ET_50_ (min, 95% CI) | AUC (min, 95% CI) |
| --- | --- | --- | --- | --- | --- | --- |
| A1 | Probit | 0.092 | 0.063 | 0.937 | 214.873 (206.016 - 223.057) | 501.589 (479.962 - 501.628) |
| A1 | Hill2P | 0.069 | 0.049 | 0.964 | 184.404 (176.889 - 192.061) | 506.103 (484.754 - 506.32) |
| A1 | Hill4P | 0.069 | 0.049 | 0.964 | 186.041 (176.508 - 194.209) | 504.614 (483.251 - 504.858) |
| A1 | GP | 0.069 | 0.049 | 0.964 | 187.769 (177.226 - 198.37) | 507.92 (486.52 - 508.135) |
| A2 | Probit | 0.128 | 0.086 | 0.909 | 317.897 (305.712 - 329.857) | 401.948 (380.301 - 402.047) |
| A2 | Hill2P | 0.116 | 0.073 | 0.925 | 290.925 (280.452 - 306.314) | 411.267 (389.89 - 411.46) |
| A2 | Hill4P | 0.116 | 0.073 | 0.925 | 288.28 (273.57 - 302.405) | 408.792 (387.689 - 409.003) |
| A2 | GP | 0.116 | 0.073 | 0.925 | 291.494 (276.894 - 305.17) | 408.552 (387.344 - 408.729) |
| A3 | Probit | 0.097 | 0.028 | 0.946 | 548.535 (538.777 - 557.931) | 171.472 (149.658 - 172.327) |
| A3 | Hill2P | 0.099 | 0.033 | 0.945 | 546.871 (534.431 - 558.581) | 171.718 (150.118 - 172.386) |
| A3 | Hill4P | 0.099 | 0.033 | 0.945 | 548.134 (537.093 - 558.672) | 170.464 (148.558 - 171.109) |
| A3 | GP | 0.097 | 0.029 | 0.946 | 547.317 (531.325 - 559.26) | 172.107 (143.98 - 172.852) |
| A4 | Probit | 0.086 | 0.061 | 0.952 | 396.198 (389.402 - 404.939) | 324.207 (302.734 - 324.29) |
| A4 | Hill2P | 0.080 | 0.056 | 0.958 | 373.772 (366.095 - 381.961) | 326.545 (305.891 - 326.698) |
| A4 | Hill4P | 0.080 | 0.057 | 0.958 | 375.209 (362.636 - 390.934) | 327.889 (307.121 - 328.015) |
| A4 | GP | 0.080 | 0.057 | 0.958 | 377.437 (367.883 - 385.769) | 328.296 (307.823 - 328.406) |
| A5 | Probit | 0.108 | 0.085 | 0.901 | 428.833 (418.849 - 440.182) | 295.286 (275.112 - 295.341) |
| A5 | Hill2P | 0.099 | 0.076 | 0.918 | 394.394 (385.08 - 403.598) | 300.303 (281.261 - 300.397) |
| A5 | Hill4P | 0.098 | 0.077 | 0.918 | 379.533 (362.534 - 410.174) | 299.884 (281.2 - 299.962) |
| A5 | GP | 0.098 | 0.076 | 0.919 | 392.826 (382.224 - 404.754) | 299.988 (281.295 - 300.071) |
| B1 | Probit | 0.125 | 0.061 | 0.856 | 599.703 (588.405 - 613.444) | 122.539 (102.765 - 122.77) |
| B1 | Hill2P | 0.117 | 0.058 | 0.877 | 581.374 (567.786 - 595.748) | 136.494 (109.791 - 136.754) |
| B1 | Hill4P | NA | NA | NA | NA | NA |
| B1 | GP | 0.105 | 0.043 | 0.899 | 571.497 (555.365 - 589.047) | 128.877 (111.595 - 129.201) |
| B2 | Probit | 0.210 | 0.178 | 0.570 | 282.264 (247.035 - 307.48) | 416.907 (395.894 - 416.912) |
| B2 | Hill2P | 0.159 | 0.127 | 0.757 | 203.733 (187.007 - 222.801) | 442.745 (423.503 - 442.818) |
| B2 | Hill4P | 0.120 | 0.093 | 0.858 | 155.67 (145.796 - 168.677) | 427.577 (410.927 - 428.09) |
| B2 | GP | 0.121 | 0.094 | 0.857 | 176.532 (163.466 - 186.07) | 428.077 (411.523 - 428.479) |
| B3 | Probit | 0.131 | 0.094 | 0.870 | 470.228 (457.511 - 483.371) | 253.683 (233.532 - 253.765) |
| B3 | Hill2P | 0.101 | 0.071 | 0.928 | 423.426 (415.957 - 433.051) | 279.253 (259.273 - 279.376) |
| B3 | Hill4P | 0.059 | 0.038 | 0.974 | 377.076 (371.307 - 382.706) | 263.442 (246.651 - 263.73) |
| B3 | GP | 0.059 | 0.038 | 0.974 | 395.269 (387.81 - 401.745) | 263.36 (246.474 - 263.738) |
| B4 | Probit | 0.184 | 0.158 | 0.594 | 395.492 (373.43 - 420.626) | 334.601 (316.787 - 334.624) |
| B4 | Hill2P | 0.142 | 0.116 | 0.761 | 306.533 (286.862 - 326.553) | 354.315 (337.475 - 354.356) |
| B4 | Hill4P | 0.094 | 0.073 | 0.894 | 192.903 (184.063 - 202.461) | 343.672 (329.295 - 344.093) |
| B4 | GP | 0.094 | 0.074 | 0.893 | 225.311 (214.136 - 235.52) | 344.213 (330.067 - 344.58) |
| B5 | Probit | 0.100 | 0.067 | 0.865 | 568.514 (555.456 - 581.011) | 172.81 (156.047 - 172.901) |
| B5 | Hill2P | 0.090 | 0.064 | 0.891 | 553.311 (539.29 - 571.732) | 181.673 (166.248 - 181.746) |
| B5 | Hill4P | 0.086 | 0.059 | 0.900 | 453.749 (428.636 - 496.639) | 178.512 (164.326 - 178.601) |
| B5 | GP | 0.085 | 0.058 | 0.901 | 536.904 (509.935 - 573.081) | 177.215 (162.671 - 177.325) |
| C1 | Probit | 0.155 | 0.120 | 0.793 | 392.758 (376.81 - 408.085) | 330.422 (309.836 - 330.466) |
| C1 | Hill2P | 0.176 | 0.144 | 0.743 | 393.09 (370.521 - 422.182) | 299.077 (281.033 - 299.156) |
| C1 | Hill4P | 0.134 | 0.100 | 0.843 | 520.575 (503.947 - 528.675) | 322.569 (301.13 - 322.852) |
| C1 | GP | 0.089 | 0.069 | 0.930 | 507.999 (497.499 - 517.6) | 326.524 (305.424 - 327.113) |
| C2 | Probit | 0.146 | 0.103 | 0.835 | 257.728 (245.285 - 272.783) | 458.233 (436.239 - 458.27) |
| C2 | Hill2P | 0.172 | 0.137 | 0.803 | 269.516 (239.575 - 295.606) | 413.175 (392.643 - 413.317) |
| C2 | Hill4P | 0.125 | 0.084 | 0.880 | 372.932 (346.16 - 384.122) | 448.245 (426.138 - 448.583) |
| C2 | GP | 0.101 | 0.069 | 0.921 | 354.816 (340.274 - 372.027) | 454.228 (431.986 - 454.463) |
| C3 | Probit | 0.124 | 0.090 | 0.885 | 342.118 (330.387 - 354.386) | 377.337 (348.975 - 377.396) |
| C3 | Hill2P | 0.123 | 0.096 | 0.888 | 304.178 (290.151 - 322.843) | 373.851 (354.285 - 373.953) |
| C3 | Hill4P | 0.134 | 0.106 | 0.897 | 335.9 (323.68 - 349.002) | 342.699 (324.168 - 342.788) |
| C3 | GP | 0.089 | 0.059 | 0.941 | 333.612 (249.624 - 377.187) | 379.047 (357.408 - 379.489) |
| C4 | Probit | 0.139 | 0.102 | 0.846 | 346.008 (333.881 - 360.513) | 373.24 (351.933 - 373.28) |
| C4 | Hill2P | 0.146 | 0.115 | 0.830 | 310.304 (292.275 - 327.759) | 363.939 (345.012 - 364.048) |
| C4 | Hill4P | 0.154 | 0.127 | 0.843 | 358.371 (330.434 - 385.628) | 336.322 (317.927 - 336.384) |
| C4 | GP | 0.101 | 0.072 | 0.917 | 376.263 (329.362 - 417.63) | 372.985 (351.583 - 373.348) |
| C5 | Probit | 0.172 | 0.132 | 0.712 | 457.524 (436.985 - 480.199) | 275.161 (256.55 - 275.206) |
| C5 | Hill2P | 0.169 | 0.131 | 0.721 | 424.549 (399.059 - 451.5) | 277.931 (261.584 - 277.997) |
| C5 | Hill4P | 0.174 | 0.134 | 0.725 | 469.824 (437.559 - 499.949) | 252.976 (237.896 - 253.031) |
| C5 | GP | 0.125 | 0.092 | 0.848 | 591.929 (331.423 - 615.649) | 273.725 (252.085 - 274.21) |
| D1 | Probit | 0.123 | 0.098 | 0.755 | 397.136 (380.557 - 412.051) | 334.553 (316.113 - 334.57) |
| D1 | Hill2P | 0.130 | 0.098 | 0.729 | 339.954 (319.818 - 363.363) | 332.52 (317.044 - 332.537) |
| D1 | Hill4P | 0.133 | 0.104 | 0.746 | 484.031 (417.448 - 537.093) | 309.755 (294.777 - 309.785) |
| D1 | GP | 0.107 | 0.085 | 0.815 | 424.086 (407.089 - 439.924) | 338.178 (321.788 - 338.24) |
| D2 | Probit | 0.127 | 0.101 | 0.809 | 443.817 (429.006 - 459.562) | 290.722 (272.111 - 290.758) |
| D2 | Hill2P | 0.108 | 0.081 | 0.862 | 390.842 (376.912 - 406.269) | 300.312 (283.612 - 300.389) |
| D2 | Hill4P | 0.106 | 0.077 | 0.867 | 313.66 (283.179 - 360.001) | 297.898 (282.014 - 298.003) |
| D2 | GP | 0.101 | 0.075 | 0.879 | 407.806 (383.875 - 427.09) | 296.804 (280.945 - 296.982) |
| D3 | Probit | 0.166 | 0.136 | 0.605 | 387.549 (363.924 - 406.741) | 341.784 (323.326 - 341.798) |
| D3 | Hill2P | 0.137 | 0.108 | 0.732 | 304.344 (283.508 - 326.058) | 352.872 (336.791 - 352.891) |
| D3 | Hill4P | 0.129 | 0.103 | 0.761 | 189.441 (169.036 - 359.989) | 348.512 (333.654 - 348.606) |
| D3 | GP | 0.128 | 0.102 | 0.766 | 262.287 (236.164 - 281.192) | 349.294 (334.724 - 349.374) |
| D4 | Probit | 0.144 | 0.109 | 0.844 | 394.16 (382.511 - 409.896) | 327.859 (307.189 - 327.907) |
| D4 | Hill2P | 0.114 | 0.084 | 0.904 | 340.725 (329.456 - 351.759) | 348.848 (328.835 - 348.968) |
| D4 | Hill4P | 0.099 | 0.072 | 0.926 | 300.775 (291.415 - 314.385) | 337.264 (319.464 - 337.466) |
| D4 | GP | 0.099 | 0.071 | 0.926 | 324.712 (305.471 - 338.154) | 337.078 (318.986 - 337.256) |
| D5 | Probit | 0.125 | 0.104 | 0.691 | 429.919 (411.215 - 450.739) | 317.435 (300.181 - 317.449) |
| D5 | Hill2P | 0.131 | 0.107 | 0.660 | 377.153 (346.053 - 413.513) | 317.782 (303.197 - 317.753) |
| D5 | Hill4P | 0.135 | 0.110 | 0.673 | 550.545 (420.009 - 631.417) | 293.758 (280.326 - 293.769) |
| D5 | GP | 0.102 | 0.081 | 0.794 | 505.202 (491.417 - 522.753) | 320.106 (303.833 - 320.157) |
